# Growth promotion of maize and metabolomic profiling of *Azospirillum brasilense* cultures at varying incubation times

**DOI:** 10.1007/s42770-026-02134-0

**Published:** 2026-10-29

**Authors:** Amanda Neumann, Elisete Pains Rodrigues, André Luiz Martinez de Oliveira

**Affiliations:** 1 Departamento de Bioquímica e Biotecnologia, CCE, Universidade Estadual de Londrina, PO BOX 6001, Londrina, PR 86051-990 Brazil; 2 Departamento de Biologia Geral, CCB, Universidade Estadual de Londrina, Londrina, Brazil

**Keywords:** Plant-bacteria interaction, Bacterial bioinput, Maize inoculation, PGPB metabolites

## Abstract

*Azospirillum brasilense* Ab-V5, a well-known plant growth-promoting bacteria (PGPB), is widely used in commercial inoculant formulations for crops of economic and nutritional relevance. The mechanisms underlying its plant-growth-promoting activity are largely associated with secondary metabolic products such as exopolysaccharides (EPS), polyhydroxyalkanoates (PHA), phytohormones, siderophores, enzymes, and other bioactive metabolites. In this study, cultures of *A. brasilense* Ab-V5 at 12, 36 and 72 h (representing the exponential, stationary and decay growth phases) were sampled for untargeted metabolomic analysis using gas chromatography-mass spectrometry (GC–MS), and to evaluate inoculation responses in maize (*Zea mays*). After 15 days of growth in a greenhouse, the maize plants responded differently to inoculation depending on the growth stage of the bacterial inoculum, with the most pronounced increases in shoot dry weight and root system development observed in plants inoculated with 72-hour cultures. Metabolomic profiling revealed distinct metabolic patterns across growth phases, with a different expression of metabolites associated with physiological maturation and potential plant interaction capacity. Cultures from the 72-hour decaying stage displayed significant qualitative and quantitative shifts in metabolic compounds that were putatively assigned to carbohydrates, carboxylic acids, and aromatic and phenolic compounds. The role of putatively assigned metabolites on inoculation efficiency and the plant response is discussed.

**Supplementary Information:**

The online version contains supplementary material available at https://doi.org/10.1007/s42770-026-02134-0.

## Introduction

Bacteria of the genus *Azospirillum* are widely recognized as plant growth-promoting bacteria (PGPB), with several species integrated into commercial inoculant formulations. These microorganisms enhance plant growth through both direct and indirect mechanisms, including improved nutrient uptake and assimilation, modulation of plant morpho-physiological processes, and induction of tolerance to biotic and abiotic stress [1, 2]. Secondary metabolites produced by *Azospirillum* play essential roles in these mechanisms, for example phytohormones, organic acids, lipopolysaccharides (LPS), exopolysaccharides (EPS), and polyhydroxyalkanoates (PHA) [3]. Indeed, the performance of inoculants formulated with *Azospirillum* demonstrates a strong influence from the physiological quality of the microorganism, which in turn reflects characteristics of its metabolism that are determined by the cultivation conditions, nutrient composition of the culture medium, and the presence of bioactive compounds, as previously demonstrated [3–5].

Despite the extensive scientific literature on the physiology, ecology and application of *Azospirillum* as a biological input, the molecular-level interaction mechanisms between this genus of plant growth-promoting bacteria (PGPB) and plants are still not well understood [6]. This interaction begins with chemotaxis events, followed by *Azospirillum* migrating to the plant surface (usually the rhizoplane), establishing biofilms with active populations and beginning the mutualistic relationship with the plant [1, 3]. Recent studies using ‘omics’ tools have made important advances in our understanding of the relationship between *Azospirillum* and the host plant. These studies indicate that metabolic functions related to the metabolism of sugars, amino acids, organic acids and phenolic compounds play a role in inoculation efficiency and interaction with the native soil microbiota [7–10]. Recent studies using ‘omics’ tools have improved our understanding of the relationship between *Azospirillum* and the host plant. These studies suggest that metabolic functions related to the metabolism of sugars, amino acids, organic acids and phenolic compounds play a role in inoculation efficiency and interaction with the native soil microbiota. Furthermore, a recent meta-analysis of *Azospirillum*-based inoculants applied on extensive crops highlighted the variability in the inoculation response across crops, environmental conditions, and inoculation strategies, although the mean increase of grain yield on inoculated crops was about 10% over uninoculated controls [11].

The physiological state of *Azospirillum* in inoculant formulations has long been recognized as an important trait for inoculation efficacy [12, 13]. In a previous work, a culture medium designed to increase the EPS and PHA production by *A. brasilense* Ab-V5 has shown that the bacterial growth period (e.g. growth phase) is also an important determinant of the inoculation response [5]. In this context, the present study aimed to characterize the metabolomic profile of *Azospirillum* in an optimized culture medium to improve inoculation performance, in order to understand the bacterial metabolic dynamics throughout different growth phases, and search for metabolites that could influence the physiological state of this microorganism and its interaction with the host plant.

## Materials and methods

### Preparation of pre-inoculum and *A. brasilense* Ab-V5 cultures

The bacterial strain *Azospirillum brasilense* Ab-V5 was kindly provided by the Diazotrophic and Plant Growth-Promoting Bacteria Culture Collection of Embrapa Soja (collections WFCC #1213, WDCM #1054). This strain is widely used in commercial inoculant formulations in Brazil. The pre-inoculum was prepared by growing *A. brasilense* Ab-V5 in Dygs medium [14] at 28 °C and 180 rpm for 24 h. The culture was then adjusted to 1 × 10⁷ cells mL⁻¹ using sterile Dygs medium, and 100 µL aliquots were used to inoculate 100 mL of FORM15 medium [5]. The medium composition of FORM15, for one liter, consists of 30 g of glycerol, 50 g of sucrose, 50 g of yeast extract, 1.5 g K_2_HPO_4_, and 0.1 mL of micronutrient solution (g L^− 1^: H_3_BO_3_, 1.4; ZnSO_4_.7H_2_O, 1.2; MnSO_4_.H_2_O, 1.18; Na_2_MoO_4_.2H_2_O, 1.0; CuSO_4_.5H_2_O, 0.04). Cultures were incubated at 28 °C and 180 rpm. Flasks containing uninoculated FORM15 medium were maintained under identical conditions and used as the control.

### Experimental design

Bacterial cultures were grown in a completely randomized experimental design with three replicates. Samples (50 mL) were collected after 12, 36, and 72 h of incubation, corresponding to the exponential, stationary and decay phases, respectively, according to previous data [5]. The mean population counts of *A. brasilense* Ab-V5 in FORM15 at the indicated sampling times were as follows: 7.47 CFU mL^− 1^ (12 h incubation), 9.66 CFU mL^− 1^ (36 h incubation), and 8.27 CFU mL^− 1^ (72 h incubation). These bacterial cultures were subsequently used to inoculate maize seeds and undergo GC–MS metabolomic analysis, as described in Sect. Gas chromatography-mass spectrometry (GC–MS) of A. brasilense cultures. The inoculation experiment was conducted under greenhouse conditions for just 15 days, as inoculation with *A. brasilense* has previously been shown to promote early maize growth [15]. The trial followed a completely randomized experimental design with nine replicates (pots) per treatment, with one plant per pot. At the end of the 15-day period, the nine replicates were pooled into three composite samples per treatment to increase accuracy in the biometric analyses. Each composite sample was considered as one replicate, and the results are presented as mean values relating to a single plantlet.

### Plants and *A. brasilense* Ab-V5 inoculation

Maize (*Zea mays* L. cv. BRS 2107, Embrapa) seeds were pregerminated for 48 h at 28 °C. Uniform seedlings were transplanted into 0.5 L pots containing 0.5 kg of sterilized sand–soil mixture (3:1 w/w). The substrate had the following characteristics: pH (in H_2_O), 5.8; H + Al (cmol_c_ dm^− 3^), 4.26; K (cmol_c_ dm^− 3^), 3.77; Ca (cmol_c_ dm^− 3^), 4.3; Mg (cmol_c_ dm^− 3^), 3.1; Al (cmol_c_ dm^− 3^), 0.18; P (mg dm^− 3^), 17.0; organic matter (%), 1.78. Immediately after transplanting, the plants were inoculated with 1.0 mL of *A. brasilense* suspensions (12, 36, and 72 h cultures, adjusted to 1 × 10⁷ cells mL⁻¹). Plants receiving 1.0 mL of uninoculated FORM15 medium were used as controls.

### Gas chromatography-mass spectrometry (GC–MS) of *A. brasilense* cultures

The GC–MS was used to access the low-molecular-mass and thermally stable compounds secreted by *A. brasilense* Ab-V5 into the FORM15 medium. To this, aliquots of the cultures and control treatments were collected in triplicate at the previously defined incubation times, adjusted to a population density of 1 × 10⁷ cells mL⁻¹ and centrifuged for 10 min at 4,000 g to allow the cells and/or particles to sediment. Then, 30 mL from the culture supernatants were filtered (porosity of 0.22 μm), and lyophilized. The dried material was reconstituted in 10 mL of ultrapure water and 5 mL of this solution were subjected to solid-phase extraction (Strata-X^®^, 200 mg/3 mL; Phenomenex), following the manufacturer’s instructions (conditioning, sample loading and elution). Metabolites were eluted with 1.2 mL methanol (MS grade), concentrated under vacuum using the miVac Duo (Genevac), and stored at -80 °C until analysis.

Prior to GC–MS analysis, the samples were resuspended in 1 mL acetonitrile, sonicated (28 kHz, 600 W, 10 min, 30 °C), and derivatized sequentially with methoxyamine in pyridine (20 mg mL⁻¹, 1 h, 80 °C) and N-methyl-N-(trimethylsilyl)trifluoroacetamide (MSTFA, 37 °C, 30 min). The samples were then filtered (porosity of 0.22 μm) and 1 µL aliquots of the derivatized samples were subjected to GC-MS analysis using the equipment GC-MS QP2010SE (Shimadzu) with RTX-5MS RESTEK column (30 m × 0.25 mm, 0.2 μm film). Helium was the carrier gas (1.0 mL min⁻¹). The injector temperature was maintained at 270 °C, and the column was maintained at an initial temperature of 70 °C for 2 min, with a gradual temperature increase of 40 °C min⁻¹ until reaching 320 °C, at which the temperature was maintained for 1 min. The temperatures of the transfer line and the ion source were 250 °C and 200 °C, respectively. Ions were generated by a 70 eV electron beam at an ionization current of 2.0 mA, and 30 spectra/second were recorded in the mass range of 40–700 m/z.

The stability of the GC-MS equipment was evaluated by injecting quality control (QC) samples, prepared by mixing equal volumes of all the samples (culture and control treatments), and treated as described above. The QC samples were injected into the instrument, interspersed every eight test samples (cultures and controls).

### Biometric analysis of inoculated plants

After 15 days, plants were gently removed from the pots, washed, separated into shoots and roots, and pooled to create the composite samples described above. Roots were scanned on a dark background, and images were analyzed using *GiA Roots* software [16] to determine total root length, mean diameter, and root area. Shoots and roots were oven-dried (40 °C, 72 h) for dry biomass determination. Biometric values were expressed as means per plant.

### Untargeted metabolomics analyses

The raw data obtained from the GC-MS analysis of the samples were transformed into .mzML format using the GCMSsolution program (v. 4.11; Shimadzu, Kyoto, Japan). The processing of the chromatograms and the extraction of molecular features were conducted using the XCMS package (v. 3.21) [17] along with the R platform (v. 4.3.0). All GC-MS spectra were corrected for retention index using the endogenous signals from all samples when processing chromatograms with the XCMS package on the R platform. The parameters applied included feature detection (peak picking) using the Matched Filter method (binSize = 0.1, fwhm = 5, steps = 2, max = 500, snthresh = 2, mzdiff = 0.5, index = F). The data recovered from the chromatograms were grouped by treatment (minSamples = 1, bw = 10, binSize = 0.05, minFraction = 0.5, maxFeatures = 50), corrected for retention time (minFraction = 0.5, family = “gaussian”, smooth = “loess”, span = 0.4) and grouped again (minSamples = 2, bw = 1, binSize = 0.05). These analyses resulted in a list of 783 peak intensities (a combined list of control and culture samples), with missing values (NA) filled in for signals present in some of the analyzed samples.

### Statistical analysis and compound identification

The results of the biometric analyses of the maize plants were subjected to normality analysis (Shapiro-Wilk) and homogeneity of variance analysis (Batlett) prior to analysis of variance (ANOVA). When significant differences were identified between treatments, comparisons between means were conducted using the Tukey’test (*p* < 0.05), with the aid of the ExpDes package [18], in the R environment (www.r-project.org).

For metabolomics analyses, the data matrix was subjected to statistical analysis using the MetaboAnalyst 6.0 platform (https://www.metaboanalyst.ca) [19]. Prior to analysis, the data matrix was pre-processed to fill in missing values using the PPCA algorithm, exclude low-quality signals (filters for low repeatability, low variance, and low signal intensity), and normalize the signals based on log10 base transformation and auto-scaling. Multivariate (PCA and sPLS-DA), univariate statistical approaches, and cluster analysis were performed using the MetaboAnalyst platform (ANOVA, fold change, t-test).

The pathway analysis module was used to identify putative metabolic pathways that were upregulated during Ab-V5 culturing, using the annotated compounds that were significantly more abundant in the inoculated FORM15 (Azo treatment) than in the uninoculated FORM15 (Cont treatment), as indicated by the t-test (one-way ANOVA, *p* < 0.05) and considering all sampling timepoints. The PCA model showed R² > 0.5 and a p-value < 0.05, indicating the model’s reliability. The sPLS-DA (sparse PLS-DA) analysis was performed considering 4 components (after model performance analysis by cross-validation) and 50 principal components for each dataset. Hierarchical clustering analysis was obtained using heatmaps with Euclidean correlations and Ward’s clustering algorithm.

Temporal analyses of Ab-V5 during cell cycle progression were determined by comparing the matrices obtained for each culture time with their respective controls to identify differentially expressed metabolites (*p* < 0.05) between samples with a fold change of at least two times. Contrasts between control treatments (Cont) x culture (Azo) at 12, 36, and 72 h were considered. Contrasts between the 12 h Azo x 36 h Azo and 36 h Azo x 72 h Azo culture treatments were also evaluated. The probable identification of differentially expressed compounds validated as variables of importance in the projection (VIP scores > 1.2) was performed using the National Institute of Standards and Technology (NIST) mass spectrum library.

Chromatogram deconvolution was performed using AMDIS v.2.73 software (NIST - Gaithersburg, MD, USA) and the NIST’08 MS database (NIST; Gaithersburg, MD, USA). Identification of selected metabolites was performed by combining retention times and mass fragmentation patterns, as recommended by the Metabolomics Standards Initiative (MSI) [20]. Since no standard compound was used to quantify the identified peaks, and since the retention index was corrected using the endogenous signals of the samples, the compound annotations were limited to the putative level. Probable compound annotation was performed based on the similarity of fragmentation patterns to those of NIST library entries when mass spectral comparison resulted in a Match Factor > 700 (lower Match Factor values were not considered). Features present in only one sample analyzed for a given treatment and features corresponding to artifacts were excluded from the analyses.

## Results

### Maize response to *A. brasilense* Ab-V5 inoculation

**Fig. 1:**
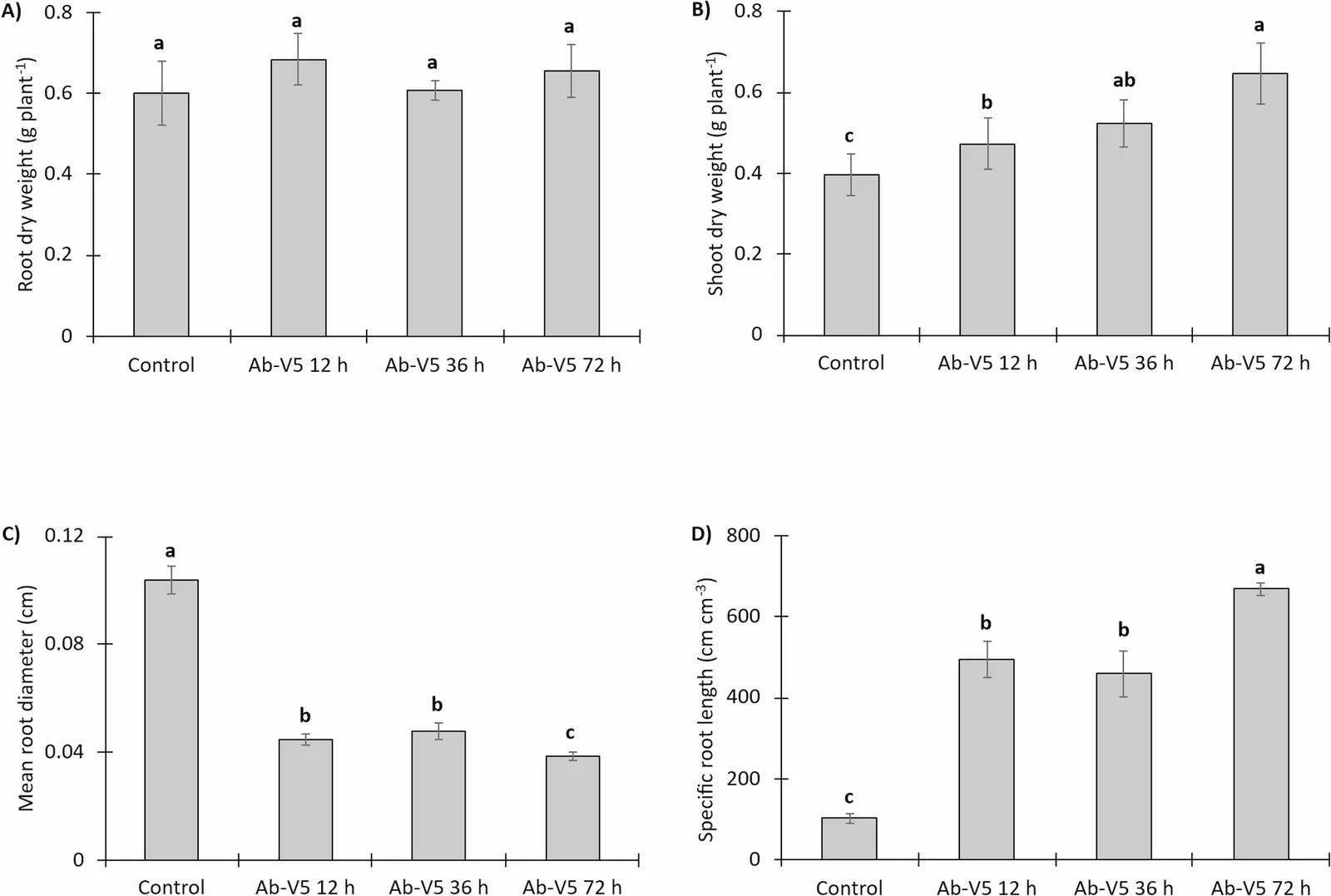
Biometric parameters of maize inoculated with *Azospirillum brasilense* Ab-V5 cells at different culturing times, after 15 days of growth under greenhouse conditions. (**A**) Root dry weight (g plant^-1^). (**B**) Shoot dry weight (g plant^-1^). (**C**) Mean root diameter (mm plant^-1^). (**D**) total root length (mm plant^-1^). Note: ‘Control’ indicates plants inoculated with sterilized FORM15 culture medium. ‘Ab-V5 12 h’, ‘Ab-V5 36 h’ and ‘Ab-V5 72 h’ indicate plants inoculated with A. brasilense Ab-V5 cultured in a FORM15 medium for increasing periods of time. Different lowercase letters above the bars indicate statistically significant differences (p < 0.05) according to the Tukey’s test

The response of maize plants to inoculation with *A. brasilense* Ab-V5 was influenced by the microorganism’s cultivation time and, consequently, its physiological state (Fig. 1). Although the dry weight of the roots showed no effect of the treatments (Fig. 1A), notable increases in the dry weight of the aerial parts of the plants were observed when longer cultivation times of Ab-V5 were used to prepare inoculant suspensions (Fig. 1B). Inoculation with Ab-V5 was also found to influence root architecture, reducing the root diameter of inoculated seedlings while increasing the specific root length (the product of the root length divided by the root volume; Fig. 1C and D).

### Untargeted metabolomics of *A. brasilense* Ab-V5 cultures at different incubation times

**Fig. 2:**
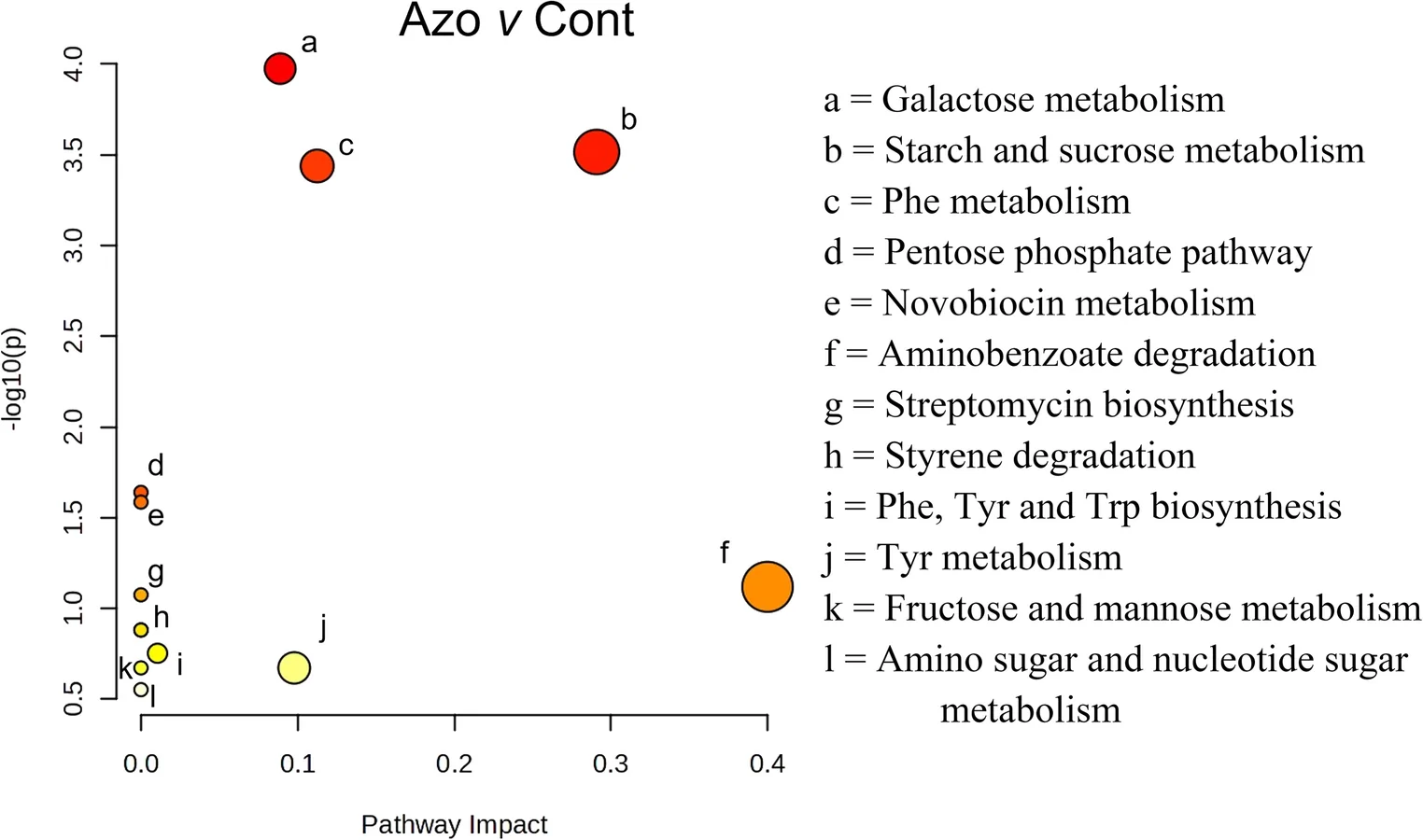
Metabolic pathways analysis of the up-regulated compounds in *A. brasilense* Ab-V5 revealed significantly higher abundances than observed in the culture medium without inoculation (one-way ANOVA, *p* < 0.05), considering all samples and timepoints. Each dot point represents a single metabolic pathway of *A. brasilense* Ab-V5, as identified through the pathways referenced in MetaboAnalyst

The GC-MS analysis of the FORM15 culture medium over an increasing incubation period yielded multiple signals (Figure S1). Overlay analysis of inoculated and uninoculated samples over the same incubation periods showed minor variations in retention time and peak intensity, suggesting minimal instrument-induced error. A one-way ANOVA of all the Azo and Cont samples at all timepoints revealed 65 chromatographic peaks of varying abundance, from which 60 were present at a higher intensity in the Azo samples and five in the Cont samples (Table S1). Of these compounds, 37 had a Match Factor value greater than 700, as indicated by the NIST’08 database. Of these, 34 were more abundant in the Azo samples and were potentially involved in 12 metabolic pathways, six of which had relevant impact values (Fig. 2). Secondary metabolites such as benzoate-related phenolic compounds, terephthalic acid, pantoyl lactone, and carbohydrates, among others, were the putative compounds identified as being significantly more abundant (Table S1, Fig. 2). 

**Fig. 3:**
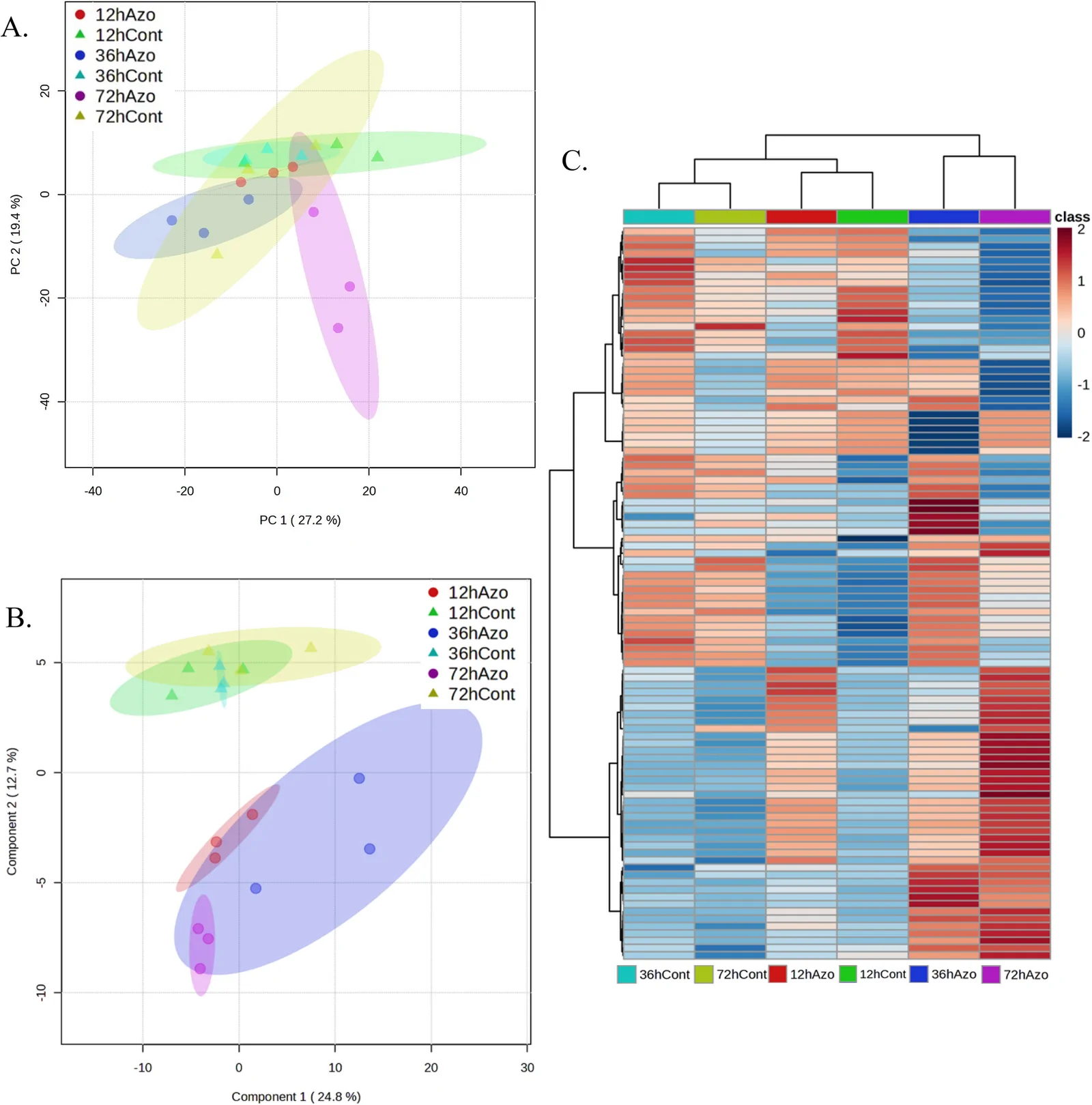
Multivariate analysis based on the area of the chromatographic peaks of the identified metabolites in the FORM15 culture medium inoculated (Azo) and uninoculated (Cont) with *A. brasilense* Ab-V5 and incubated at different times. (**A**) Principal component analysis (PCA) of the identified metabolites in each treatment. (**B**) Supervised discriminant analysis by partial least squares (sPLS-DA) for the top 50 metabolites in each treatment. (**C**) Hierarchical heatmap showing the clustering of the top 100 metabolites (heatmap rows) as defined by ANOVA for the treatments as groups (heatmap columns), using Ward’s algorithm and Euclidean distance.*Note*: Translucent ovals surrounding data points in (**A**) and (**B**) indicate 95% confidence intervals per treatment; variance of each PC is detailed on the respective axes

Figure 3 shows a comparison of 781 chromatographic peaks from the untargeted metabolomes of the Azo and Cont treatments at different culture times. Of these, 542 peaks were detected in at least two Cont replicates, and 501 in at least two Azo replicates. Principal component analysis (PCA) of this comparison did not clearly separate the treatments (Fig. 3A), probably due to the chemical complexity of the FORM15 medium, which resulted in a large number of common peaks across all samples. However, the position of the 72-hour Azo samples, which are almost fully separated from the other samples by PC1 and PC2, suggests the presence of several discriminating peaks.

Conversely, the metabolomic profiles of the inoculated (Azo) and uninoculated (Cont) samples were successfully discriminated through supervised partial least squares discriminant analysis (sPLS-DA), based on the 50 main variants of each sample (Fig. 3B). In this analysis, the 36-hour Azo has a wider confidence interval and overlaps with the 12-hour Azo and 72-hour Azo, suggesting a metabolic transition state between exponential growth (12 h of culturing) and the decay phase (72 h of culturing).

A heat map based on the top 100 most significant metabolites (Table S2), according to the ANOVA, is represented by Fig. 3C. The samples were clustered into three main groups, with the group I contained all the uninoculated samples as well as the 12-hour Azo sample, group II contained the 36-hour Azo sample and group III contained the 72-hour Azo sample. Taken together, the results in Fig. 3 suggest that the 72-hour Azo metabolome is clearly different from those obtained for shorter incubation period, and that a greater diversity of differentially expressed metabolites is observed during the decaying growth phase. Furthermore, given the improved maize response to inoculation when the Ab-V5 inoculum at the decaying growth phase was used (Fig. 1), one can also expect that the bacterial metabolites that are important for improved inoculation performance are more likely to be found in the 72-hour Azo metabolome. 

**Fig. 4:**
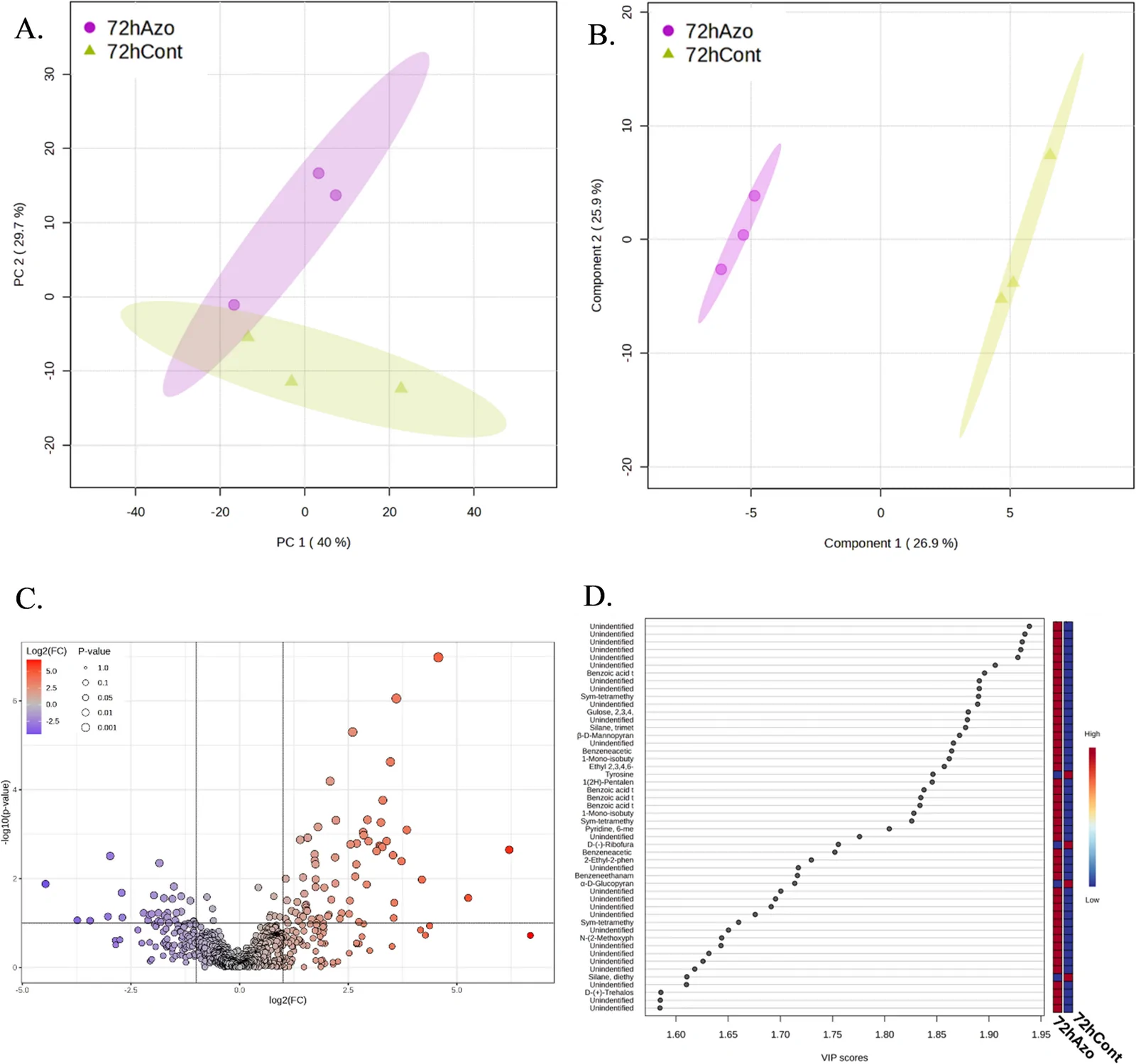
Comparative metabolome profiling of *A. brasilense* Ab-V5 inoculated (72hAzo) versus uninoculated (72hCont) FORM15 culture medium after 72 h of incubation under orbital shaking at 180 rpm and 28 °C. (**A**) Principal component analysis (PCA) of all metabolites recorded from the 72hAzo and 72hCont treatments. (**B**) Supervised discriminant analysis by partial least squares (sPLS-DA) for the top 50 metabolites from the 72hAzo and 72hCont treatments. (**C**) Volcano plot of the fold change analysis comparing the metabolomes of the 72hAzo and 72hCont, with a P-value cutoff of < 0.05 and log fold change (inoculated/uninoculated) cutoffs of > 1 or < − 1. (**D**) Variable importance in projection (VIP) score for the top 50 contrasting metabolites between the 72hAzo and 72hCont treatments

Based on these previous results, the Azo and Cont samples incubated for 72 h were selected for further analysis. The PCA of these samples comprised 526 chromatographic peaks, and the plot showed a small overlap between 72hAzo and 72hCont at 95% confidence intervals, and 69.7% of the variance was explained by PC1 and PC2 (Fig. 4A). Adding a third axis (PC3 which explained a further 13.5% of the variance) resulted in a clear separation of inoculated and uninoculated samples (Figure S2). The analysis of the top 50 variables from the 72hAzo and 72hCont treatments by a supervised sPLS-DA (95% confidence interval) clearly highlighted differences in the metabolomic profiles of these samples (Fig. 4B).

In an attempt to identify the relevant chromatographic peaks resulting from the Ab-V5 metabolism, a fold change (FC) analysis was carried out by contrasting the 72-hour Azo metabolome with the 72-hour Cont metabolome. Peaks with an FC value greater than two (log FC > 2.0; *p* < 0.05) were searched for and selected, yielding a total of 71 molecules with significantly higher intensity (i.e. positively regulated metabolites), as well as 30 molecules with significantly lower intensity in the 72-hour Azo metabolome (Fig. 4C, Table S3). Additionally, 46 of the 50 main discriminating metabolites, as determined by the variable importance in projection (VIP) score, were present in the 72-hour Azo metabolome (Fig. 4D). This finding is consistent with the results of the ANOVA and fold change analyses (Tables S1 and S3, respectively).

Searching these metabolites against the NIST’08 database using a Match Factor value > 700 (fair matches and above) allowed us to putatively assign identities to 37 of the differentially expressed metabolites. Of these compounds, eight metabolites were found to have a reduced peak intensity, while 29 were found to be positively regulated. The compounds that were positively regulated in the 72-hour Azo samples were putatively assigned to the following categories: carbohydrates, carboxylic acids, aromatic compounds, alcohols, fatty acids and other compounds (see Table S3). 

## Discussion

This study employed an untargeted metabolomics approach to perform an exploratory analysis and identify differentially expressed compounds in the exometabolome of *A. brasilense* Ab-V5. The analysis used a culture medium designed to enhance inoculation response and efficiency. The main goal was to lay the groundwork for future research aimed at enhancing the beneficial interaction between PGPB and plants. Since no chemical standards were used, absolute quantification and unequivocal identification were not possible. The decision to use the FORM15 culture medium to study the exometabolome of *A. brasilense* Ab-V5 was based on previous studies demonstrating the improved physiological quality and inoculation performance of cells cultivated in this medium [5, 21]. Although the composition of FORM15 could lead to the misidentification of compounds produced by *A. brasilense*, we argue that the likelihood of bias would be the same for any other culture medium, given that the phenotypic variability of bacteria in response to nutrient availability is well documented in the literature [22]. In this study, misattribution of compounds to the AbV5 metabolism was prevented by using uninoculated FORM15 as control and by only considering compounds with significantly different abundances in the analyses.

In maize seedlings, changes in the partitioning of carbon between roots and shoots, as well as growth stimuli or impairment, are often associated with alterations to the endogenous hormonal balance. These shifts can be triggered by biotic factors, such as PGPB inoculation, or abiotic factors, such as drought [15, 23, 24]. When considering *Azospirillum* inoculation, the changes observed in maize development are primarily attributed to the production of the phytohormone indole acetic acid by the associative bacteria [1, 3, 25]. Environmental and physiological conditions also affect how plants develop a beneficial associative relationship with growth-promoting microorganisms. For example, the method and timing of inoculation, and the availability of cotyledonary reserves, play a significant role in how the plant responds to PGPB inoculation [21, 26]. Previous metabolomic studies have shown that inoculating maize with *Azospirillum* modifies the set of identified plant compounds, including quantitative changes in carbohydrate metabolism and the biosynthesis of organic acids, amino acids, and stress factors [8, 27].

The current work presents GC-MS results from an untargeted metabolomics study of *A. brasilense* culturing. While further validations are needed to confirm the raised hypothesis, important bioactive compounds linked to its interaction with plants were putatively identified. The putative presence of several benzoate-related phenolics is reported (Figs. 2 and 4D, Tables S2 and S3), in agreement with the finding that *A. brasilense* can use benzoate and hydroxybenzoate as carbon and energy sources, and that these compounds enhance the nitrogenase activity [28]. The putative identification of benzoic acid produced by *A. brasilense* in culture media is also consistent with this finding [29]. Interestingly, benzoate, 4-hydroxybenzoate and 3,4-dihydroxybenzoic acid were reported as chemoattractants for *A. brasilense* and may influence the bacterium’s ability to survive and select host plants [30]. In addition, indole-derived benzoxazinoids synthesized by cereals can shape the root microbiome and significantly impact the gene expression, chemotaxis, and ability of *A. brasilense* to colonize the rhizosphere [7].

Several phenolic compounds produced by microorganisms and plants, such as benzoate and benzoic acid, function as important signaling molecules and antimicrobial agents [31, 32]. Maize and sugarcane, for example, increase benzoic acid concentration in their roots under nitrogen scarcity, chemoattracting diazotrophic bacteria while repelling potential pathogens [7, 33]. The results of the current study suggest that mature culture of *A. brasilense* Ab-V5 in the FORM15 produces several benzoate-related phenolics, some of which had not been previously reported for this strain (Table S1). Additionally, maize seeds inoculated with 72-hour Ab-V5 cultures exhibited improved growth-promoting effects compared to seeds inoculated with Ab-V5 cultures for shorter periods, in agreement with previous results [5].

Following FC analysis, eight compounds positively regulated in the 72-hour Ab-V5 cultures were preliminarily assigned to benzoate-derived compounds, including benzoic acid, benzeneacetic acid, and benzeneethanamine. Benzoic acid, a precursor of the phytohormone salicylic acid and siderophores, presents inhibitory activity over the nitrogenase with chemotactic activity, attracting PGPR and repelling phytopathogens [34, 35]. Benzeneacetic acid (phenylacetic acid) has auxin and antimicrobial activity and is synthesized by several plants and microorganisms including *A. brasilense* [36]. Additionally, benzeneethanamine exhibits antibacterial and anti-quorum sensing properties against multidrug-resistant pathogens [37].

Other compounds with increased FC in the 72-hour Ab-V5 metabolome include those preliminarily identified as trehalose, gulose, and glucose- and mannose-derived compounds. Trehalose plays an important role in the physiology of *A. brasilense*, including the formation of biofilms, cell motility, biosynthesis of auxins, tolerance to abiotic and oxidative stresses, and the promotion of maize growth under conditions of water stress [38]. The presence of gulose and glucose-derived forms can result from the partial consumption of sucrose, considering that *A. brasilense* has been reported to metabolize glucose inefficiently, despite harboring the UDP-glucose-4’-epimerase gene [39]. Additionally, the cell membrane and exopolysaccharides produced by *A. brasilense* contain diverse monosaccharides, such as mannose, glucose, arabinose, and galactose, which corroborate its presence in the bacterium metabolome [40].

Glycerol, an ingredient in FORM 15 and found at increased FC, may result from partial consumption of sucrose by Ab-V5, as it can be produced from fructose via the Embden-Meyerhof-Parnas pathway [29, 41]. Compounds putatively assigned as pentanoic acid (valeric acid), the terpenoids 1(2 H)-pentalenone and 3,3a,4,6a-tetrahydro-2 H-naphthalen-2-one, and propanoic acid and its 2-phenylethyl ester derivative, were also at increased FC. Valeric acid, a volatile short-chain fatty acid, has antifungal properties and appears to attract beneficial microorganisms to the rhizosphere of certain plant species [42, 43]. Terpenoids are known to exhibit various biological activities, including antimicrobial, antioxidant and cell signaling properties, which are important in the initial stages of interactions between plants, bacteria, and soil biota [44]. Potential precursors of alkaloid biosynthesis exhibiting antifungal activity were also present at higher FC, including 1-monoisobutyrin bis(trimethylsilyl) and 2-ethyl-2-phenyl-1,3-benzodioxole [45, 46].

Compounds with decreased peak intensity in the inoculated culture medium were preliminarily assigned as follows: D-ribose, mannose, and sucrose derivatives(carbohydrates), tyrosine (amino acid), N-(3-amino-2-hydroxyphenyl)acetamide) (phenolic compound), and diethyl dodecyloxyethoxy (surfactant). The FORM15 medium uses sucrose as carbon and yeast extract as nitrogen and amino acids sources, both at relatively high amounts [5, 47]. The relative lower number of chromatographic peaks of these compounds in the metabolome of 72-hour Ab-V5 suggests its consumption by the bacteria to support cellular processes. In addition, the compound preliminarily identified as diethyldodecyloxyoctyl exhibited lower peak intensity in the 72-hour Ab-V5 metabolome. This finding suggests molecular rearrangement of FORM15 components under the experimental conditions (temperature and agitation) or during sample processing [48].

## Conclusions

Maize inoculated with *A. brasilense* Ab-V5 from FORM15 cultures exhibited variable responses to different incubation times, with increased growth-promotion from cultures with a 72-hour incubation period. Furthermore, the Ab-V5 exometabolome exhibited higher diversity of differentially expressed compounds after a 72-hour incubation period. Several compounds that facilitate efficient interactions between Ab-V5, the host plant, and the soil microbiota were potentially identified. These compounds include carbohydrates, carboxylic acids, phenolic compounds, and several benzoate derivatives. The results presented underscore the importance of expanding our knowledge of the metabolic determinants of PGPB inoculant quality, efficacy, and competitiveness to improve commercial formulation quality. For *A. brasilense* Ab-V5, we hypothesize that these compounds originating from benzoate appear to be of great importance, though this needs to be validated through further studies.

## Supplementary Information

Below is the link to the electronic supplementary material.

Supplementary Material 1 (DOCX 1.12 MB)

## Data Availability

The original contributions presented in the study are included in the article/Supplementary Material, further inquiries can be directed to the corresponding authors.

## Declarations

As this manuscript does not contain any studies involving human participants or animals performed by any of the authors, it is not subject to statements relating to ethics approval, patient consent, permission to reproduce material from other sources, or clinical trial registration.
