# Supplementary material for: Growth promotion of maize and metabolomic profiling of Azospirillum brasilense cultures at varying incubation times
Source: Braz J Microbiol. 2026 Oct 29;57(1):314. doi: 10.1007/s42770-026-02134-0 (PMC13719914; doi:10.1007/s42770-026-02134-0)
Supplement: Supplementary file 1 [file 42770_2026_2134_MOESM1_ESM.docx]

**Figure S1**. Average total ion current (TIC) chromatogram of the untargeted GC-MS metabolome of the FORM15 culture medium inoculated with *A. brasilense* (Azo), compared with uninoculated FORM15 (Cont). A) TIC comparison between inoculated and uninoculated FORM15 after 12 hour incubation period. B) TIC comparison between inoculated and uninoculated FORM15 after 36 hour incubation period. C) TIC comparison between inoculated and uninoculated FORM15 after 72 hour incubation period.

**A)**

**B)**

**C)**

### **Figure S2**. Synchronized 3D plot of the principal component analysis (PCA) representing the variance distribution of the chromatographic peaks recorded from the untargeted GC-MS metabolome of the FORM15 culture medium inoculated with *Azospirillum brasilense* Ab-V5 (72hAzo) and the uninoculated culture medium (72hCont). The PCA was plotted using the three main principal components to better describe the variance distribution of the samples data, collected from the FORM15 supernatant after 72 hours of incubation under orbital shaking at 180 rpm and 28 °C.

**Table S1**. Significant metabolites identified by one-way ANOVA analysis of the peak list obtained from FORM15 culture medium inoculated with *A. brasilense* Azo, compared with uninoculated FORM15 culture medium. This analysis disregarded the incubation time (12, 36 or 72 hours at 180 rpm and 28 °C). Peak ID indicates the number of the peak after XCMS analysis of the chromatograms. Treatment prevalence indicates whether a given peak signal is quantitatively related to samples inoculated with *A. brasilense* Azo (Azo) or the uninoculated FORM15 culture medium (Cont). The *p*-value indicates whether there is a significant difference according to Fisher's test (p < 0.05). The FDR value indicates the false discovery rate attributed to each peak signal. The Match Factor indicates how similar a peak is to the listed compounds in the NIST Mass Spectral Library (excellent match: >900; good match: 800–900; fair match: 700–800; medium to low confidence: <700).

| Peak ID | Treatment prevalence | *p*-value | FDR | *m/z* | RT (s) | Match Factor | Compounds |
| --- | --- | --- | --- | --- | --- | --- | --- |
| FT008 | Azo | 5.40E-03 | 6.90E-02 | 45.99 | 348.0509 | 808 | Glycerol, tris(trimethylsilyl) ether |
| FT010 | Azo | 5.37E-03 | 1.47E-01 | 46.95 | 348.0387 | 950 | Glycerol, tris(trimethylsilyl) ether |
| FT013 | Azo | 5.03E-01 | 2.33E-01 | 46.96 | 359.0704 | 887 | Benzeneacetic acid, trimethylsilyl ester |
| FT021 | Azo | 3.95E-03 | 2.39E-02 | 56.04 | 348.5077 |  | unindentified |
| FT025 | Azo | 2.10E-04 | 2.39E-02 | 57.05 | 404.6882 | 678 | Tyramine, trimethylsilyl ether |
| FT046 | Cont | 4.35E-04 | 3.98E-01 | 65.05 | 359.0704 | 727 | Sym-tetramethyl (diisopropyl)disiloxane |
| FT057 | Azo | 7.41E-01 | 2.56E-04 | 71.05 | 386.9210 | 859 | 1-Mono-isobutyrin, bis(trimethylsilyl)- deriv. |
| FT058 | Azo | 4.00E-03 | 1.29E-03 | 71.06 | 348.5077 |  | unindentified |
| FT059 | Azo | 4.54E-03 | 1.88E-03 | 71.09 | 434.0676 | 682 | 2-Thiobarbituric acid, tris(tert-butyldimethylsilyl) deriv. |
| FT068 | Azo | 1.31E-04 | 2.65E-03 | 73.05 | 358.5492 | 910 | 1(2H)-Pentalenone, 3,3a,4,6a-tetrahydro- |
| FT077 | Azo | 1.33E-03 | 3.30E-03 | 74.05 | 358.6306 | 882 | Benzeneacetic acid, trimethylsilyl ester |
| FT085 | Azo | 1.97E-01 | 3.30E-03 | 75.03 | 358.9076 | 727 | Sym-tetramethyl(diisopropyl)disiloxane |
| FT091 | Azo | 3.89E-03 | 6.82E-03 | 77.04 | 344.0441 | 930 | Benzoic acid trimethylsilyl ester |
| FT094 | Azo | 4.60E-03 | 6.82E-03 | 77.04 | 408.7724 |  | unindentified |
| FT104 | Azo | 2.45E-04 | 6.83E-03 | 85.08 | 404.6882 | 678 | Tyramine, trimethylsilyl ether |
| FT116 | Azo | 1.96E-04 | 6.83E-03 | 88.10 | 486.0066 | 802 | Gulose, 2,3,4,5,6-pentakis-O-(trimethylsilyl)- |
| FT117 | Azo | 3.76E-03 | 6.83E-03 | 88.10 | 426.7490 | 761 | N-(2-Methoxyphenyl)-2-nitrobenzenecarbothioamide |
| FT135 | Azo | 5.82E-04 | 7.04E-03 | 91.10 | 353.8015 | 794 | Benzeneethanamine |
| FT149 | Azo | 3.01E-03 | 7.04E-03 | 99.10 | 422.0801 | 645 | 4-Amino-4,5(1H)-dihydro-1,2,4-triazole-5-one |
| FT154 | Azo | 3.41E-02 | 8.93E-03 | 100.10 | 487.0781 | 837 | Ethyl 2,3,4,6-tetrakis-O-(trimethylsilyl)-D-glucopyranoside |
| FT159 | Azo | 2.54E-04 | 1.07E-02 | 101.09 | 404.8656 | 662 | Bis(tert-butyldimethylsilyl) 3,6,9,12-tetraoxatetradecane-1,14-dioate |
| FT169 | Azo | 5.96E-03 | 1.07E-02 | 103.10 | 442.4285 | 735 | Terephthalic acid, cyclobutyl decyl ester |
| FT174 | Azo | 3.03E-03 | 1.07E-02 | 104.10 | 416.1051 | 646 | Acetamide, N-(2-phenylethyl)- |
| FT177 | Azo | 7.65E-04 | 1.22E-02 | 105.10 | 344.0783 | 867 | Benzoic acid trimethylsilyl ester |
| FT178 | Azo | 9.53E-04 | 1.22E-02 | 105.10 | 428.7658 | 861 | N-(2-Methoxyphenyl)-2-nitrobenzenecarbothioamide |
| FT180 | Azo | 3.21E-01 | 1.55E-02 | 106.09 | 344.5408 | 932 | Benzoic acid trimethylsilyl ester |
| FT203 | Cont | 1.07E-03 | 1.58E-02 | 113.14 | 450.8045 | 449 | Benzene, 1,3-bis(1-allyl-5-tetrazolylthiomethyl)- |
| FT232 | Azo | 1.87E-03 | 1.67E-02 | 120.10 | 483.9204 | 618 | l-Alanine, N-(4-ethylbenzoyl)-, decyl ester |
| FT253 | Azo | 1.53E-03 | 1.73E-02 | 129.10 | 486.0000 | 796 | Ethyl 2,3,4,6-tetrakis-O-(trimethylsilyl)-D-glucopyranoside |
| FT254 | Azo | 1.36E-03 | 1.76E-02 | 129.11 | 429.7755 | 661 | N-(2-Methoxyphenyl)-2-nitrobenzenecarbothioamide # |
| FT277 | Azo | 5.72E-04 | 1.76E-02 | 133.06 | 486.0000 |  | unindentified |
| FT286 | Cont | 3.81E-03 | 1.76E-02 | 135.10 | 344.0949 | 856 | Benzoic acid trimethylsilyl ester |
| FT290 | Azo | 7.52E-01 | 2.10E-02 | 137.08 | 359.1714 | 727 | Sym-tetramethyl(diisopropyl)disiloxane |
| FT305 | Azo | 4.94E-03 | 2.10E-02 | 143.10 | 328.1278 | 603 | Butane, 1,2,3-tris(trimethylsiloxy)- |
| FT321 | Azo | 3.39E-04 | 2.31E-02 | 147.09 | 405.2682 | 678 | Tyramine, trimethylsilyl ether |
| FT328 | Azo | 5.51E-03 | 2.58E-02 | 148.10 | 426.9009 | 766 | Benzenepropanoic acid, α-[(trimethylsilyl)oxy]-, trimethylsilyl ester |
| FT357 | Azo | 2.90E-03 | 2.58E-02 | 159.05 | 348.0262 | 892 | 1-(1-Adamantyl)-2-triisopropylsilyloxyethane |
| FT358 | Azo | 1.81E-03 | 2.58E-02 | 159.09 | 404.7100 | 678 | Tyramine, trimethylsilyl ether |
| FT369 | Azo | 1.79E-04 | 2.65E-02 | 164.10 | 358.1909 | 766 | Sym-tetramethyl (diisopropyl)disiloxane |
| FT373 | Azo | 2.21E-04 | 3.09E-02 | 166.05 | 327.0345 | 799 | Pantoyl lactone, trimethylsilyl |
| FT387 | Cont | 4.34E-03 | 3.10E-02 | 171.10 | 486.0000 | 757 | Glucopyranose, 1,2,3,4,6-pentakis-O-(trimethylsilyl)-, D- |
| FT412 | Azo | 6.08E-03 | 3.45E-02 | 179.05 | 344.0099 | 776 | Benzeneethanamine |
| FT418 | Azo | 4.64E-04 | 3.54E-02 | 180.06 | 344.0100 | 729 | Pyridine, 6-methyl-2-[(2,3-dimethyl-2-butyl)dimethylsilyloxy]- |
| FT423 | Cont | 1.32E-03 | 3.54E-02 | 181.05 | 352.0240 | 691 | Thiophene, 2,5-bis(1,1-dimethylethyl)- |
| FT453 | Azo | 5.61E-04 | 3.54E-02 | 193.08 | 359.0704 | 887 | Benzeneacetic acid, trimethylsilyl ester |
| FT479 | Azo | 8.13E-04 | 3.72E-02 | 203.09 | 432.5015 | 749 | Furan, 2,5-dihydro-2,5-dimethoxy- |
| FT480 | Azo | 8.92E-04 | 4.05E-02 | 203.09 | 404.7100 | 678 | Tyramine, trimethylsilyl ether |
| FT481 | Azo | 2.27E-03 | 4.05E-02 | 203.10 | 387.3405 | 893 | 1-Mono-isobutyrin, bis(trimethylsilyl)- deriv. |
| FT484 | Azo | 4.33E-03 | 4.05E-02 | 204.09 | 509.0190 | 525 | Phenylthioacetic acid, 4-cyanophenyl ester |
| FT487 | Azo | 4.14E-03 | 4.05E-02 | 204.11 | 508.7986 | 751 | Gulose, 2,3,4,5,6-pentakis-O-(trimethylsilyl)- |
| FT522 | Azo | 2.42E-03 | 4.05E-02 | 217.10 | 508.9257 | 601 | β-Gentiobiose, octakis(trimethylsilyl) ether |
| FT528 | Azo | 2.76E-03 | 4.05E-02 | 218.10 | 486.0000 |  | unindentified |
| FT529 | Azo | 5.13E-03 | 4.11E-02 | 218.09 | 441.9106 | 894 | α-D-Glucopyranoside, 1,3,4,6-tetrakis-O-(trimethylsilyl)-β-D-fructofuranosyl 2,3,4,6-tetrakis-O-(trimethylsilyl)- |
| FT530 | Azo | 1.81E-04 | 4.15E-02 | 218.09 | 456.8384 | 755 | D-(-)-Ribofuranose, tetrakis (trimethylsilyl) ether (isomer 2) |
| FT559 | Azo | 3.62E-03 | 4.15E-02 | 229.09 | 486.0000 |  | unindentified |
| FT578 | Azo | 4.69E-04 | 4.32E-02 | 233.11 | 486.0000 |  | unindentified |
| FT596 | Azo | 4.80E-03 | 4.43E-02 | 243.10 | 441.9198 | 707 | Sucrose, octakis(trimethylsilyl) ether |
| FT610 | Azo | 5.72E-03 | 4.48E-02 | 250.10 | 433.8238 | 510 | Chromic acetylacetonate |
| FT611 | Azo | 1.88E-04 | 4.57E-02 | 251.09 | 482.0141 | 741 | β-D-Mannopyranoside, methyl 2,3,4,6-tetrakis-O-(trimethylsilyl)- |
| FT613 | Azo | 1.96E-03 | 4.66E-02 | 257.09 | 441.9198 | 907 | Sucrose, octakis(trimethylsilyl) ether |
| FT637 | Azo | 2.35E-03 | 4.66E-02 | 271.09 | 485.7143 | 804 | Ethyl 2,3,4,6-tetrakis-O-(trimethylsilyl)-D-glucopyranoside |
| FT668 | Azo | 3.26E-03 | 4.68E-02 | 291.10 | 486.0000 |  | unindentified |
| FT712 | Azo | 1.05E-03 | 4.77E-02 | 331.14 | 485.8571 | 810 | Mannose, 6-deoxy-2,3,4,5-tetrakis-O-(trimethylsilyl)-, L- |
| FT740 | Azo | 1.86E-03 | 4.90E-02 | 362.13 | 485.3125 |  | unindentified |
| FT769 | Azo | 1.04E-03 | 4.92E-02 | 438.21 | 441.3501 | 862 | D-(+)-Trehalose, octakis (trimethylsilyl) ether |

**Table S2**. Top 100 metabolites used to build the heatmap shown in Figure 3C, selected according to their significant variation in peak intensity as determined by one-way ANOVA analysis (T-test). The peak list includes metabolites from both the inoculated and uninoculated *A. brasilense* Ab-V5 FORM15 culture medium, regardless the incubation time (12, 36 or 72 hours at 180 rpm and 28 °C). Peak ID indicates the number of the peak after XCMS analysis of the chromatograms, and the peaks are presented in the same order as in Fig. 3C (from top to bottom). The *p*-value indicates whether there is a significant difference according to Fisher's test (p < 0.05). The Match Factor indicates how similar a peak is to the listed compounds in the NIST Mass Spectral Library (excellent match: >900; good match: 800–900; fair match: 700–800; medium to low confidence: <700).

| Peak ID | *p*-value | *m/z* | RT (s) | Match Factor | Compounds |
| --- | --- | --- | --- | --- | --- |
| FT094 | 4.60E-03 | 77.04 | 408.7724 |  | unindentified |
| FT373 | 2.21E-04 | 166.05 | 327.0345 | 799 | Pantoyl lactone, trimethylsilyl |
| FT740 | 1.86E-03 | 362.13 | 485.3125 |  | unindentified |
| FT712 | 1.05E-03 | 331.14 | 485.8571 | 810 | Mannose, 6-deoxy-2,3,4,5-tetrakis-O-(trimethylsilyl)-, L- |
| FT203 | 1.07E-03 | 113.14 | 450.8045 | 449 | Benzene, 1,3-bis(1-allyl-5-tetrazolylthiomethyl)- |
| FT246 | 2.22E-03 | 127.13 | 467.1968 | 465 | Benzene, 2-(1-decylundecyl)-1,4-dimethyl- |
| FT520 | 2.19E-02 | 217.10 | 457.2807 | 720 | Silane, diethylethoxyoctyloxy- |
| FT530 | 1.81E-04 | 218.09 | 456.8384 | 755 | D-(-)-Ribofuranose, tetrakis (trimethylsilyl) ether (isomer 2) |
| FT484 | 4.33E-03 | 204.09 | 509.0190 | 525 | Phenylthioacetic acid, 4-cyanophenyl ester |
| FT487 | 4.14E-03 | 204.11 | 508.7986 | 751 | Gulose, 2,3,4,5,6-pentakis-O-(trimethylsilyl)- |
| FT522 | 2.42E-03 | 217.10 | 508.9257 | 601 | β-Gentiobiose, octakis(trimethylsilyl) ether |
| FT399 | 3.22E-02 | 175.10 | 509.0191 | 806 | Mannose, 6-deoxy-2,3,4,5-tetrakis-O-(trimethylsilyl)-, L- |
| FT442 | 5.28E-03 | 191.05 | 508.1164 |  | unindentified |
| FT149 | 3.01E-03 | 99.10 | 422.0801 | 645 | 4-Amino-4,5(1H)-dihydro-1,2,4-triazole-5-one |
| FT571 | 3.86E-02 | 231.15 | 433.9338 | 685 | Benzenamine, N,N'-(1,2-dimethyl-1,2-ethanediylidene)bis[2,6-bis(1-methylethyl)- |
| FT059 | 4.54E-03 | 71.09 | 434.0676 | 682 | 2-Thiobarbituric acid, tris(tert-butyldimethylsilyl) deriv. |
| FT479 | 8.13E-04 | 203.09 | 432.5015 | 749 | Furan, 2,5-dihydro-2,5-dimethoxy- |
| FT422 | 2.91E-02 | 180.11 | 409.6467 | 701 | Tyramine, trimethylsilyl ether |
| FT277 | 5.72E-04 | 133.06 | 486.0000 |  | unindentified |
| FT528 | 2.76E-03 | 218.10 | 486.0000 |  | unindentified |
| FT668 | 3.26E-03 | 291.10 | 486.0000 |  | unindentified |
| FT734 | 1.36E-02 | 361.11 | 485.3125 | 564 | D-(+)-Trehalose, octakis(trimethylsilyl) ether |
| FT578 | 4.69E-04 | 233.11 | 486.0000 |  | unindentified |
| FT088 | 3.91E-02 | 76.02 | 486.0000 |  | unindentified |
| FT660 | 3.13E-02 | 289.10 | 486.0000 | 832 | Ethyl 2,3,4,6-tetrakis-O-(trimethylsilyl)-D-glucopyranoside |
| FT250 | 3.94E-02 | 129.10 | 348.0504 | 750 | Glycerol, tris(trimethylsilyl) ether |
| FT488 | 3.84E-02 | 204.13 | 348.3706 | 757 | Glycerol, tris(trimethylsilyl) ether |
| FT483 | 3.16E-02 | 203.10 | 348.1618 | 757 | Glycerol, tris(trimethylsilyl) ether |
| FT036 | 3.71E-02 | 60.00 | 348.0504 | 750 | Glycerol, tris(trimethylsilyl) ether |
| FT486 | 2.67E-02 | 204.10 | 348.0387 | 774 | Silane, trimethyl (propoxymethyl)- |
| FT042 | 2.46E-02 | 62.98 | 300.1271 | 931 | Benzeneethanamine |
| FT764 | 3.03E-03 | 437.15 | 442.2019 | 764 | Sucrose, octakis(trimethylsilyl) ether |
| FT658 | 2.88E-02 | 289.10 | 447.0771 | 648 | Ribitol, 1,2,3,4,5-pentakis-O-(trimethylsilyl)- |
| FT182 | 2.34E-02 | 107.10 | 495.6162 | 766 | Tyrosine |
| FT750 | 8.64E-03 | 365.15 | 446.9551 | 648 | Ribitol, 1,2,3,4,5-pentakis-O-(trimethylsilyl)- |
| FT383 | 3.54E-02 | 170.10 | 478.0498 | 682 | Glycyl-L-tyrosine |
| FT713 | 4.61E-03 | 331.11 | 441.9236 | 929 | α-D-Glucopyranoside, 1,3,4,6-tetrakis-O-(trimethylsilyl)-β-D-fructofuranosyl 2,3,4,6-tetrakis-O-(trimethylsilyl)- |
| FT260 | 2.74E-02 | 130.09 | 425.8345 | 828 | Benzenepropanoic acid, α-[(trimethylsilyl)oxy]-, trimethylsilyl ester |
| FT135 | 5.82E-04 | 91.10 | 353.8015 | 794 | Benzeneethanamine |
| FT139 | 1.20E-02 | 92.09 | 354.7446 | 748 | Benzeneethanamine |
| FT259 | 2.75E-02 | 130.10 | 469.3360 | 703 | Alanine, N-methyl-N-ethoxycarbonyl-, dodecyl ester |
| FT186 | 1.32E-02 | 107.10 | 478.9640 | 682 | Glycyl-L-tyrosine |
| FT771 | 2.39E-02 | 439.20 | 441.3501 | 707 | Sucrose, octakis(trimethylsilyl) ether |
| FT232 | 1.87E-03 | 120.10 | 483.9204 | 618 | l-Alanine, N-(4-ethylbenzoyl)-, decyl ester |
| FT564 | 3.85E-02 | 230.10 | 432.6191 | 701 | Glucuronolactone, trisO-(trimethylsilyl)- |
| FT610 | 5.72E-03 | 250.10 | 433.8238 | 510 | Chromic acetylacetonate |
| FT529 | 1.28E-02 | 218.09 | 441.9106 | 894 | α-D-Glucopyranoside, 1,3,4,6-tetrakis-O-(trimethylsilyl)-β-D-fructofuranosyl 2,3,4,6-tetrakis-O-(trimethylsilyl)- |
| FT613 | 1.96E-03 | 257.09 | 441.9198 | 907 | Sucrose, octakis(trimethylsilyl) ether |
| FT320 | 3.49E-02 | 147.09 | 441.9144 | 735 | Terephthalic acid, cyclobutyl decyl ester |
| FT382 | 2.24E-02 | 169.10 | 441.9044 |  | unindentified |
| FT169 | 5.96E-03 | 103.10 | 442.4285 | 735 | Terephthalic acid, cyclobutyl decyl ester |
| FT741 | 1.99E-02 | 362.12 | 442.2020 | 829 | α-D-Glucopyranoside, 1,3,4,6-tetrakis-O-(trimethylsilyl)-β-D-fructofuranosyl 2,3,4,6-tetrakis-O-(trimethylsilyl)- |
| FT638 | 3.76E-02 | 271.10 | 441.9160 | 773 | Sucrose, octakis(trimethylsilyl) ether |
| FT596 | 4.80E-03 | 243.10 | 441.9198 | 707 | Sucrose, octakis(trimethylsilyl) ether |
| FT601 | 2.37E-02 | 244.10 | 441.9053 |  | unindentified |
| FT441 | 3.12E-02 | 191.04 | 442.0627 |  | unindentified |
| FT702 | 1.22E-02 | 319.10 | 442.0628 | 829 | α-D-Glucopyranoside, 1,3,4,6-tetrakis-O-(trimethylsilyl)-β-D-fructofuranosyl 2,3,4,6-tetrakis-O-(trimethylsilyl)- |
| FT704 | 3.63E-02 | 319.16 | 441.3573 | 907 | Sucrose, octakis(trimethylsilyl) ether |
| FT749 | 6.08E-03 | 364.17 | 441.1653 |  | unindentified |
| FT412 | 2.18E-02 | 179.05 | 344.0099 | 776 | Benzeneethanamine |
| FT003 | 9.78E-03 | 44.95 | 359.2051 | 702 | Sym-tetramethyl (diisopropyl)disiloxane |
| FT091 | 3.89E-03 | 77.04 | 344.0441 | 930 | Benzoic acid trimethylsilyl ester |
| FT418 | 4.64E-04 | 180.06 | 344.0100 | 729 | Pyridine, 6-methyl-2-[(2,3-dimethyl-2-butyl)dimethylsilyloxy]- |
| FT286 | 3.81E-03 | 135.10 | 344.0949 | 856 | Benzoic acid trimethylsilyl ester |
| FT177 | 7.65E-04 | 105.10 | 344.0783 | 867 | Benzoic acid trimethylsilyl ester |
| FT369 | 1.79E-04 | 164.10 | 358.1909 | 766 | Sym-tetramethyl (diisopropyl)disiloxane |
| FT085 | 1.97E-01 | 75.03 | 358.9076 | 727 | Sym-tetramethyl(diisopropyl)disiloxane |
| FT648 | 3.32E-02 | 281.00 | 552.0428 | 361 | Pyrrolo[1,2-a]pyrazine-1,4-dione, hexahydro-3-(2-methylpropyl)- |
| FT159 | 2.54E-04 | 101.09 | 404.8656 | 662 | Bis(tert-butyldimethylsilyl) 3,6,9,12-tetraoxatetradecane-1,14-dioate |
| FT611 | 1.88E-04 | 251.09 | 482.0141 | 741 | β-D-Mannopyranoside, methyl 2,3,4,6-tetrakis-O-(trimethylsilyl)- |
| FT358 | 1.81E-03 | 159.09 | 404.4064 | 678 | Tyramine, trimethylsilyl ether |
| FT321 | 3.39E-04 | 147.09 | 405.2682 | 678 | Tyramine, trimethylsilyl ether |
| FT284 | 2.20E-02 | 134.10 | 388.0871 | 724 | Silane, trimethyl (tricyclo[3.3.1.1(3,7)]dec-2-yloxy)- |
| FT104 | 2.45E-04 | 85.08 | 404.6882 | 678 | Tyramine, trimethylsilyl ether |
| FT025 | 2.10E-04 | 57.05 | 404.6882 | 678 | Tyramine, trimethylsilyl ether |
| FT480 | 8.92E-04 | 203.09 | 404.7100 | 678 | Tyramine, trimethylsilyl ether |
| FT134 | 2.74E-02 | 91.10 | 358.6306 | 787 | Benzeneacetic acid, trimethylsilyl ester |
| FT077 | 1.33E-03 | 74.05 | 358.6306 | 882 | Benzeneacetic acid, trimethylsilyl ester |
| FT068 | 1.31E-04 | 73.05 | 358.5492 | 910 | 1(2H)-Pentalenone, 3,3a,4,6a-tetrahydro- |
| FT013 | 5.03E-01 | 46.96 | 359.0704 | 887 | Benzeneacetic acid, trimethylsilyl ester |
| FT481 | 2.27E-03 | 203.10 | 387.3405 | 893 | 1-Mono-isobutyrin, bis(trimethylsilyl)- deriv. |
| FT453 | 5.61E-04 | 193.08 | 359.0704 | 887 | Benzeneacetic acid, trimethylsilyl ester |
| FT057 | 7.41E-01 | 71.05 | 386.9210 | 859 | 1-Mono-isobutyrin, bis(trimethylsilyl)- deriv. |
| FT290 | 7.52E-01 | 137.08 | 359.1714 | 727 | Sym-tetramethyl(diisopropyl)disiloxane |
| FT046 | 4.35E-04 | 65.05 | 359.0704 | 727 | Sym-tetramethyl (diisopropyl)disiloxane |
| FT180 | 3.21E-01 | 106.09 | 344.5408 | 932 | Benzoic acid trimethylsilyl ester |
| FT106 | 2.45E-02 | 85.05 | 390.3981 | 607 | Pentanethioic acid, S-ethyl ester |
| FT154 | 3.41E-02 | 100.10 | 487.0781 | 837 | Ethyl 2,3,4,6-tetrakis-O-(trimethylsilyl)-D-glucopyranoside |
| FT029 | 3.80E-02 | 58.04 | 404.7878 | 662 | Bis(tert-butyldimethylsilyl) 3,6,9,12-tetraoxatetradecane-1,14-dioate |
| FT254 | 1.36E-03 | 129.11 | 429.7755 | 661 | N-(2-Methoxyphenyl)-2-nitrobenzenecarbothioamide # |
| FT116 | 1.96E-04 | 88.10 | 486.0066 | 802 | Gulose, 2,3,4,5,6-pentakis-O-(trimethylsilyl)- |
| FT253 | 1.53E-03 | 129.10 | 486.0000 | 796 | Ethyl 2,3,4,6-tetrakis-O-(trimethylsilyl)-D-glucopyranoside |
| FT333 | 1.10E-02 | 149.10 | 332.0562 | 716 | 2-Ethyl-2-phenyl-1,3-benzodioxole |
| FT769 | 1.04E-03 | 438.21 | 441.3501 | 862 | D-(+)-Trehalose, octakis (trimethylsilyl) ether |
| FT187 | 2.65E-02 | 107.10 | 486.4315 | 630 | Ethyl 2,3,4,6-tetrakis-O-(trimethylsilyl)-D-glucopyranoside |
| FT233 | 4.02E-03 | 121.10 | 486.3158 | 630 | Ethyl 2,3,4,6-tetrakis-O-(trimethylsilyl)-D-glucopyranoside |
| FT117 | 3.76E-03 | 88.10 | 426.7490 | 761 | N-(2-Methoxyphenyl)-2-nitrobenzenecarbothioamide |
| FT178 | 9.53E-04 | 105.10 | 428.7658 | 861 | N-(2-Methoxyphenyl)-2-nitrobenzenecarbothioamide |
| FT022 | 1.52E-02 | 56.04 | 486.5014 | 576 | Methylphosphonic acid, fluoroanhydride, 3-methyl-1-(2-methylpropyl)butyl ester |
| FT328 | 5.51E-03 | 148.10 | 426.9009 | 766 | Benzenepropanoic acid, α-[(trimethylsilyl)oxy]-, trimethylsilyl ester |

**Table S3**. Differential metabolites in the *A. brasilense* Ab-V5 culture at FORM15, compared to the uninoculated medium after an incubation period of 72 h at 180 rpm and 28 °C. Peak ID indicates the peak number after XCMS analysis of the chromatograms. VIP score denotes the value of variable importance in projection, considering the component 1 of multivariate supervised discriminant analysis by partial least squares (sPLS-DA). The *p*-value indicates whether a significant difference (p < 0.05) or a non-significant difference (NS) is present according to Student's t-test. FC stands for fold-change analysis, which is the ratio between group means using data prior to column-wise normalization. Log2(FC) indicates the log2 transformed value for each comparison. Match Factor indicates the similarity score of a peak to the listed compounds in the NIST Mass Spectral Library (excellent match: >900; good match: 800–900; fair match: 700–800; medium to low confidence: <700).

| Peak ID | VIP score | *p*-value | FC | log2(FC) | *m/z* | RT (s) | Match Factor | Compound |
| --- | --- | --- | --- | --- | --- | --- | --- | --- |
| FT003 | 1.6743 | 3.13E-02 | 5.8096 | 2.5384 | 44.95 | 359.2051 | 702 | Sym-tetramethyl (diisopropyl)disiloxane |
| FT013 | 1.9098 | 1.20E-03 | 2.9633 | 1.5672 | 46.96 | 359.0704 | 887 | Benzeneacetic acid, trimethylsilyl ester |
| FT025 | 1.9579 | 2.36E-05 | 11.097 | 3.472 | 57.05 | 404.6882 | 678 | Tyramine, trimethylsilyl ether |
| FT026 | 1.5251 | NS | 4.093 | 20.331 | 57.05 | 390.3981 | 513 | 1H-Tetrazol-5-amine |
| FT029 | 1.4346 | NS | 3.7864 | 1.9208 | 58.04 | 404.7878 | 662 | Bis(tert-butyldimethylsilyl) 3,6,9,12-tetraoxatetradecane-1,14-dioate |
| FT043 | 0.8879 | NS | 2.7511 | 1.460 | 63.00 | 358.6306 |  | unindentified |
| FT046 | 1.9303 | 4.87E-04 | 4.6467 | 2.2162 | 65.05 | 359.0704 | 727 | Sym-tetramethyl (diisopropyl)disiloxane |
| FT057 | 1.8945 | 1.95E-03 | 9.7353 | 3.2832 | 71.05 | 386.921 | 859 | 1-Mono-isobutyrin, bis(trimethylsilyl)- deriv. |
| FT060 | 1.7137 | 2.36E-02 | 0.255 | -1.9712 | 72.05 | 478.3067 | 669 | 2,2-Dipropyl-N-ethylpiperidine |
| FT068 | 1.8955 | 1.90E-03 | 9.4653 | 3.2426 | 73.05 | 358.5492 | 910 | 1(2H)-Pentalenone, 3,3a,4,6a-tetrahydro- |
| FT077 | 1.8117 | 8.98E-03 | 6.3195 | 2.6598 | 74.05 | 358.6306 | 882 | Benzeneacetic acid, trimethylsilyl ester |
| FT085 | 1.9175 | 9.00E-04 | 7.1706 | 2.8421 | 75.03 | 358.9076 | 727 | Sym-tetramethyl(diisopropyl)disiloxane |
| FT091 | 1.8798 | 2.83E-03 | 3.3105 | 1.727 | 77.04 | 344.0441 | 930 | Benzoic acid trimethylsilyl ester |
| FT104 | 1.9653 | 1.05E-07 | 23.782 | 4.5718 | 85.08 | 404.6882 | 678 | Tyramine, trimethylsilyl ether |
| FT106 | 1.5511 | NS | 5.731 | 2.5188 | 85.05 | 390.3981 | 607 | Pentanethioic acid, S-ethyl ester |
| FT116 | 1.8971 | 1.81E-03 | 9.3003 | 3.2173 | 88.10 | 486.0066 | 802 | Gulose, 2,3,4,5,6-pentakis-O-(trimethylsilyl)- |
| FT126 | 0.6501 | NS | 3.3665 | 1.7512 | 90.06 | 357.9671 | 721 | propanoic acid, 2-oxo-, trimethylsilyl ester |
| FT134 | 1.9046 | 1.44E-03 | 7.814 | 2.9661 | 91.10 | 358.6306 | 787 | Benzeneacetic acid, trimethylsilyl ester |
| FT147 | 0.8180 | NS | 2.7541 | 1.4616 | 99.05 | 348.038 |  | unindentified |
| FT148 | 1.4375 | NS | 0.323 | -1.6287 | 99.10 | 433.8238 | 758 | Furan, 2,5-dihydro-2,5-dimethoxy- |
| FT149 | 1.8759 | 3.09E-03 | 0.127 | -2.9779 | 99.10 | 422.0801 | 645 | 4-Amino-4,5(1H)-dihydro-1,2,4-triazole-5-one |
| FT154 | 1.8893 | 2.24E-03 | 73.771 | 6.205 | 100.10 | 487.0781 | 837 | Ethyl 2,3,4,6-tetrakis-O-(trimethylsilyl)-D-glucopyranoside |
| FT159 | 1.9622 | 5.04E-06 | 6.0644 | 2.6004 | 101.09 | 404.8656 | 662 | Bis(tert-butyldimethylsilyl) 3,6,9,12-tetraoxatetradecane-1,14-dioate |
| FT174 | 1.6765 | 3.09E-02 | 2.1288 | 1.090 | 104.10 | 416.1051 | 646 | Acetamide, N-(2-phenylethyl)- |
| FT177 | 1.8867 | 2.40E-03 | 8.8891 | 3.152 | 105.10 | 344.0783 | 867 | Benzoic acid trimethylsilyl ester |
| FT180 | 1.9235 | 6.89E-04 | 3.4926 | 1.8043 | 106.09 | 344.5408 | 932 | Benzoic acid trimethylsilyl ester |
| FT182 | 1.8576 | 4.47E-03 | 0.278 | -1.8453 | 107.10 | 495.9923 | 766 | Tyrosine |
| FT203 | 1.5624 | NS | 0.366 | -1.4521 | 113.14 | 450.8045 | 449 | Benzene, 1,3-bis(1-allyl-5-tetrazolylthiomethyl)- |
| FT206 | 0.9301 | NS | 2.1605 | 1.1114 | 114.10 | 483.9204 | 618 | l-Alanine, N-(4-ethylbenzoyl)-, decyl ester |
| FT232 | 1.5409 | NS | 2.6465 | 1.4041 | 120.10 | 483.9204 | 618 | l-Alanine, N-(4-ethylbenzoyl)-, decyl ester |
| FT246 | 1.7063 | 2.50E-02 | 0.394 | -1.3456 | 127.13 | 467.1968 | 465 | Benzene, 2-(1-decylundecyl)-1,4-dimethyl- |
| FT253 | 1.4875 | NS | 3.1621 | 1.6609 | 129.10 | 486.0000 | 796 | Ethyl 2,3,4,6-tetrakis-O-(trimethylsilyl)-D-glucopyranoside |
| FT254 | 1.6584 | 3.48E-02 | 11.785 | 3.5588 | 129.11 | 429.7755 | 661 | N-(2-Methoxyphenyl)-2-nitrobenzenecarbothioamide # |
| FT258 | 1.4987 | NS | 3.6042 | 1.8497 | 130.09 | 347.9841 | 948 | Glycerol, tris(trimethylsilyl) ether |
| FT269 | 1.7234 | 2.19-E02 | 2.8077 | 1.4894 | 132.06 | 348.0265 |  | unindentified |
| FT284 | 1.9135 | 1.05E-03 | 7.2377 | 2.8555 | 134.10 | 388.0871 | 724 | Silane, trimethyl (tricyclo[3.3.1.1(3,7)]dec-2-yloxy)- |
| FT286 | 1.9010 | 1.61E-03 | 6.4798 | 2.696 | 135.10 | 344.0949 | 856 | Benzoic acid trimethylsilyl ester |
| FT290 | 1.8993 | 1.70E-03 | 5.8662 | 2.5524 | 137.08 | 359.1714 | 727 | Sym-tetramethyl(diisopropyl)disiloxane |
| FT311 | 0.3054 | NS | 2.5054 | 1.325 | 145.08 | 492.1170 | 549 | Trans-3-methylpent-3-ene-1-yn-5-ol, acetate |
| FT321 | 1.9643 | 8.85E-07 | 12.184 | 3.6069 | 147.09 | 405.2682 | 678 | Tyramine, trimethylsilyl ether |
| FT333 | 1.6909 | 2.80E-02 | 40.219 | 5.3298 | 149.10 | 332.0562 | 716 | 2-Ethyl-2-phenyl-1,3-benzodioxole |
| FT346 | 1.5147 | NS | 2.083 | 1.0587 | 154.09 | 483.2593 | 682 | 7-Isoquinolinol, 1,2,3,4-tetrahydro-6-methoxy-2-methyl-1-[2-(3,4,5-trimethoxyphenyl)ethyl]- |
| FT352 | 1.6742 | 3.14E-02 | 0.456 | -1.1331 | 156.08 | 453.1774 | 412 | Benzenemethanol, α-(chloromethyl)- |
| FT358 | 1.9447 | 1.73E-04 | 9.8137 | 3.2948 | 159.09 | 404.7100 | 678 | Tyramine, trimethylsilyl ether |
| FT369 | 1.8777 | 2.97E-03 | 11.556 | 3.5305 | 164.10 | 358.1909 | 766 | Sym-tetramethyl (diisopropyl)disiloxane |
| FT372 | 0.3734 | NS | 4.2399 | 2.084 | 165.10 | 358.0790 | 760 | Benzeneacetic acid, trimethylsilyl ester |
| FT393 | 0.9767 | NS | 2.8483 | 1.5101 | 174.07 | 347.9530 | 802 | Glycerol, tris(trimethylsilyl) ether |
| FT399 | 1.4731 | NS | 0.358 | -1.4824 | 175.10 | 509.0191 | 806 | Mannose, 6-deoxy-2,3,4,5-tetrakis-O-(trimethylsilyl)-, L- |
| FT412 | 1.7759 | 1.36E-02 | 7.5912 | 2.9243 | 179.05 | 344.0099 | 776 | Benzeneethanamine |
| FT418 | 1.8542 | 4.75E-03 | 3.3546 | 1.7462 | 180.06 | 344.0100 | 729 | Pyridine, 6-methyl-2-[(2,3-dimethyl-2-butyl)dimethylsilyloxy]- |
| FT453 | 1.9307 | 4.75E-04 | 7.6923 | 2.9434 | 193.08 | 359.0704 | 887 | Benzeneacetic acid, trimethylsilyl ester |
| FT455 | 1.7813 | 1.28E-02 | 4.6058 | 2.2035 | 193.10 | 424.6939 | 766 | Benzenepropanoic acid, α-[(trimethylsilyl)oxy]-, trimethylsilyl ester |
| FT456 | 0.9939 | NS | 3.3144 | 1.7288 | 194.06 | 344.0441 |  | unindentified |
| FT457 | 0.8227 | NS | 2.6288 | 1.3944 | 194.05 | 358.6643 |  | unindentified |
| FT461 | 0.1701 | NS | 18.320 | 4.1954 | 194.09 | 372.3615 | 645 | Sym-tetramethyl(diisopropyl)disiloxane |
| FT463 | 0.5022 | NS | 13.226 | 3.7253 | 195.10 | 375.2696 | 464 | d,l-trans-4-Methyl-5-methoxy-1-(1-methoxy-1-isopropyl)cyclohex-3-ene |
| FT465 | 0.2036 | NS | 3.3628 | 1.7496 | 196.11 | 376.3305 |  | unindentified |
| FT466 | 1.4199 | NS | 2.882 | 1.5271 | 199.04 | 378.8215 |  | unindentified |
| FT472 | 0.5805 | NS | 2.2962 | 1.1992 | 200.11 | 483.9204 | 618 | l-Alanine, N-(4-ethylbenzoyl)-, decyl ester |
| FT480 | 1.9283 | 5.44E-04 | 9.5338 | 3.2531 | 203.09 | 404.7100 | 678 | Tyramine, trimethylsilyl ether |
| FT481 | 1.9202 | 8.02E-04 | 14.386 | 3.8466 | 203.10 | 387.3405 | 893 | 1-Mono-isobutyrin, bis(trimethylsilyl)- deriv. |
| FT484 | 1.4726 | NS | 0.075 | -3.7338 | 204.09 | 509.0190 | 525 | Phenylthioacetic acid, 4-cyanophenyl ester |
| FT486 | 1.4674 | NS | 2.9362 | 1.554 | 204.10 | 348.0387 | 774 | Silane, trimethyl (propoxymethyl)- |
| FT487 | 1.1718 | NS | 0.092 | -3.4398 | 204.11 | 508.7986 | 751 | Gulose, 2,3,4,5,6-pentakis-O-(trimethylsilyl)- |
| FT493 | 1.5919 | NS | 2.9155 | 1.5437 | 206.10 | 348.1303 | 551 | 2-Octen-1-ol |
| FT507 | 0.6927 | NS | 4.5895 | 2.1983 | 210.11 | 376.8694 |  | unindentified |
| FT513 | 0.7597 | NS | 3.523 | 1.8168 | 214.15 | 485.6559 |  | unindentified |
| FT520 | 1.5700 | NS | 0.274 | -1.8662 | 217.10 | 457.2807 | 720 | Silane, diethylethoxyoctyloxy- |
| FT522 | 1.5400 | NS | 0.238 | -2.0716 | 217.10 | 508.9257 | 601 | β-Gentiobiose, octakis(trimethylsilyl) ether |
| FT524 | 1.4732 | NS | 0.232 | -2.1088 | 217.09 | 418.0645 | 788 | Sucrose, octakis (trimethylsilyl) ether |
| FT525 | 1.1297 | NS | 3.8614 | 1.9491 | 217.10 | 491.1234 | 690 | p-Hexyloxyphenyl trans-4-butylcyclohexanecarboxylate |
| FT530 | 1.6931 | 2.75E-02 | 0.299 | -1.7403 | 218.09 | 456.8384 | 755 | D-(-)-Ribofuranose, tetrakis (trimethylsilyl) ether (isomer 2) |
| FT531 | 1.5888 | NS | 2.8839 | 1.528 | 218.10 | 348.0756 |  | unindentified |
| FT532 | 1.5019 | NS | 11.679 | 3.5459 | 218.15 | 404.7660 | 703 | 1-Cyclohexyldimethylsilyloxy-2-phenylethane |
| FT535 | 0.2066 | NS | 6.4384 | 2.6867 | 219.09 | 404.8656 | 665 | Bis(tert-butyldimethylsilyl) 3,6,9,12-tetraoxatetradecane-1,14-dioate |
| FT537 | 1.1487 | NS | 0.3168 | -1.6583 | 219.10 | 456.8152 |  | unindentified |
| FT549 | 1.2357 | NS | 6.7036 | 2.7449 | 223.11 | 447.2252 | 695 | 2,4(3H,5H)-Furandione, 3-methyl- |
| FT551 | 0.9377 | NS | 5.0902 | 2.3477 | 223.10 | 427.1036 | 629 | Ethaneperoxoic acid, 1-cyano-1-phenylbutyl ester |
| FT552 | 0.2002 | NS | 4.1491 | 2.0528 | 223.13 | 486.0000 |  | unindentified |
| FT553 | 1.0843 | NS | 3.417 | 1.7727 | 224.12 | 388.3017 | 732 | Propanoic acid, 2-phenylethyl ester |
| FT584 | 1.2550 | NS | 4.954 | 2.3086 | 235.11 | 484.7440 | 751 | Gulose, 2,3,4,5,6-pentakis-O-(trimethylsilyl)- |
| FT585 | 0.8069 | NS | 2.0874 | 1.0617 | 236.11 | 483.7046 |  | unindentified |
| FT593 | 0.5754 | NS | 0.480 | -1.060 | 242.12 | 486.0000 |  | unindentified |
| FT598 | 1.4556 | NS | 0.274 | -1.8693 | 244.10 | 342.9062 | 877 | N-(3-Amino-2-hydroxy-phenyl)-acetamide |
| FT609 | 0.8484 | NS | 0.317 | -1.658 | 247.10 | 485.9937 |  | unindentified |
| FT611 | 1.9047 | 1.43E-03 | 10.425 | 3.3819 | 251.09 | 482.0141 | 741 | β-D-Mannopyranoside, methyl 2,3,4,6-tetrakis-O-(trimethylsilyl)- |
| FT612 | 0.4487 | NS | 2.5602 | 1.3563 | 252.09 | 484.6125 | 597 | 5,13-Eicosadienoic acid, DMOX derivative |
| FT618 | 0.1768 | NS | 0.343 | -1.5458 | 259.16 | 466.8774 |  | unindentified |
| FT658 | 1.5199 | NS | 0.123 | -3.0294 | 289.10 | 447.0771 | 648 | Ribitol, 1,2,3,4,5-pentakis-O-(trimethylsilyl)- |
| FT659 | 0.9858 | NS | 0.303 | -1.7238 | 289.09 | 433.5910 | 472 | Thymol-β-d-glucopyranoside, tetrakis(O-trimethylsilyl)- |
| FT662 | 1.5088 | NS | 0.154 | -2.6984 | 289.13 | 445.8668 | 634 | D-(+)-Trehalose, octakis(trimethylsilyl) ether |
| FT672 | 1.2356 | NS | 0.351 | -1.5114 | 293.09 | 485.7125 |  | unindentified |
| FT713 | 1.7296 | 2.08E-02 | 0.152 | -2.7138 | 331.11 | 441.9236 | 929 | α-D-Glucopyranoside, 1,3,4,6-tetrakis-O-(trimethylsilyl)-β-D-fructofuranosyl 2,3,4,6-tetrakis-O-(trimethylsilyl)- |
| FT719 | 0.1806 | NS | 0.481 | -1.0565 | 333.13 | 486.0000 |  | unindentified |
| FT725 | 1.6408 | 3.86E-02 | 2.8875 | 1.5298 | 341.00 | 546.6301 | 360 | 1-Silacyclo-2,4-hexadiene |
| FT736 | 1.3891 | NS | 0.045 | -4.4642 | 361.14 | 442.3656 |  | unindentified |
| FT745 | 1.0334 | NS | 0.246 | -2.0224 | 363.13 | 485.5000 |  | unindentified |
| FT746 | 0.3967 | NS | 0.394 | -1.3422 | 363.18 | 485.3333 |  | unindentified |
| FT750 | 1.4483 | NS | 0.285 | -1.8107 | 365.15 | 446.9551 | 648 | Ribitol, 1,2,3,4,5-pentakis-O-(trimethylsilyl)- |
| FT751 | 1.5605 | NS | 0.216 | -2.2082 | 371.26 | 407.0411 | 796 | Silane, diethyldodecyloxyoctyloxy- |
| FT769 | 1.6873 | 2.90E-02 | 4.3187 | 2.1106 | 438.21 | 441.3501 | 862 | D-(+)-Trehalose, octakis (trimethylsilyl) ether |
| FT773 | 0.4351 | NS | 2.4562 | 1.2964 | 450.24 | 441.9053 |  | unindentified |

**Table S4**. Differential metabolites in the *A. brasilense* Ab-V5 culture at FORM15 compared to the uninoculated medium after an incubation period of 12 h at 180 rpm and 28 ºC. Peak ID indicates the peak number after XCMS analysis of the chromatograms. VIP score denotes the value of variable importance in projection, considering the component 1 of multivariate supervised discriminant analysis by partial least squares (sPLS-DA). The *p*-value indicates whether a significant difference (p < 0.05) or a non-significant difference (NS) is present according to Student's t-test. FC stands for fold-change analysis, which is the ratio between group means using data prior to column-wise normalization. Log2(FC) indicates the log2 transformed value for each comparison. Match Factor indicates the similarity score of a peak to the listed compounds in the NIST Mass Spectral Library (excellent match: >900; good match: 800–900; fair match: 700–800; medium to low confidence: <700).

| Peak ID | VIP score | *p*-value | FC | log2(FC) | *m/z* | RT (s) | Match Factor | Compound |
| --- | --- | --- | --- | --- | --- | --- | --- | --- |
| FT003 | 1.8044 | 1.76E-02 | 3.5591 | 1.8315 | 44.95 | 359.2051 | 702 | Sym-tetramethyl (diisopropyl)disiloxane |
| FT005 | 1.1523 | NS | 0.315 | -1.6651 | 44.99 | 407.8130 | 906 | Tyramine, trimethylsilyl ether |
| FT016 | 2.0002 | 2.98E-04 | 2.895 | 1.5336 | 50.00 | 300.8238 | 807 | Acetamide, N-(2,6-dimethylphenyl)-N-(2-methylphenyl)- Acetamide, N-(2,6-dimethylphenyl)-N-(2-methylphenyl)- |
| FT020 | 1.7026 | 3.66E-02 | 2.0035 | 1.0025 | 56.02 | 326.1130 | 781 | N-methyliminopropylbenzene |
| FT025 | 1.9827 | 7.56E-04 | 4.709 | 2.2354 | 57.05 | 404.6882 | 678 | Tyramine, trimethylsilyl ether |
| FT035 | 1.7359 | 2.97E-02 | 0.495 | -1.0146 | 59.01 | 433.9883 |  | unindentified |
| FT046 | 1.6401 | NS | 2.0339 | 1.0242 | 65.05 | 359.0704 | 727 | Sym-tetramethyl (diisopropyl)disiloxane |
| FT057 | 1.9823 | 7.70E-04 | 4.5238 | 2.1775 | 71.05 | 386.9210 | 859 | 1-Mono-isobutyrin, bis(trimethylsilyl)- deriv. |
| FT059 | 1.9670 | 1.36E-03 | 0.306 | -1.7057 | 71.09 | 434.0676 | 682 | 2-Thiobarbituric acid, tris(tert-butyldimethylsilyl) deriv. |
| FT062 | 1.3835 | NS | 2.5152 | 1.3307 | 72.05 | 348.0756 |  | unindentified |
| FT063 | 1.2150 | NS | 0.338 | -1.5638 | 72.15 | 348.1051 | 924 | Glycerol, tris(trimethylsilyl) ether |
| FT068 | 1.9034 | 5.58E-03 | 3.3603 | 1.7486 | 73.05 | 358.5492 | 910 | 1(2H)-Pentalenone, 3,3a,4,6a-tetrahydro- |
| FT071 | 1.2974 | NS | 0.321 | -1.6404 | 73.05 | 408.8817 | 901 | Tyramine, trimethylsilyl ether |
| FT077 | 1.8751 | 8.36E-03 | 2.651 | 1.4065 | 74.05 | 358.6306 | 882 | Benzeneacetic acid, trimethylsilyl ester |
| FT085 | 1.8845 | 7.37E-03 | 3.1486 | 1.6547 | 75.03 | 358.9076 | 727 | Sym-tetramethyl(diisopropyl)disiloxane |
| FT091 | 1.9758 | 1.00E-03 | 3.4228 | 1.7752 | 77.04 | 344.0441 | 930 | Benzoic acid trimethylsilyl ester |
| FT104 | 1.9754 | 1.01E-03 | 7.6934 | 2.9436 | 85.08 | 404.6882 | 678 | Tyramine, trimethylsilyl ether |
| FT105 | 1.4715 | NS | 0.332 | -1.5921 | 85.10 | 416.2404 | 603 | Acetonitrile, 2-phenyl-2-trimethylsilyloxy- |
| FT108 | 1.8769 | 8.16E-03 | 5.0728 | 2.3428 | 86.08 | 404.7878 | 662 | Bis(tert-butyldimethylsilyl) 3,6,9,12-tetraoxatetradecane-1,14-dioate |
| FT123 | 1.2797 | NS | 0.428 | -1.2254 | 89.06 | 408.1745 | 782 | 2-Piperidinecarboxylic acid, trimethylsilyl ester |
| FT136 | 1.2458 | NS | 0.224 | -2.1567 | 91.09 | 484.6124 |  | unindentified |
| FT139 | 1.2535 | NS | 2.051 | 1.0363 | 92.09 | 354.7446 | 748 | Benzeneethanamine |
| FT141 | 1.0198 | NS | 0.351 | -1.5118 | 93.02 | 300.9320 | 807 | Acetamide, N-(2,6-dimethylphenyl)-N-(2-methylphenyl)- |
| FT148 | 1.6961 | 3.81E-02 | 0.149 | -2.7411 | 99.10 | 433.8238 | 758 | Furan, 2,5-dihydro-2,5-dimethoxy- |
| FT149 | 1.5035 | NS | 0.337 | -1.5678 | 99.10 | 422.0801 | 645 | 4-Amino-4,5(1H)-dihydro-1,2,4-triazole-5-one |
| FT152 | 1.8782 | 8.02E-03 | 8.2949 | 3.0522 | 100.10 | 429.7755 |  | unindentified |
| FT153 | 1.6845 | 4.07E-02 | 0.308 | -1.6964 | 100.09 | 434.1112 |  | unindentified |
| FT154 | 1.4330 | NS | 4.9097 | 2.2956 | 100.10 | 487.0781 | 837 | Ethyl 2,3,4,6-tetrakis-O-(trimethylsilyl)-D-glucopyranoside |
| FT158 | 1.4811 | NS | 0.483 | -1.0482 | 101.09 | 433.9883 | 688 | Furan, 2,5-dihydro-2,5-dimethoxy- |
| FT163 | 1.3527 | NS | 0.245 | -2.025 | 102.10 | 433.9338 |  | unindentified |
| FT168 | 1.8211 | 1.51E-02 | 0.424 | -1.2375 | 103.10 | 434.0925 | 682 | 2-Thiobarbituric acid, tris(tert-butyldimethylsilyl) deriv |
| FT177 | 2.0204 | 2.33E-05 | 4.6855 | 2.2282 | 105.10 | 344.0783 | 867 | Benzoic acid trimethylsilyl ester |
| FT178 | 1.7396 | 2.90E-02 | 2.2884 | 1.1944 | 105.10 | 428.7658 | 861 | N-(2-Methoxyphenyl)-2-nitrobenzenecarbothioamide |
| FT180 | 1.9223 | 3.99E-03 | 2.4129 | 1.2708 | 106.09 | 344.5408 | 932 | Benzoic acid trimethylsilyl ester |
| FT181 | 1.5679 | NS | 2.3202 | 1.2143 | 106.10 | 428.3851 | 347 | 2-Methylbenzyl p-toluate |
| FT200 | 1.3472 | NS | 0.345 | -1.536 | 113.10 | 348.0380 | 727 | 2(3H)-Furanone, 3-[(4-nitrophenyl)methylene]-5-phenyl- |
| FT214 | 1.7903 | 1.99E-02 | 0.402 | -1.316 | 116.10 | 433.2574 | 749 | Furan, 2,5-dihydro-2,5-dimethoxy- |
| FT220 | 0.7497 | NS | 0.476 | -1.0707 | 117.10 | 531.9411 | 371 | N-Cyclohexyl-1-pyrrolidinecarbothioamide |
| FT223 | 0.9198 | NS | 0.419 | -1.2549 | 118.10 | 532.9350 | 513 | 1,2-Cyclohexanedicarboxylic acid, 2,6-dimethoxyphenyl heptyl ester |
| FT231 | 1.7157 | 3.38E-02 | 2.5118 | 1.3287 | 120.10 | 441.9036 |  | unindentified |
| FT269 | 1.2904 | NS | 0.441 | -1.1799 | 132.06 | 348.0265 |  | unindentified |
| FT286 | 1.9784 | 9.04E-04 | 3.9191 | 1.9705 | 135.10 | 344.0949 | 856 | Benzoic acid trimethylsilyl ester |
| FT287 | 1.5757 | NS | 2.2259 | 1.1544 | 135.08 | 469.3414 | 704 | Adenine |
| FT290 | 1.9719 | 1.15E-03 | 2.5799 | 1.3673 | 137.08 | 359.1714 | 727 | Sym-tetramethyl(diisopropyl)disiloxane |
| FT319 | 1.7732 | 2.27E-02 | 0.336 | -1.5736 | 147.09 | 433.9882 |  | unindentified |
| FT321 | 1.8658 | 9.38E-03 | 2.7822 | 1.4762 | 147.09 | 405.2682 | 678 | Tyramine, trimethylsilyl ether |
| FT329 | 1.2377 | NS | 0.340 | -1.5552 | 149.06 | 407.8980 |  | unindentified |
| FT346 | 1.0066 | NS | 2.0294 | 1.0211 | 154.09 | 483.2593 | 682 | 7-Isoquinolinol, 1,2,3,4-tetrahydro-6-methoxy-2-methyl-1-[2-(3,4,5-trimethoxyphenyl)ethyl]- |
| FT358 | 1.7356 | 2.97E-02 | 2.5217 | 1.3344 | 159.09 | 404.7100 | 678 | Tyramine, trimethylsilyl ether |
| FT369 | 1.9211 | 4.12E-03 | 3.8972 | 1.9624 | 164.10 | 358.1909 | 766 | Sym-tetramethyl (diisopropyl)disiloxane |
| FT371 | 1.0512 | NS | 0.193 | -2.3703 | 165.10 | 406.9612 | 598 | 2,3,4,5-Tetrahydropyridazine |
| FT375 | 1.2618 | NS | 0.278 | -1.8473 | 166.09 | 408.1745 | 753 | Hexadecanoic acid, 2,3-bis[(trimethylsilyl)oxy]propyl ester |
| FT378 | 1.5725 | NS | 2.3318 | 1.2215 | 167.09 | 551.1802 |  | unindentified |
| FT382 | 0.6337 | NS | 2.5412 | 1.3455 | 169.10 | 441.9044 |  | unindentified |
| FT406 | 1.4359 | NS | 0.188 | -2.4083 | 177.10 | 407.4672 |  | unindentified |
| FT412 | 1.9979 | 3.37E-04 | 5.2488 | 2.392 | 179.05 | 344.0099 | 776 | Benzeneethanamine |
| FT414 | 0.7467 | NS | 0.421 | -1.2473 | 179.10 | 406.0970 |  | unindentified |
| FT418 | 1.9739 | 1.07E-03 | 3.0967 | 1.6307 | 180.06 | 344.0100 | 729 | Pyridine, 6-methyl-2-[(2,3-dimethyl-2-butyl)dimethylsilyloxy]- |
| FT420 | 1.1040 | NS | 0.375 | -1.4129 | 180.10 | 407.4287 | 717 | Silane, dimethyl-2-propenyl(tetradecyloxy)- |
| FT422 | 1.3244 | NS | 0.105 | -3.2512 | 180.11 | 409.6467 | 701 | Tyramine, trimethylsilyl ether |
| FT425 | 1.1427 | NS | 0.225 | -2.1502 | 181.10 | 407.4287 |  | unindentified |
| FT453 | 1.9451 | 2.49E-03 | 3.5881 | 1.8432 | 193.08 | 359.0704 | 887 | Benzeneacetic acid, trimethylsilyl ester |
| FT455 | 1.4684 | NS | 4.6159 | 2.2066 | 193.10 | 424.6939 | 766 | Benzenepropanoic acid, α-[(trimethylsilyl)oxy]-, trimethylsilyl ester |
| FT460 | 1.3728 | NS | 0.230 | -2.1203 | 194.09 | 408.1745 |  | unindentified |
| FT468 | 1.0507 | NS | 3.4348 | 1.7802 | 199.07 | 447.0001 | 676 | D-Turanose, heptakis(trimethylsilyl)- |
| FT479 | 1.6398 | NS | 0.364 | -1.456 | 203.09 | 432.5015 | 783 | Furan, 2,5-dihydro-2,5-dimethoxy- |
| FT480 | 1.8376 | 1.29E-02 | 4.0784 | 2.028 | 203.09 | 404.7100 | 678 | Tyramine, trimethylsilyl ether |
| FT481 | 1.9488 | 2.28E-03 | 5.4842 | 2.4553 | 203.10 | 387.3405 | 893 | 1-Mono-isobutyrin, bis(trimethylsilyl)- deriv. |
| FT484 | 1.1857 | NS | 0.432 | -1.2097 | 204.09 | 509.0190 | 525 | Phenylthioacetic acid, 4-cyanophenyl ester |
| FT487 | 1.1131 | NS | 0.472 | -1.082 | 204.11 | 508.7986 | 751 | Gulose, 2,3,4,5,6-pentakis-O-(trimethylsilyl)- |
| FT497 | 0.5661 | NS | 0.495 | -1.0144 | 207.00 | 532.7247 | 482 | Mannose, 6-deoxy-2,3,4,5-tetrakis-O-(trimethylsilyl)-, L- |
| FT521 | 0.8618 | NS | 3.0817 | 1.6237 | 217.10 | 441.9198 |  | unindentified |
| FT522 | 1.5445 | NS | 0.412 | -1.2785 | 217.10 | 508.9257 | 601 | β-Gentiobiose, octakis(trimethylsilyl) ether |
| FT523 | 1.6382 | NS | 8.7501 | 3.1293 | 217.09 | 404.6749 | 531 | Benzeneethanamine, N-[(pentafluorophenyl)methylene]-4-[(trimethylsilyl)oxy]- |
| FT524 | 1.7435 | 2.82E-02 | 0.294 | -1.7679 | 217.09 | 418.0645 | 788 | Sucrose, octakis (trimethylsilyl) ether |
| FT532 | 1.9223 | 4.03E-03 | 4.172 | 2.0607 | 218.15 | 404.7660 | 703 | 1-Cyclohexyldimethylsilyloxy-2-phenylethane |
| FT533 | 1.2289 | NS | 0.372 | -1.4264 | 219.09 | 348.1056 | 556 | Benzene, 1,1'-[1-(2-propenyl)-1,2-ethanediyl]bis- |
| FT539 | 1.2146 | NS | 0.327 | -1.6138 | 220.09 | 348.0265 |  | unindentified |
| FT550 | 1.7617 | 2.48E-02 | 2.1688 | 1.1169 | 223.10 | 458.9928 | 518 | 1,4-Dioxaspiro[4.5]decane, 8-chloro- |
| FT563 | 1.0121 | NS | 2.2734 | 1.1848 | 230.10 | 442.9854 | 717 | 3-Tetradecene, (Z)- |
| FT567 | 1.5552 | NS | 0.192 | -2.379 | 231.10 | 433.3596 |  | unindentified |
| FT571 | 1.9442 | 2.55E-03 | 0.129 | -2.9552 | 231.15 | 433.9338 | 685 | Benzenamine, N,N'-(1,2-dimethyl-1,2-ethanediylidene)bis[2,6-bis(1-methylethyl)- |
| FT576 | 1.9432 | 2.61E-03 | 0.179 | -2.4791 | 232.15 | 434.0676 |  | unindentified |
| FT596 | 1.5995 | NS | 5.7057 | 2.5124 | 243.10 | 441.9198 | 707 | Sucrose, octakis(trimethylsilyl) ether |
| FT601 | 1.3664 | NS | 2.0039 | 1.0028 | 244.10 | 441.9053 |  | unindentified |
| FT611 | 1.8903 | 6.79E-03 | 2.2191 | 1.150 | 251.09 | 482.0141 | 741 | β-D-Mannopyranoside, methyl 2,3,4,6-tetrakis-O-(trimethylsilyl)- |
| FT638 | 1.8492 | 1.14E-02 | 4.0789 | 2.0282 | 271.10 | 441.9160 | 773 | Sucrose, octakis(trimethylsilyl) ether |
| FT639 | 1.2646 | NS | 2.2285 | 1.1561 | 271.09 | 403.7614 | 637 | Tyramine, trimethylsilyl ether |
| FT658 | 1.0598 | NS | 16.921 | 4.0808 | 289.10 | 447.0771 | 648 | Ribitol, 1,2,3,4,5-pentakis-O-(trimethylsilyl)- |
| FT660 | 1.0845 | NS | 2.4145 | 1.2717 | 289.10 | 486.0000 | 832 | Ethyl 2,3,4,6-tetrakis-O-(trimethylsilyl)-D-glucopyranoside |
| FT662 | 0.8055 | NS | 12.553 | 3.650 | 289.13 | 445.8668 | 634 | D-(+)-Trehalose, octakis(trimethylsilyl) ether |
| FT665 | 0.7836 | NS | 9.1626 | 3.1958 | 290.10 | 446.8869 | 703 | 3,6-Dioxa-2,7-disilaoctane, 2,2,4,7,7-pentamethyl- |
| FT702 | 1.3572 | NS | 3.6551 | 1.8699 | 319.10 | 442.0628 | 829 | α-D-Glucopyranoside, 1,3,4,6-tetrakis-O-(trimethylsilyl)-β-D-fructofuranosyl 2,3,4,6-tetrakis-O-(trimethylsilyl)- |
| FT704 | 0.9564 | NS | 2.5479 | 1.3493 | 319.15 | 441.3573 | 907 | Sucrose, octakis(trimethylsilyl) ether |
| FT727 | 1.6319 | NS | 0.161 | -2.6302 | 341.15 | 530.7080 | 668 | 9,12-Octadecadienoic acid (Z,Z)-, trimethylsilyl ester |
| FT735 | 0.6803 | NS | 0.439 | -1.1889 | 361.13 | 420.0736 | 573 | 2-Amino-1-phenylethanol, trimethylsilyl ether |
| FT736 | 0.3970 | NS | 3.818 | 1.9328 | 361.14 | 442.3656 |  | unindentified |
| FT737 | 0.9788 | NS | 9.4969 | 3.2475 | 361.20 | 441.9044 |  | unindentified |
| FT744 | 0.8809 | NS | 3.632 | 1.8607 | 363.11 | 441.9236 | 603 | α-D-Glucopyranoside, 1,3,4,6-tetrakis-O-(trimethylsilyl)-β-D-fructofuranosyl 2,3,4,6-tetrakis-O-(trimethylsilyl)- |
| FT749 | 0.8542 | NS | 2.2578 | 1.1749 | 364.17 | 441.1653 |  | unindentified |
| FT750 | 1.6295 | NS | 12.193 | 3.608 | 365.15 | 446.9551 | 648 | Ribitol, 1,2,3,4,5-pentakis-O-(trimethylsilyl)- |
| FT764 | 1.7597 | 2.51E-02 | 7.3265 | 2.8731 | 437.15 | 442.2019 | 764 | Sucrose, octakis(trimethylsilyl) ether |
| FT766 | 1.1916 | NS | 0.456 | -1.1341 | 437.20 | 441.3501 |  | unindentified |
| FT768 | 1.6411 | NS | 2.9014 | 1.5367 | 438.13 | 442.0627 |  | unindentified |
| FT769 | 1.1211 | NS | 0.412 | -1.2789 | 438.21 | 441.3501 | 862 | D-(+)-Trehalose, octakis (trimethylsilyl) ether |
| FT770 | 1.5322 | NS | 2.0814 | 1.0576 | 439.15 | 442.2019 |  | unindentified |

**Table S5**. Differential metabolites in the *A. brasilense* Ab-V5 culture at FORM15 compared to the uninoculated medium after an incubation period of 36 h at 180 rpm and 28 ºC. Peak ID indicates the peak number after XCMS analysis of the chromatograms. VIP score denotes the value of variable importance in projection, considering the component 1 of multivariate supervised discriminant analysis by partial least squares (sPLS-DA). The *p*-value indicates whether a significant difference (p < 0.05) or a non-significant difference (NS) is present according to Student's t-test. FC stands for fold-change analysis, which is the ratio between group means using data prior to column-wise normalization. Log2(FC) indicates the log2 transformed value for each comparison. Match Factor indicates the similarity score of a peak to the listed compounds in the NIST Mass Spectral Library (excellent match: >900; good match: 800–900; fair match: 700–800; medium to low confidence: <700).

| Peak ID | VIP score | *p*-value | FC | log2(FC) | *m/z* | RT (s) | Match Factor | Compounds |
| --- | --- | --- | --- | --- | --- | --- | --- | --- |
| FT001 | 1.2228 | NS | 0.493 | -1.0191 | 44.99 | 348.0756 |  | unindentified |
| FT008 | 1.2237 | NS | 0.435 | -1.2007 | 45.99 | 348.0509 | 808 | Glycerol, tris(trimethylsilyl) ether |
| FT010 | 1.2218 | NS | 0.465 | -1.1047 | 46.95 | 348.0387 | 950 | Glycerol, tris(trimethylsilyl) ether |
| FT020 | 1.1796 | NS | 0.469 | -1.0922 | 56.02 | 326.1130 | 781 | N-methyliminopropylbenzene |
| FT022 | 1.6186 | 8.80E-04 | 3.0216 | 1.5953 | 56.05 | 486.5014 | 576 | Methylphosphonic acid, fluoroanhydride, 3-methyl-1-(2-methylpropyl)butyl ester |
| FT023 | 1.5901 | 2.49E-03 | 2.9796 | 1.5751 | 56.05 | 427.0527 | 372 | 1H-Isoindole-1,3(2H)-dione, 2-(7-azabicyclo[2.2.1]hept-1-en-7-yl)- |
| FT025 | 1.1349 | NS | 5.4843 | 2.4553 | 57.05 | 404.6882 | 678 | Tyramine, trimethylsilyl ether |
| FT026 | 1.2477 | NS | 3.4802 | 1.7992 | 57.05 | 390.3981 | 513 | 1H-Tetrazol-5-amine |
| FT027 | 1.1859 | NS | 0.357 | -1.4851 | 58.01 | 348.0504 | 768 | 2-Phenylamino-6,7-dihydro-5H-1-thia-8-aza-S-indacene-3-carbaldehyde |
| FT036 | 1.1427 | NS | 0.422 | -1.2434 | 60.00 | 348.0504 | 750 | Glycerol, tris(trimethylsilyl) ether |
| FT042 | 1.2456 | NS | 0.342 | -1.5457 | 63.00 | 300.1271 | 931 | Benzeneethanamine |
| FT045 | 1.2168 | NS | 0.398 | -1.3299 | 65.00 | 300.0397 |  | unindentified |
| FT057 | 1.0753 | NS | 3.7262 | 1.8977 | 71.05 | 386.9210 | 859 | 1-Mono-isobutyrin, bis(trimethylsilyl)- deriv. |
| FT059 | 1.6376 | 2.46E-04 | 0.308 | -1.6992 | 71.09 | 434.0676 | 682 | 2-Thiobarbituric acid, tris(tert-butyldimethylsilyl) deriv. |
| FT063 | 1.1078 | NS | 0.024 | -5.3869 | 72.15 | 348.1051 | 924 | Glycerol, tris(trimethylsilyl) ether |
| FT065 | 1.2113 | NS | 0.463 | -1.1101 | 73.05 | 348.0756 |  | unindentified |
| FT068 | 1.5115 | 1.15E-02 | 3.3191 | 1.7308 | 73.05 | 358.5492 | 910 | 1(2H)-Pentalenone, 3,3a,4,6a-tetrahydro- |
| FT070 | 1.2149 | NS | 2.0049 | 1.0036 | 73.05 | 327.1555 | 792 | 3,6,9-Trioxa-2,10-disilaundecane, 2,2,10,10-tetramethyl- |
| FT071 | 1.2647 | NS | 0.392 | -1.3524 | 73.05 | 408.8817 | 901 | Tyramine, trimethylsilyl ether |
| FT073 | 1.0947 | NS | 11.104 | 3.473 | 73.05 | 333.8488 | 629 | Butane, 2-isocyanato- |
| FT074 | 1.3116 | NS | 0.455 | -1.1365 | 74.05 | 348.1003 |  | unindentified |
| FT077 | 1.5093 | 1.18E-02 | 2.7313 | 1.4496 | 74.05 | 358.6306 | 882 | Benzeneacetic acid, trimethylsilyl ester |
| FT082 | 1.2260 | NS | 9.0012 | 3.1701 | 75.05 | 333.8488 |  | unindentified |
| FT083 | 1.2252 | NS | 0.401 | -1.318 | 75.04 | 348.0509 |  | unindentified |
| FT084 | 1.4651 | 1.97E-02 | 0.201 | -2.312 | 75.02 | 532.0743 | 679 | Octadecanoic acid, trimethylsilyl ester |
| FT090 | 1.3283 | NS | 0.302 | -1.7287 | 76.03 | 531.9411 | 812 | 9,12-Octadecadienoic acid (Z,Z)-, trimethylsilyl ester |
| FT093 | 1.0751 | NS | 0.286 | -1.8038 | 77.01 | 417.0645 | 697 | Phenol, 4-(2-aminoethyl)- |
| FT099 | 1.1860 | NS | 0.434 | -1.2053 | 84.05 | 410.5290 |  | unindentified |
| FT103 | 1.2736 | NS | 0.442 | -1.1775 | 85.05 | 348.1603 |  | unindentified |
| FT104 | 1.0527 | NS | 9.4496 | 3.2403 | 85.08 | 404.6882 | 678 | Tyramine, trimethylsilyl ether |
| FT105 | 1.0788 | NS | 0.298 | -1.7456 | 85.10 | 416.2404 | 603 | Acetonitrile, 2-phenyl-2-trimethylsilyloxy- |
| FT106 | 1.2056 | NS | 4.2027 | 2.0713 | 85.05 | 390.3981 | 607 | Pentanethioic acid, S-ethyl ester |
| FT107 | 1.2203 | NS | 0.254 | -1.978 | 86.10 | 322.1338 | 628 | tert-Butyl-[2-[2-(2-ethoxyethoxy)ethoxy]ethoxy]dimethylsilane |
| FT108 | 1.2569 | NS | 3.5968 | 1.8467 | 86.08 | 404.7878 | 662 | Bis(tert-butyldimethylsilyl) 3,6,9,12-tetraoxatetradecane-1,14-dioate |
| FT111 | 1.1035 | NS | 0.304 | -1.7197 | 86.10 | 315.0363 | 743 | l-Leucine, trimethylsilyl ester |
| FT112 | 1.4225 | 2.90E-02 | 2.5087 | 1.3269 | 87.04 | 486.0000 |  | unindentified |
| FT115 | 1.1309 | NS | 0.381 | -1.3914 | 88.05 | 348.0498 |  | unindentified |
| FT116 | 1.2343 | NS | 3.4681 | 1.7941 | 88.10 | 486.0066 | 802 | Gulose, 2,3,4,5,6-pentakis-O-(trimethylsilyl)- |
| FT117 | 1.6318 | 3.98E-04 | 5.5857 | 2.4817 | 88.10 | 426.7490 | 761 | N-(2-Methoxyphenyl)-2-nitrobenzenecarbothioamide |
| FT125 | 1.1195 | NS | 0.446 | -1.164 | 90.05 | 326.0347 | 447 | 3,6,9-Trioxa-2,10-disilaundecane, 2,2,10,10-tetramethyl- |
| FT129 | 1.4006 | 3.45E-02 | 0.490 | -1.0278 | 90.08 | 408.5278 |  | unindentified |
| FT135 | 1.5844 | 2.98E-03 | 14.473 | 3.8553 | 91.10 | 353.8015 | 794 | Benzeneethanamine |
| FT136 | 0.8011 | NS | 0.355 | -1.4956 | 91.09 | 484.6124 |  | unindentified |
| FT137 | 1.3044 | NS | 0.018 | -5.7884 | 91.10 | 300.0405 |  | unindentified |
| FT139 | 1.4466 | 2.35E-02 | 12.025 | 3.588 | 92.09 | 354.7446 | 748 | Benzeneethanamine |
| FT141 | 1.1863 | NS | 0.203 | -2.297 | 93.02 | 300.9320 | 807 | Acetamide, N-(2,6-dimethylphenyl)-N-(2-methylphenyl)- |
| FT148 | 0.7846 | NS | 0.083 | -3.5923 | 99.10 | 433.8238 | 758 | Furan, 2,5-dihydro-2,5-dimethoxy- |
| FT151 | 0.6009 | NS | 3.610 | 1.852 | 100.08 | 422.2989 | 948 | 4-Amino-4,5(1H)-dihydro-1,2,4-triazole-5-one |
| FT152 | 1.5285 | 9.03E-03 | 82.490 | 6.3661 | 100.10 | 429.7755 |  | unindentified |
| FT153 | 1.0317 | NS | 0.490 | -1.0284 | 100.09 | 434.1112 |  | unindentified |
| FT154 | 1.6270 | 5.53E-04 | 55.887 | 5.8044 | 100.10 | 487.0781 | 837 | Ethyl 2,3,4,6-tetrakis-O-(trimethylsilyl)-D-glucopyranoside |
| FT157 | 0.8085 | NS | 2.1481 | 1.1031 | 101.09 | 327.0030 | 411 | Butane, 1,2,3-tris(trimethylsiloxy)- |
| FT158 | 1.6220 | 7.35E-04 | 0.321 | -1.6413 | 101.09 | 433.9883 | 688 | Furan, 2,5-dihydro-2,5-dimethoxy- |
| FT159 | 0.9753 | NS | 2.3696 | 1.2446 | 101.09 | 404.8656 | 662 | Bis(tert-butyldimethylsilyl) 3,6,9,12-tetraoxatetradecane-1,14-dioate |
| FT160 | 0.6668 | NS | 2.4944 | 1.3187 | 101.10 | 429.7755 | 347 | 2-Methylbenzyl p-toluate |
| FT163 | 1.4721 | 1.83E-02 | 0.239 | -2.0664 | 102.10 | 433.9338 |  | unindentified |
| FT165 | 1.1968 | NS | 0.367 | -1.4445 | 102.14 | 348.0498 |  | unindentified |
| FT166 | 1.2392 | NS | 0.474 | -1.0753 | 103.10 | 348.0756 |  | unindentified |
| FT168 | 1.3370 | NS | 0.431 | -1.2155 | 103.10 | 434.0925 | 682 | 2-Thiobarbituric acid, tris(tert-butyldimethylsilyl) deriv |
| FT175 | 0.8589 | NS | 0.271 | -1.8834 | 105.05 | 321.1933 | 567 | tert-Butyl-[2-[2-(2-ethoxyethoxy)ethoxy]ethoxy]dimethylsilane |
| FT178 | 1.6321 | 3.90E-04 | 19.415 | 4.2791 | 105.10 | 428.7658 | 861 | N-(2-Methoxyphenyl)-2-nitrobenzenecarbothioamide |
| FT181 | 1.6498 | 4.46E-05 | 4.0775 | 2.0277 | 106.10 | 428.3851 | 347 | 2-Methylbenzyl p-toluate |
| FT184 | 0.9511 | NS | 0.195 | -2.3601 | 107.09 | 417.0645 | 697 | Phenol, 4-(2-aminoethyl)- |
| FT186 | 1.4923 | 1.46E-02 | 2.7882 | 1.4793 | 107.10 | 478.9640 | 682 | Glycyl-L-tyrosine |
| FT187 | 1.4093 | 3.23E-02 | 4.4192 | 2.1438 | 107.10 | 486.4315 | 630 | Ethyl 2,3,4,6-tetrakis-O-(trimethylsilyl)-D-glucopyranoside |
| FT190 | 1.1051 | NS | 0.133 | -2.9096 | 108.09 | 417.0645 |  | unindentified |
| FT195 | 1.1445 | NS | 2.3528 | 1.2344 | 111.10 | 485.9875 | 530 | Ethyl 2,3,4,6-tetrakis-O-(trimethylsilyl)-D-glucopyranoside |
| FT198 | 1.2338 | NS | 0.378 | -1.4024 | 113.05 | 348.0509 |  | unindentified |
| FT203 | 1.5261 | 9.35E-03 | 0.412 | -1.2795 | 113.14 | 450.8045 | 449 | Benzene, 1,3-bis(1-allyl-5-tetrazolylthiomethyl)- |
| FT210 | 1.2114 | NS | 0.389 | -1.3618 | 115.06 | 348.0265 |  | unindentified |
| FT215 | 0.9335 | NS | 20.771 | 4.3765 | 116.10 | 333.8488 |  | unindentified |
| FT216 | 1.1590 | NS | 0.359 | -1.4788 | 116.14 | 348.1056 | 934 | Silanol, trimethyl-, phosphate (3:1) |
| FT217 | 1.2026 | NS | 0.472 | -1.0827 | 117.10 | 348.0756 |  | unindentified |
| FT220 | 0.9061 | NS | 0.263 | -1.9259 | 117.10 | 531.9411 | 371 | N-Cyclohexyl-1-pyrrolidinecarbothioamide |
| FT223 | 1.1594 | NS | 0.356 | -1.4891 | 118.10 | 532.9350 | 513 | 1,2-Cyclohexanedicarboxylic acid, 2,6-dimethoxyphenyl heptyl ester |
| FT225 | 0.5718 | NS | 0.474 | -1.0761 | 118.10 | 348.0262 |  | unindentified |
| FT226 | 1.2420 | NS | 0.398 | -1.3279 | 119.06 | 348.1003 |  | unindentified |
| FT232 | 1.4261 | 2.53E-02 | 2.7553 | 1.4622 | 120.10 | 483.9204 | 618 | l-Alanine, N-(4-ethylbenzoyl)-, decyl ester |
| FT233 | 1.5853 | 2.95E-03 | 8.9161 | 3.1564 | 121.10 | 486.3158 | 630 | Ethyl 2,3,4,6-tetrakis-O-(trimethylsilyl)-D-glucopyranoside |
| FT245 | 1.3280 | NS | 0.283 | -1.8194 | 127.10 | 553.2478 | 609 | 3,6-Dibutyl-1,2-dihydro-1,2,4,5-tetrazine |
| FT246 | 1.5794 | 3.45E-03 | 0.391 | -1.3533 | 127.13 | 467.1968 | 465 | Benzene, 2-(1-decylundecyl)-1,4-dimethyl- |
| FT250 | 1.2231 | NS | 0.327 | -1.6144 | 129.10 | 348.0504 | 750 | Glycerol, tris(trimethylsilyl) ether |
| FT252 | 1.4379 | 2.56E-02 | 0.284 | -1.8145 | 129.10 | 532.0743 |  | unindentified |
| FT253 | 1.5167 | 1.08E-02 | 2.2426 | 1.1651 | 129.10 | 486.0000 | 796 | Ethyl 2,3,4,6-tetrakis-O-(trimethylsilyl)-D-glucopyranoside |
| FT254 | 1.3402 | NS | 5.9337 | 2.5689 | 129.11 | 429.7755 | 661 | N-(2-Methoxyphenyl)-2-nitrobenzenecarbothioamide # |
| FT258 | 1.2136 | NS | 0.398 | -1.3297 | 130.09 | 347.9841 | 948 | Glycerol, tris(trimethylsilyl) ether |
| FT259 | 1.4317 | 2.70E-02 | 2.9485 | 1.560 | 130.10 | 469.3359 | 703 | Alanine, N-methyl-N-ethoxycarbonyl-, dodecyl ester |
| FT260 | 1.5046 | 1.27E-02 | 2.7227 | 1.445 | 130.09 | 425.8345 | 828 | Benzenepropanoic acid, α-[(trimethylsilyl)oxy]-, trimethylsilyl ester |
| FT262 | 1.2427 | NS | 0.335 | -1.5795 | 131.04 | 348.0509 |  | unindentified |
| FT265 | 1.4764 | 1.76E-02 | 2.2042 | 1.1402 | 131.08 | 327.1555 | 735 | Pantoyl lactone, trimethylsilyl |
| FT266 | 1.3726 | 4.23E-02 | 0.493 | -1.0203 | 131.10 | 533.2920 |  | unindentified |
| FT273 | 1.4630 | 2.02E-02 | 0.433 | -1.2071 | 132.10 | 504.0042 | 788 | Hexadecanoic acid, trimethylsilyl ester |
| FT274 | 1.3808 | 4.00E-02 | 0.350 | -1.5137 | 132.10 | 532.9350 | 740 | Octadecanoic acid, trimethylsilyl ester |
| FT275 | 1.1865 | NS | 0.422 | -1.2454 | 132.13 | 347.9841 | 790 | Glycerol, tris(trimethylsilyl) ether |
| FT276 | 1.1938 | NS | 0.472 | -1.0818 | 133.05 | 348.0756 |  | unindentified |
| FT278 | 0.9542 | NS | 2.2186 | 1.1497 | 133.05 | 327.03273 |  | unindentified |
| FT280 | 1.4150 | 3.10E-02 | 0.405 | -1.3027 | 133.07 | 433.9882 |  | unindentified |
| FT281 | 1.1967 | NS | 0.342 | -1.5484 | 134.05 | 348.0509 |  | unindentified |
| FT284 | 0.6547 | NS | 2.9241 | 1.548 | 134.10 | 388.0871 | 724 | Silane, trimethyl (tricyclo[3.3.1.1(3,7)]dec-2-yloxy)- |
| FT286 | 0.4118 | NS | 2.3135 | 1.2101 | 135.10 | 344.0949 | 856 | Benzoic acid trimethylsilyl ester |
| FT289 | 1.0250 | NS | 0.331 | -1.5934 | 137.10 | 417.0645 |  | unindentified |
| FT305 | 1.3352 | NS | 0.403 | -1.3116 | 143.10 | 328.1278 | 603 | Butane, 1,2,3-tris(trimethylsiloxy)- |
| FT309 | 1.2558 | NS | 0.354 | -1.4985 | 145.10 | 532.0743 | 371 | N-Cyclohexyl-1-pyrrolidinecarbothioamide |
| FT312 | 1.4470 | 2.36E-02 | 0.246 | -2.0235 | 145.10 | 318.0349 | 596 | 3-Methyl-1,2-bis(trimethylsilyl)butane |
| FT314 | 1.1999 | NS | 0.378 | -1.402 | 146.14 | 348.0498 |  | unindentified |
| FT316 | 1.1012 | NS | 16.871 | 4.0764 | 147.05 | 333.8488 |  | unindentified |
| FT317 | 1.2030 | NS | 0.463 | -1.1113 | 147.10 | 348.1003 |  | unindentified |
| FT319 | 1.2870 | NS | 0.437 | -1.1928 | 147.09 | 433.9882 |  | unindentified |
| FT321 | 0.9536 | NS | 4.174 | 2.0614 | 147.09 | 405.2682 | 678 | Tyramine, trimethylsilyl ether |
| FT322 | 1.2065 | NS | 0.479 | -1.0614 | 148.09 | 348.0756 |  | unindentified |
| FT327 | 1.3944 | 3.63E-02 | 0.423 | -1.241 | 148.10 | 433.9882 | 611 | Acetoacetic acid, bis(trimethylsilyl)- deriv. |
| FT328 | 1.6534 | 2.16E-05 | 6.2934 | 2.6538 | 148.10 | 426.9009 | 766 | Benzenepropanoic acid, α-[(trimethylsilyl)oxy]-, trimethylsilyl ester |
| FT330 | 1.2163 | NS | 0.499 | -1.0011 | 149.05 | 348.1056 |  | unindentified |
| FT333 | 1.2209 | NS | 4.2614 | 2.0913 | 149.10 | 332.0562 | 716 | 2-Ethyl-2-phenyl-1,3-benzodioxole |
| FT334 | 1.1507 | NS | 0.441 | -1.1795 | 150.07 | 348.0504 |  | unindentified |
| FT357 | 1.3268 | NS | 0.362 | -1.4648 | 159.05 | 348.0262 | 892 | 1-(1-Adamantyl)-2-triisopropylsilyloxyethane |
| FT358 | 0.9042 | NS | 3.4243 | 1.7758 | 159.09 | 404.7100 | 678 | Tyramine, trimethylsilyl ether |
| FT359 | 1.3063 | NS | 0.455 | -1.1373 | 159.10 | 348.3706 |  | unindentified |
| FT365 | 1.2397 | NS | 0.387 | -1.3682 | 163.05 | 348.0382 |  | unindentified |
| FT368 | 1.4979 | 1.38E-02 | 7.8528 | 2.9732 | 164.07 | 486.9657 | 641 | Di-N-propylethylamine |
| FT369 | 0.7654 | NS | 2.8302 | 1.5009 | 164.10 | 358.1909 | 766 | Sym-tetramethyl (diisopropyl)disiloxane |
| FT371 | 1.3850 | 3.88E-02 | 0.093 | -3.4223 | 165.10 | 406.9612 | 598 | 2,3,4,5-Tetrahydropyridazine |
| FT373 | 1.4991 | 1.36E-02 | 0.211 | -2.241 | 166.05 | 327.0345 | 799 | Pantoyl lactone, trimethylsilyl |
| FT375 | 1.3394 | NS | 0.313 | -1.6736 | 166.09 | 408.1745 | 753 | Hexadecanoic acid, 2,3-bis[(trimethylsilyl)oxy]propyl ester |
| FT389 | 1.4038 | 3.38E-02 | 0.421 | -1.2463 | 173.11 | 434.1125 |  | unindentified |
| FT396 | 1.2375 | NS | 0.373 | -1.4215 | 175.05 | 348.0504 |  | unindentified |
| FT399 | 1.0221 | NS | 0.488 | -1.0359 | 175.10 | 509.0191 | 806 | Mannose, 6-deoxy-2,3,4,5-tetrakis-O-(trimethylsilyl)-, L- |
| FT403 | 1.2381 | NS | 0.319 | -1.6481 | 177.05 | 348.0387 |  | unindentified |
| FT406 | 1.2047 | NS | 0.206 | -2.2776 | 177.10 | 407.4672 |  | unindentified |
| FT407 | 1.2713 | NS | 0.436 | -1.1977 | 178.06 | 348.0382 |  | unindentified |
| FT411 | 0.9978 | NS | 7.8233 | 2.9678 | 179.03 | 347.5509 |  | unindentified |
| FT414 | 0.9326 | NS | 0.359 | -1.4795 | 179.10 | 406.0970 |  | unindentified |
| FT420 | 1.5083 | 1.21E-02 | 0.052 | -4.2759 | 180.10 | 407.4287 | 717 | Silane, dimethyl-2-propenyl(tetradecyloxy)- |
| FT421 | 1.2173 | NS | 0.328 | -1.6087 | 180.10 | 420.8306 | 895 | 4-Hydroxyphenylethanol, di-TMS |
| FT422 | 1.5208 | 1.02E-02 | 0.083 | -3.5936 | 180.11 | 409.6467 | 701 | Tyramine, trimethylsilyl ether |
| FT423 | 1.5004 | 1.34E-02 | 0.358 | -1.4821 | 181.05 | 352.0240 | 691 | Thiophene, 2,5-bis(1,1-dimethylethyl)- |
| FT425 | 1.3497 | 4.91E-02 | 0.175 | -2.5119 | 181.10 | 407.4287 |  | unindentified |
| FT433 | 1.1941 | NS | 0.440 | -1.1836 | 189.05 | 348.0264 |  | unindentified |
| FT443 | 1.2710 | NS | 0.462 | -1.1129 | 191.05 | 348.0756 |  | unindentified |
| FT446 | 1.2086 | NS | 0.324 | -1.624 | 191.09 | 348.1984 |  | unindentified |
| FT449 | 1.2026 | NS | 0.449 | -1.1531 | 192.08 | 348.0504 |  | unindentified |
| FT450 | 0.9938 | NS | 0.434 | -1.2042 | 192.97 | 349.9706 | 908 | Silanol, trimethyl-, phosphate (3:1) |
| FT453 | 1.5369 | 8.00E-3 | 3.4972 | 1.8062 | 193.08 | 359.0704 | 887 | Benzeneacetic acid, trimethylsilyl ester |
| FT455 | 0.9398 | NS | 6.3586 | 2.6687 | 193.10 | 424.6939 | 766 | Benzenepropanoic acid, α-[(trimethylsilyl)oxy]-, trimethylsilyl ester |
| FT460 | 1.1766 | NS | 0.308 | -1.7002 | 194.09 | 408.1745 |  | unindentified |
| FT477 | 1.1434 | NS | 0.326 | -1.6179 | 203.09 | 348.0509 | 529 | Silanol, trimethyl-, phosphate (3:1) |
| FT479 | 1.5618 | 5.11E-03 | 0.230 | -2.1198 | 203.09 | 432.5015 | 749 | Furan, 2,5-dihydro-2,5-dimethoxy- |
| FT480 | 1.0948 | NS | 4.5804 | 2.1955 | 203.09 | 404.7100 | 678 | Tyramine, trimethylsilyl ether |
| FT481 | 1.5596 | 5.34E-03 | 7.2087 | 2.8497 | 203.10 | 387.3405 | 893 | 1-Mono-isobutyrin, bis(trimethylsilyl)- deriv. |
| FT483 | 1.2273 | NS | 0.256 | -1.968 | 203.10 | 348.1618 | 757 | Glycerol, tris(trimethylsilyl) ether |
| FT484 | 1.5832 | 3.13E-03 | 0.139 | -2.8454 | 204.09 | 509.0190 | 525 | Phenylthioacetic acid, 4-cyanophenyl ester |
| FT486 | 1.1363 | NS | 0.351 | -1.5114 | 204.10 | 348.0387 | 774 | Silane, trimethyl (propoxymethyl)- |
| FT487 | 1.6095 | 1.36E-03 | 0.135 | -2.8918 | 204.11 | 508.7986 | 751 | Gulose, 2,3,4,5,6-pentakis-O-(trimethylsilyl)- |
| FT488 | 1.2293 | NS | 0.275 | -1.8624 | 204.13 | 348.3706 | 757 | Glycerol, tris(trimethylsilyl) ether |
| FT493 | 1.2285 | NS | 0.455 | -1.1365 | 206.10 | 348.1303 | 551 | 2-Octen-1-ol |
| FT501 | 1.0965 | NS | 0.388 | -1.3644 | 207.09 | 348.0264 |  | unindentified |
| FT520 | 1.5814 | 3.29E-03 | 0.215 | -2.2147 | 217.10 | 457.2807 | 720 | Silane, diethylethoxyoctyloxy- |
| FT522 | 1.5643 | 4.86E-03 | 0.421 | -1.2468 | 217.10 | 508.9257 | 601 | β-Gentiobiose, octakis(trimethylsilyl) ether |
| FT523 | 1.1287 | NS | 17.876 | 4.1599 | 217.09 | 404.6749 | 531 | Benzeneethanamine, N-[(pentafluorophenyl)methylene]-4-[(trimethylsilyl)oxy]- |
| FT526 | 0.4478 | NS | 0.446 | -1.1661 | 217.10 | 348.0386 |  | unindentified |
| FT527 | 1.0179 | NS | 2.9855 | 1.578 | 217.10 | 432.6191 |  | unindentified |
| FT530 | 1.6465 | 9.46E-05 | 0.318 | -1.6537 | 218.09 | 456.8384 | 755 | D-(-)-Ribofuranose, tetrakis (trimethylsilyl) ether (isomer 2) |
| FT531 | 1.2418 | NS | 0.437 | -1.1943 | 218.10 | 348.0756 |  | unindentified |
| FT532 | 1.0385 | NS | 6.6924 | 2.7425 | 218.15 | 404.7660 | 703 | 1-Cyclohexyldimethylsilyloxy-2-phenylethane |
| FT538 | 1.2312 | NS | 0.280 | -1.8347 | 219.13 | 348.0498 |  | unindentified |
| FT539 | 1.1292 | NS | 0.403 | -1.3106 | 220.09 | 348.0265 |  | unindentified |
| FT544 | 1.2712 | NS | 0.424 | -1.2389 | 221.08 | 348.0380 |  | unindentified |
| FT548 | 0.9767 | NS | 0.472 | -1.0841 | 223.09 | 406.4707 |  | unindentified |
| FT564 | 1.1935 | NS | 5.548 | 2.472 | 230.10 | 432.6191 | 701 | Glucuronolactone, trisO-(trimethylsilyl)- |
| FT567 | 1.1479 | NS | 0.236 | -2.0851 | 231.10 | 433.3596 |  | unindentified |
| FT569 | 1.5218 | 1.01E-02 | 0.444 | -1.1712 | 231.13 | 455.8022 |  | unindentified |
| FT570 | 1.2439 | NS | 0.472 | -1.0826 | 231.10 | 485.5272 |  | unindentified |
| FT571 | 1.5677 | 4.52E-03 | 0.080 | -3.648 | 231.15 | 433.9338 | 685 | Benzenamine, N,N'-(1,2-dimethyl-1,2-ethanediylidene)bis[2,6-bis(1-methylethyl)- |
| FT576 | 1.5385 | 7.80E-03 | 0.219 | -2.1897 | 232.15 | 434.0676 |  | unindentified |
| FT594 | 0.8951 | NS | 2.5316 | 1.340 | 243.09 | 432.4280 |  | unindentified |
| FT598 | 1.0794 | NS | 2.048 | 1.0342 | 244.10 | 342.9062 | 877 | N-(3-Amino-2-hydroxy-phenyl)-acetamide |
| FT602 | 1.3010 | NS | 4.2701 | 2.0943 | 245.10 | 432.8102 |  | unindentified |
| FT607 | 1.0874 | NS | 0.241 | -2.0521 | 246.10 | 553.9181 | 469 | Pyrrolo[1,2-a]pyrazine-1,4-dione, hexahydro-3-(2-methylpropyl)- |
| FT610 | 1.3207 | NS | 5.3199 | 2.4114 | 250.10 | 433.8238 | 510 | Chromic acetylacetonate |
| FT611 | 1.4724 | 1.84E-02 | 2.1357 | 1.0947 | 251.09 | 482.0141 | 741 | β-D-Mannopyranoside, methyl 2,3,4,6-tetrakis-O-(trimethylsilyl)- |
| FT622 | 0.7921 | NS | 2.5484 | 1.3496 | 262.15 | 504.4969 | 498 | Phenylthioacetic acid, 4-cyanophenyl ester |
| FT639 | 0.0941 | NS | 2.5359 | 1.3425 | 271.09 | 403.7614 | 637 | Tyramine, trimethylsilyl ether |
| FT646 | 1.4317 | 2.70E-02 | 0.374 | -1.4185 | 275.10 | 432.9317 | 758 | Furan, 2,5-dihydro-2,5-dimethoxy- |
| FT653 | 0.8711 | NS | 0.211 | -2.2415 | 282.98 | 350.0357 |  | unindentified |
| FT676 | 1.0236 | NS | 0.453 | -1.1414 | 299.09 | 350.1687 |  | unindentified |
| FT678 | 1.0388 | NS | 0.456 | -1.1327 | 300.01 | 350.0307 |  | unindentified |
| FT679 | 0.9828 | NS | 0.470 | -1.0894 | 300.08 | 350.2787 |  | unindentified |
| FT681 | 0.9843 | NS | 0.487 | -1.037 | 301.07 | 350.0588 |  | unindentified |
| FT694 | 1.2314 | NS | 0.355 | -1.4919 | 313.23 | 504.0042 |  | unindentified |
| FT695 | 0.9788 | NS | 0.492 | -1.0235 | 314.09 | 350.0588 |  | unindentified |
| FT720 | 1.0809 | NS | 0.379 | -1.3997 | 337.20 | 532.0506 | 634 | Tetrasiloxane, 3,5-diethoxy-1,1,1,7,7,7-hexamethyl-3,5-bis(trimethylsiloxy)- |
| FT721 | 1.3478 | 4.97E-02 | 0.263 | -1.9261 | 337.26 | 531.9327 | 827 | Oleic acid, trimethylsilyl ester |
| FT723 | 1.3712 | 4.27E-02 | 0.271 | -1.8849 | 339.26 | 531.9411 | 730 | N-(4-Bromo-2,5-dimethylphenyl)-2-chlorobenzamide |
| FT727 | 0.7368 | NS | 0.341 | -1.551 | 341.15 | 530.7080 | 668 | 9,12-Octadecadienoic acid (Z,Z)-, trimethylsilyl ester |
| FT738 | 1.2263 | NS | 0.224 | -2.1561 | 361.20 | 485.4167 |  | unindentified |
| FT767 | 1.0166 | NS | 2.3485 | 1.2317 | 437.20 | 419.7287 | 598 | 4-Hydroxyphenylethanol, di-TMS |

**Table S6**. Differential metabolites in the uninoculated FORM15 culture medium after incubation for 12 h and 36 h at 180 rpm and 28 ºC. Peak ID indicates the peak number after XCMS analysis of the chromatograms. VIP score denotes the value of variable importance in projection, considering the component 1 of multivariate supervised discriminant analysis by partial least squares (sPLS-DA). The *p*-value indicates whether a significant difference (p < 0.05) or a non-significant difference (NS) is present according to Student's t-test. FC stands for fold-change analysis, which is the ratio between group means using data prior to column-wise normalization. Log2(FC) indicates the log2 transformed value for each comparison. Match Factor indicates the similarity score of a peak to the listed compounds in the NIST Mass Spectral Library (excellent match: >900; good match: 800–900; fair match: 700–800; medium to low confidence: <700).

| Peak ID | VIP score | *p*-value | FC | log2(FC) | *m/z* | RT (s) | Match Factor | Compounds |
| --- | --- | --- | --- | --- | --- | --- | --- | --- |
| FT002 | 1.4815 | NS | 2.1357 | 1.0947 | 44.99 | 327.0941 |  | unindentified |
| FT016 | 1.2511 | NS | 0.2955 | -1.7588 | 50.00 | 300.8238 | 807 | Acetamide, N-(2,6-dimethylphenyl)-N-(2-methylphenyl)- Acetamide, N-(2,6-dimethylphenyl)-N-(2-methylphenyl)- |
| FT031 | 1.4697 | NS | 2.3932 | 1.259 | 58.99 | 327.1555 | 678 | N-Cycloheptyl-2-iodo-benzamide |
| FT067 | 2.1231 | 1.06E-03 | 0.172 | -2.5354 | 73.05 | 442.9108 | 537 | Terephthalic acid, cyclobutyl decyl ester |
| FT070 | 1.6446 | NS | 3.7615 | 1.9113 | 73.05 | 327.1555 | 792 | 3,6,9-Trioxa-2,10-disilaundecane, 2,2,10,10-tetramethyl- |
| FT075 | 2.1242 | 1.02E-03 | 0.247 | -2.0164 | 74.05 | 442.9108 |  | unindentified |
| FT076 | 1.4344 | NS | 2.4528 | 1.2944 | 74.05 | 327.1555 | 556 | Pantoyl lactone, trimethylsilyl |
| FT112 | 1.8393 | 3.50E-02 | 2.8112 | 1.4912 | 87.04 | 486.0000 |  | unindentified |
| FT122 | 1.4643 | NS | 2.6193 | 1.3892 | 89.05 | 327.1555 |  | unindentified |
| FT136 | 1.2114 | NS | 3.6474 | 1.8669 | 91.09 | 484.6124 |  | unindentified |
| FT157 | 1.2503 | NS | 4.8097 | 2.2659 | 101.09 | 327.0030 | 411 | Butane, 1,2,3-tris(trimethylsiloxy)- |
| FT169 | 2.1346 | 6.88E-04 | 0.099 | -3.3283 | 103.10 | 442.4285 | 735 | Terephthalic acid, cyclobutyl decyl ester |
| FT170 | 1.3572 | NS | 2.141 | 1.0983 | 103.10 | 327.5430 |  | unindentified |
| FT181 | 1.8729 | 2.86E-02 | 0.429 | -1.2206 | 106.10 | 428.3851 | 347 | 2-Methylbenzyl p-toluate |
| FT182 | 1.3738 | NS | 0.469 | -1.0934 | 107.10 | 495.9923 | 766 | Tyrosine |
| FT200 | 1.0945 | NS | 2.0488 | 1.0348 | 113.10 | 348.0380 | 727 | 2(3H)-Furanone, 3-[(4-nitrophenyl)methylene]-5-phenyl- |
| FT213 | 1.2782 | NS | 3.5673 | 1.8348 | 116.10 | 327.0941 |  | unindentified |
| FT215 | 0.4314 | NS | 0.404 | -1.3086 | 116.10 | 333.8488 |  | unindentified |
| FT219 | 1.5668 | NS | 2.8835 | 1.5278 | 117.10 | 327.0327 |  | unindentified |
| FT232 | 0.9913 | NS | 0.462 | -1.1118 | 120.10 | 483.9204 | 618 | l-Alanine, N-(4-ethylbenzoyl)-, decyl ester |
| FT239 | 1.4004 | NS | 0.412 | -1.278 | 125.10 | 340.0211 | 816 | (Methoxymethyl)trimethylsilane |
| FT251 | 2.0235 | 7.67E-03 | 0.261 | -1.9372 | 129.09 | 441.9236 |  | unindentified |
| FT265 | 1.4017 | NS | 3.5913 | 1.8445 | 131.08 | 327.1555 | 735 | Pantoyl lactone, trimethylsilyl |
| FT269 | 1.2250 | NS | 2.1593 | 1.1106 | 132.06 | 348.0265 |  | unindentified |
| FT270 | 1.1695 | NS | 2.2778 | 1.1876 | 132.07 | 327.0941 |  | unindentified |
| FT278 | 1.2768 | NS | 3.7204 | 1.8955 | 133.05 | 327.0327 |  | unindentified |
| FT302 | 2.0055 | 9.50E-03 | 0.366 | -1.4487 | 143.07 | 440.8470 | 855 | Sucrose, octakis(trimethylsilyl) ether |
| FT320 | 2.1383 | 5.84E-04 | 0.170 | -2.5568 | 147.09 | 441.9144 | 735 | Terephthalic acid, cyclobutyl decyl ester |
| FT324 | 1.4741 | NS | 3.2736 | 1.7109 | 148.06 | 327.1555 |  | unindentified |
| FT325 | 1.9488 | 1.65E-02 | 0.447 | -1.162 | 148.09 | 440.8470 |  | unindentified |
| FT341 | 1.8634 | 3.03E-02 | 0.330 | -1.6003 | 153.08 | 346.0407 | 659 | Pyrrolo[1,2-a]pyrazine-1,4-dione, hexahydro-3-(phenylmethyl)- |
| FT346 | 1.2100 | NS | 0.261 | -1.9348 | 154.09 | 483.2593 | 682 | 7-Isoquinolinol, 1,2,3,4-tetrahydro-6-methoxy-2-methyl-1-[2-(3,4,5-trimethoxyphenyl)ethyl]- |
| FT355 | 2.0057 | 9.48E-03 | 0.310 | -1.6907 | 157.06 | 441.9107 |  | unindentified |
| FT378 | 1.3728 | NS | 0.436 | -1.1979 | 167.09 | 551.1802 |  | unindentified |
| FT382 | 2.1696 | 4.43E-05 | 0.053 | -4.2244 | 169.10 | 441.9044 |  | unindentified |
| FT383 | 1.7662 | NS | 0.331 | -1.5951 | 170.10 | 478.0498 | 682 | Glycyl-L-tyrosine |
| FT385 | 2.0613 | 4.47E-03 | 0.479 | -1.0626 | 170.10 | 485.3333 |  | unindentified |
| FT386 | 2.0308 | 6.99E-03 | 0.206 | -2.2782 | 170.10 | 442.4285 |  | unindentified |
| FT422 | 1.0337 | NS | 4.6836 | 2.2276 | 180.11 | 409.6467 | 701 | Tyramine, trimethylsilyl ether |
| FT435 | 2.1186 | 1.23E-03 | 0.240 | -2.0607 | 189.09 | 441.9160 |  | unindentified |
| FT441 | 2.1554 | 2.14E-04 | 0.193 | -2.3719 | 191.04 | 442.0627 |  | unindentified |
| FT445 | 2.1591 | 1.57E-04 | 0.173 | -2.5317 | 191.07 | 441.9353 |  | unindentified |
| FT468 | 1.7783 | 4.81E-02 | 0.252 | -1.9877 | 199.07 | 447.0001 | 676 | D-Turanose, heptakis(trimethylsilyl)- |
| FT486 | 1.1826 | NS | 2.2679 | 1.1814 | 204.10 | 348.0387 | 774 | Silane, trimethyl (propoxymethyl)- |
| FT490 | 1.4242 | NS | 5.9137 | 2.5641 | 205.10 | 327.1555 |  | unindentified |
| FT491 | 1.1750 | NS | 2.2948 | 1.1983 | 205.10 | 348.1303 |  | unindentified |
| FT521 | 2.1507 | 2.97E-04 | 0.038 | -4.7002 | 217.10 | 441.9198 |  | unindentified |
| FT526 | 1.1513 | NS | 2.599 | 1.378 | 217.10 | 348.0386 |  | unindentified |
| FT529 | 2.1335 | 7.19E-04 | 0.103 | -3.2817 | 218.09 | 441.9106 | 894 | α-D-Glucopyranoside, 1,3,4,6-tetrakis-O-(trimethylsilyl)-β-D-fructofuranosyl 2,3,4,6-tetrakis-O-(trimethylsilyl)- |
| FT534 | 2.1595 | 1.52E-04 | 0.207 | -2.2727 | 219.09 | 441.9198 |  | unindentified |
| FT539 | 1.2032 | NS | 2.1097 | 1.077 | 220.09 | 348.0265 |  | unindentified |
| FT561 | 1.7067 | NS | 0.220 | -2.1825 | 229.10 | 441.4873 |  | unindentified |
| FT563 | 1.3858 | NS | 0.096 | -3.3869 | 230.10 | 442.9854 | 717 | 3-Tetradecene, (Z)- |
| FT564 | 1.2862 | NS | 0.177 | -2.4989 | 230.10 | 432.6191 | 701 | Glucuronolactone, trisO-(trimethylsilyl)- |
| FT594 | 1.3668 | NS | 0.491 | -1.0274 | 243.09 | 432.4280 |  | unindentified |
| FT596 | 2.1500 | 3.10E-04 | 0.041 | -4.5919 | 243.10 | 441.9198 | 707 | Sucrose, octakis(trimethylsilyl) ether |
| FT598 | 1.1771 | NS | 0.407 | -1.2962 | 244.10 | 342.9062 | 877 | N-(3-Amino-2-hydroxy-phenyl)-acetamide |
| FT600 | 1.3486 | NS | 0.408 | -1.2926 | 244.10 | 339.0306 | 512 | 1,2-Dicarboxy-3-(4-chlorophenyl)-2,3(1H)-dihydropyrido(1,2-a)benzimidazole |
| FT601 | 1.9857 | 1.17E-02 | 0.110 | -3.1771 | 244.10 | 441.9053 |  | unindentified |
| FT602 | 1.8049 | 4.21E-02 | 0.336 | -1.5732 | 245.10 | 432.8102 |  | unindentified |
| FT604 | 1.2820 | NS | 0.151 | -2.7261 | 245.10 | 441.9198 |  | unindentified |
| FT610 | 1.1918 | NS | 0.375 | -1.4137 | 250.10 | 433.8238 | 510 | Chromic acetylacetonate |
| FT613 | 2.0811 | 3.13E-03 | 0.150 | -2.7364 | 257.09 | 441.9198 | 907 | Sucrose, octakis(trimethylsilyl) ether |
| FT622 | 1.1295 | NS | 3.4794 | 1.7988 | 262.15 | 504.4969 | 498 | Phenylthioacetic acid, 4-cyanophenyl ester |
| FT638 | 2.1668 | 6.78E-05 | 0.051 | -4.2788 | 271.10 | 441.9160 | 773 | Sucrose, octakis(trimethylsilyl) ether |
| FT639 | 1.2353 | NS | 2.1715 | 1.1187 | 271.09 | 403.7614 | 637 | Tyramine, trimethylsilyl ether |
| FT641 | 2.1259 | 9.64E-04 | 0.143 | -2.8098 | 272.10 | 441.9160 |  | unindentified |
| FT654 | 1.5759 | NS | 2.3899 | 1.2569 | 283.00 | 553.2478 | 493 | 4H-1-Benzopyran-4-one, 2,3-dihydro-6,7-dimethoxy-3-(2,3,4,5-tetramethoxyphenyl)-, (S)- |
| FT658 | 2.0670 | 4.05E-03 | 0.056 | -4.1604 | 289.10 | 447.0771 | 648 | Ribitol, 1,2,3,4,5-pentakis-O-(trimethylsilyl)- |
| FT662 | 1.9418 | 1.74E-02 | 0.069 | -3.8491 | 289.13 | 445.8668 | 634 | D-(+)-Trehalose, octakis(trimethylsilyl) ether |
| FT665 | 2.0602 | 4.55E-03 | 0.095 | -3.4012 | 290.10 | 446.8869 | 703 | 3,6-Dioxa-2,7-disilaoctane, 2,2,4,7,7-pentamethyl- |
| FT702 | 2.1275 | 9.14E-04 | 0.053 | -4.2365 | 319.10 | 442.0628 | 829 | α-D-Glucopyranoside, 1,3,4,6-tetrakis-O-(trimethylsilyl)-β-D-fructofuranosyl 2,3,4,6-tetrakis-O-(trimethylsilyl)- |
| FT704 | 2.1455 | 4.05E-04 | 0.058 | -4.1065 | 319.15 | 441.3573 | 907 | Sucrose, octakis(trimethylsilyl) ether |
| FT713 | 1.6285 | NS | 0.335 | -1.5789 | 331.11 | 441.9236 | 929 | α-D-Glucopyranoside, 1,3,4,6-tetrakis-O-(trimethylsilyl)-β-D-fructofuranosyl 2,3,4,6-tetrakis-O-(trimethylsilyl)- |
| FT736 | 1.8717 | 2.88E-02 | 0.022 | -5.4816 | 361.14 | 442.3656 |  | unindentified |
| FT737 | 2.1342 | 6.99E-04 | 0.011 | -6.5387 | 361.20 | 441.9044 |  | unindentified |
| FT739 | 1.1391 | NS | 0.238 | -2.0711 | 361.19 | 419.9162 | 889 | 4-Hydroxyphenylethanol, di-TMS |
| FT741 | 1.8020 | 4.31E-02 | 0.055 | -4.1844 | 362.11 | 442.2020 | 829 | α-D-Glucopyranoside, 1,3,4,6-tetrakis-O-(trimethylsilyl)-β-D-fructofuranosyl 2,3,4,6-tetrakis-O-(trimethylsilyl)- |
| FT742 | 1.7417 | NS | 0.137 | -2.8666 | 362.20 | 441.9036 |  | unindentified |
| FT744 | 2.1465 | 3.84E-04 | 0.039 | -4.6606 | 363.11 | 441.9236 | 603 | α-D-Glucopyranoside, 1,3,4,6-tetrakis-O-(trimethylsilyl)-β-D-fructofuranosyl 2,3,4,6-tetrakis-O-(trimethylsilyl)- |
| FT747 | 1.7673 | NS | 0.148 | -2.7529 | 363.20 | 441.9044 |  | unindentified |
| FT749 | 2.1605 | 1.38E-04 | 0.087 | -3.5148 | 364.17 | 441.1653 |  | unindentified |
| FT750 | 2.0090 | 9.13E-03 | 0.102 | -3.2891 | 365.15 | 446.9551 | 648 | Ribitol, 1,2,3,4,5-pentakis-O-(trimethylsilyl)- |
| FT764 | 2.1757 | 1.06E-05 | 0.049 | -4.3403 | 437.15 | 442.2019 | 764 | Sucrose, octakis(trimethylsilyl) ether |
| FT766 | 1.8161 | 4.07E-02 | 0.493 | -1.0204 | 437.20 | 441.3501 |  | unindentified |
| FT767 | 1.1177 | NS | 0.288 | -1.7972 | 437.20 | 419.7287 | 598 | 4-Hydroxyphenylethanol, di-TMS |
| FT768 | 2.0182 | 8.19E-03 | 0.138 | -2.8563 | 438.13 | 442.0627 |  | unindentified |
| FT770 | 2.1148 | 1.39E-03 | 0.172 | -2.5404 | 439.15 | 442.2019 |  | unindentified |
| FT771 | 1.5400 | NS | 0.363 | -1.4605 | 439.20 | 441.3501 | 707 | Sucrose, octakis(trimethylsilyl) ether |
| FT774 | 1.8523 | 3.23E-02 | 0.241 | -2.0502 | 451.18 | 441.9236 |  | unindentified |

**Table S7**. Differential metabolites in the uninoculated FORM15 culture medium after incubation for 36 h and 72 h at 180 rpm and 28 ºC. Peak ID indicates the peak number after XCMS analysis of the chromatograms. VIP score denotes the value of variable importance in projection, considering the component 1 of multivariate supervised discriminant analysis by partial least squares (sPLS-DA). The *p*-value indicates whether a significant difference (p < 0.05) or a non-significant difference (NS) is present according to Student's t-test. FC stands for fold-change analysis, which is the ratio between group means using data prior to column-wise normalization. Log2(FC) indicates the log2 transformed value for each comparison. Match Factor indicates the similarity score of a peak to the listed compounds in the NIST Mass Spectral Library (excellent match: >900; good match: 800–900; fair match: 700–800; medium to low confidence: <700).

| Peak ID | VIP score | *p*-value | FC | log2(FC) | *m/z* | RT (s) | Match Factor | Compounds |
| --- | --- | --- | --- | --- | --- | --- | --- | --- |
| FT002 | 0.8580 | NS | 0.287 | -1.8014 | 44.99 | 327.0941 |  | unindentified |
| FT012 | 0.9229 | NS | 0.319 | -1.6499 | 46.96 | 327.0941 |  | unindentified |
| FT031 | 0.9540 | NS | 0.241 | -2.0531 | 58.99 | 327.1555 | 678 | N-Cycloheptyl-2-iodo-benzamide |
| FT039 | 1.2944 | NS | 0.359 | -1.476 | 61.00 | 327.3492 | 692 | 3,6,9-Trioxa-2,10-disilaundecane, 2,2,10,10-tetramethyl- |
| FT051 | 1.4425 | NS | 2.071 | 1.0504 | 70.05 | 495.9961 |  | unindentified |
| FT064 | 1.1075 | NS | 3.4929 | 1.8044 | 72.14 | 486.0000 |  | unindentified |
| FT070 | 1.1416 | NS | 0.146 | -2.7797 | 73.05 | 327.1555 | 792 | 3,6,9-Trioxa-2,10-disilaundecane, 2,2,10,10-tetramethyl- |
| FT073 | 0.7006 | NS | 0.343 | -1.5445 | 73.05 | 333.8488 | 629 | Butane, 2-isocyanato- |
| FT076 | 1.0618 | NS | 0.215 | -2.2155 | 74.05 | 327.1555 | 556 | Pantoyl lactone, trimethylsilyl |
| FT081 | 0.5493 | NS | 0.377 | -1.4083 | 75.00 | 486.0000 |  | unindentified |
| FT082 | 0.7410 | NS | 0.399 | -1.3261 | 75.05 | 333.8488 |  | unindentified |
| FT099 | 1.8108 | 2.91E-02 | 3.6502 | 1.868 | 84.05 | 410.5290 |  | unindentified |
| FT107 | 1.7344 | 4.52E-02 | 5.8168 | 2.5402 | 86.10 | 322.1338 | 628 | tert-Butyl-[2-[2-(2-ethoxyethoxy)ethoxy]ethoxy]dimethylsilane |
| FT111 | 1.7933 | 3.25E-02 | 9.3224 | 3.2207 | 86.10 | 315.0363 | 743 | l-Leucine, trimethylsilyl ester |
| FT121 | 1.6652 | NS | 2.4631 | 1.3005 | 89.05 | 300.0390 |  | unindentified |
| FT122 | 0.5508 | NS | 0.231 | -2.1154 | 89.05 | 327.1555 |  | unindentified |
| FT139 | 1.3820 | NS | 0.416 | -1.2641 | 92.09 | 354.7446 | 748 | Benzeneethanamine |
| FT140 | 1.4988 | NS | 2.4031 | 1.2649 | 92.10 | 339.6141 | 571 | Ergotaman-3',6',18-trione, 9,10-dihydro-12'-hydroxy-2'-methyl-5'-(phenylmethyl)-, (5'α,10α)- |
| FT141 | 1.0429 | NS | 2.838 | 1.5049 | 93.02 | 300.9320 | 807 | Acetamide, N-(2,6-dimethylphenyl)-N-(2-methylphenyl)- |
| FT149 | 1.9722 | 6.47E-03 | 0.368 | -1.441 | 99.10 | 422.0801 | 645 | 4-Amino-4,5(1H)-dihydro-1,2,4-triazole-5-one |
| FT157 | 1.0742 | NS | 0.103 | -3.2737 | 101.09 | 327.0030 | 411 | Butane, 1,2,3-tris(trimethylsiloxy)- |
| FT170 | 1.2558 | NS | 0.187 | -2.4207 | 103.10 | 327.5430 |  | unindentified |
| FT172 | 1.4752 | NS | 2.176 | 1.1217 | 104.10 | 486.0000 |  | unindentified |
| FT190 | 0.7305 | NS | 2.1005 | 1.0707 | 108.09 | 417.0645 |  | unindentified |
| FT205 | 1.1527 | NS | 2.1202 | 1.0842 | 114.08 | 449.1627 | 598 | 3-Methyl-4-amino-4,5(1H)-dihydro-1,2,4-triazole-5-one |
| FT213 | 0.9264 | NS | 0.146 | -2.7701 | 116.10 | 327.0941 |  | unindentified |
| FT215 | 0.9767 | NS | 0.208 | -2.264 | 116.10 | 333.8488 |  | unindentified |
| FT219 | 0.5621 | NS | 0.229 | -2.1264 | 117.10 | 327.0327 |  | unindentified |
| FT224 | 0.7458 | NS | 0.268 | -1.8971 | 118.10 | 326.9425 | 698 | 3,6,9-Trioxa-2,10-disilaundecane, 2,2,10,10-tetramethyl- |
| FT227 | 1.5139 | NS | 2.3145 | 1.2107 | 119.10 | 486.0000 |  | unindentified |
| FT233 | 0.7727 | NS | 0.370 | -1.4342 | 121.10 | 486.3158 | 630 | Ethyl 2,3,4,6-tetrakis-O-(trimethylsilyl)-D-glucopyranoside |
| FT258 | 1.4847 | NS | 2.6974 | 1.4316 | 130.09 | 347.9841 | 948 | Glycerol, tris(trimethylsilyl) ether |
| FT265 | 0.7778 | NS | 0.145 | -2.7871 | 131.08 | 327.1555 | 735 | Pantoyl lactone, trimethylsilyl |
| FT270 | 0.7664 | NS | 0.293 | -1.7721 | 132.07 | 327.0941 |  | unindentified |
| FT278 | 1.2629 | NS | 0.141 | -2.8264 | 133.05 | 327.03273 |  | unindentified |
| FT304 | 1.3449 | NS | 2.1472 | 1.1025 | 143.08 | 507.0563 |  | unindentified |
| FT316 | 0.8249 | NS | 0.236 | -2.0859 | 147.05 | 333.8488 |  | unindentified |
| FT324 | 1.5061 | NS | 0.167 | -2.5782 | 148.06 | 327.1555 |  | unindentified |
| FT333 | 1.2045 | NS | 2.6598 | 1.4113 | 149.10 | 332.0562 | 716 | 2-Ethyl-2-phenyl-1,3-benzodioxole |
| FT368 | 1.2881 | NS | 0.186 | -2.4239 | 164.07 | 486.9657 | 641 | Di-N-propylethylamine |
| FT371 | 1.0418 | NS | 2.0339 | 1.0243 | 165.10 | 406.9612 | 598 | 2,3,4,5-Tetrahydropyridazine |
| FT376 | 1.5233 | NS | 2.8073 | 1.4892 | 166.10 | 502.0041 |  | unindentified |
| FT380 | 1.2564 | NS | 2.0757 | 1.0536 | 168.10 | 458.0905 | 544 | 3-Ethoxy-4-methoxyphenol |
| FT421 | 1.5952 | NS | 2.1938 | 1.1335 | 180.10 | 420.8306 | 895 | 4-Hydroxyphenylethanol, di-TMS |
| FT450 | 1.4401 | NS | 2.622 | 1.3906 | 192.97 | 349.9706 | 908 | Silanol, trimethyl-, phosphate (3:1) |
| FT490 | 0.9910 | NS | 0.079 | -3.6574 | 205.10 | 327.1555 |  | unindentified |
| FT508 | 1.7819 | 3.48E-02 | 3.3036 | 1.724 | 210.97 | 350.0251 |  | unindentified |
| FT520 | 1.8335 | 2.50E-02 | 2.7156 | 1.4413 | 217.10 | 457.2807 | 720 | Silane, diethylethoxyoctyloxy- |
| FT524 | 1.0403 | NS | 0.494 | -1.0158 | 217.09 | 418.0645 | 788 | Sucrose, octakis (trimethylsilyl) ether |
| FT527 | 1.1095 | NS | 0.337 | -1.5682 | 217.10 | 432.6191 |  | unindentified |
| FT554 | 1.4248 | NS | 2.1142 | 1.0801 | 225.05 | 350.0357 |  | unindentified |
| FT564 | 1.4282 | NS | 0.190 | -2.3975 | 230.10 | 432.6191 | 701 | Glucuronolactone, trisO-(trimethylsilyl)- |
| FT570 | 1.4221 | NS | 2.5436 | 1.3469 | 231.10 | 485.5272 |  | unindentified |
| FT594 | 1.1543 | NS | 0.370 | -1.4329 | 243.09 | 432.4280 |  | unindentified |
| FT602 | 1.4067 | NS | 0.244 | -2.0336 | 245.10 | 432.8102 |  | unindentified |
| FT610 | 1.5102 | NS | 0.217 | -2.201 | 250.10 | 433.8238 | 510 | Chromic acetylacetonate |
| FT622 | 0.1116 | NS | 0.464 | -1.1061 | 262.15 | 504.4969 | 498 | Phenylthioacetic acid, 4-cyanophenyl ester |
| FT653 | 0.6488 | NS | 2.8019 | 1.4864 | 282.98 | 350.0357 |  | unindentified |
| FT676 | 1.2800 | NS | 3.1806 | 1.6693 | 299.09 | 350.1687 |  | unindentified |
| FT678 | 1.2794 | NS | 2.7072 | 1.4368 | 300.01 | 350.0307 |  | unindentified |
| FT679 | 1.2971 | NS | 2.8773 | 1.5247 | 300.08 | 350.2787 |  | unindentified |
| FT680 | 1.2003 | NS | 2.3644 | 1.2415 | 301.03 | 350.1094 |  | unindentified |
| FT681 | 1.3515 | NS | 2.6686 | 1.4161 | 301.07 | 350.0588 |  | unindentified |
| FT695 | 1.3980 | NS | 2.9048 | 1.5384 | 314.09 | 350.0588 |  | unindentified |
| FT712 | 1.6824 | NS | 2.4229 | 1.2768 | 331.14 | 485.8571 | 810 | Mannose, 6-deoxy-2,3,4,5-tetrakis-O-(trimethylsilyl)-, L- |
| FT734 | 1.2496 | NS | 2.5008 | 1.3224 | 361.10 | 485.3125 | 564 | D-(+)-Trehalose, octakis(trimethylsilyl) ether |
| FT735 | 0.9324 | NS | 0.340 | -1.5565 | 361.13 | 420.0736 | 573 | 2-Amino-1-phenylethanol, trimethylsilyl ether |
| FT740 | 1.6193 | NS | 2.0476 | 1.0339 | 362.13 | 485.3125 |  | unindentified |

**Table S8**. Differential metabolites in the FORM15 culture medium inoculated with *Azospirillum brasilense* Ab-V5 after incubation for 12 h and 36 h at 180 rpm and 28 ºC. Peak ID indicates the peak number after XCMS analysis of the chromatograms. VIP score denotes the value of variable importance in projection, considering the component 1 of multivariate supervised discriminant analysis by partial least squares (sPLS-DA). The *p*-value indicates whether a significant difference (p < 0.05) or a non-significant difference (NS) is present according to Student's t-test. FC stands for fold-change analysis, which is the ratio between group means using data prior to column-wise normalization. Log2(FC) indicates the log2 transformed value for each comparison. Match Factor indicates the similarity score of a peak to the listed compounds in the NIST Mass Spectral Library (excellent match: >900; good match: 800–900; fair match: 700–800; medium to low confidence: <700).

| Peak ID | VIP score | *p*-value | FC | log2(FC) | *m/z* | RT (s) | Match Factor | Compounds |
| --- | --- | --- | --- | --- | --- | --- | --- | --- |
| FT003 | 1.3035 | NS | 2.7155 | 1.4412 | 44.95 | 359.2051 | 702 | Sym-tetramethyl (diisopropyl)disiloxane |
| FT008 | 1.2173 | NS | 2.2174 | 1.1489 | 45.99 | 348.0509 | 808 | Glycerol, tris(trimethylsilyl) ether |
| FT010 | 1.1928 | NS | 2.1099 | 1.0772 | 46.95 | 348.0387 | 950 | Glycerol, tris(trimethylsilyl) ether |
| FT015 | 1.5360 | 1.83E-02 | 2.5538 | 1.3526 | 49.96 | 300.2195 |  | unindentified |
| FT020 | 1.4911 | 2.74E-02 | 3.3274 | 1.7344 | 56.02 | 326.1130 | 781 | N-methyliminopropylbenzene |
| FT022 | 1.6855 | 1.02E-03 | 0.337 | -1.5675 | 56.05 | 486.5014 | 576 | Methylphosphonic acid, fluoroanhydride, 3-methyl-1-(2-methylpropyl)butyl ester |
| FT023 | 1.6517 | 3.09E-03 | 0.376 | -1.412 | 56.05 | 427.0527 | 372 | 1H-Isoindole-1,3(2H)-dione, 2-(7-azabicyclo[2.2.1]hept-1-en-7-yl)- |
| FT026 | 0.9628 | NS | 0.428 | -1.2235 | 57.05 | 390.3981 | 513 | 1H-Tetrazol-5-amine |
| FT027 | 1.1911 | NS | 2.6064 | 1.3821 | 58.01 | 348.0504 | 768 | 2-Phenylamino-6,7-dihydro-5H-1-thia-8-aza-S-indacene-3-carbaldehyde |
| FT031 | 1.0707 | NS | 2.1819 | 1.1256 | 58.99 | 327.1555 | 678 | N-Cycloheptyl-2-iodo-benzamide |
| FT036 | 1.2436 | NS | 2.6757 | 1.4199 | 60.00 | 348.0504 | 750 | Glycerol, tris(trimethylsilyl) ether |
| FT042 | 1.3282 | NS | 2.9808 | 1.5757 | 63.00 | 300.1271 | 931 | Benzeneethanamine |
| FT044 | 1.5761 | 1.16E-02 | 2.9238 | 1.5478 | 64.02 | 300.2253 | 634 | Isothiazole, 3,5-bis(methylthio)-4-phenyl- |
| FT045 | 1.2733 | NS | 2.4893 | 1.3157 | 65.00 | 300.0397 |  | unindentified |
| FT063 | 1.0884 | NS | 21.737 | 4.4421 | 72.15 | 348.1051 | 924 | Glycerol, tris(trimethylsilyl) ether |
| FT065 | 1.0945 | NS | 2.0403 | 1.0288 | 73.05 | 348.0756 |  | unindentified |
| FT067 | 1.6016 | 8.16E-03 | 0.180 | -2.4765 | 73.05 | 442.9108 | 537 | Terephthalic acid, cyclobutyl decyl ester |
| FT073 | 1.2351 | NS | 0.068 | -3.8828 | 73.05 | 333.8488 | 629 | Butane, 2-isocyanato- |
| FT074 | 1.1821 | NS | 2.0946 | 1.0667 | 74.05 | 348.1003 |  | unindentified |
| FT075 | 1.5648 | 1.34E-02 | 0.253 | -1.981 | 74.05 | 442.9108 |  | unindentified |
| FT076 | 1.0129 | NS | 2.3427 | 1.2282 | 74.05 | 327.1555 | 556 | Pantoyl lactone, trimethylsilyl |
| FT082 | 1.2995 | NS | 0.105 | -3.256 | 75.05 | 333.8488 |  | unindentified |
| FT083 | 1.1462 | NS | 2.3758 | 1.2484 | 75.04 | 348.0509 |  | unindentified |
| FT084 | 0.9695 | NS | 3.4152 | 1.772 | 75.02 | 532.0743 | 679 | Octadecanoic acid, trimethylsilyl ester |
| FT090 | 1.3058 | NS | 2.4964 | 1.3199 | 76.03 | 531.9411 | 812 | 9,12-Octadecadienoic acid (Z,Z)-, trimethylsilyl ester |
| FT093 | 1.2461 | NS | 2.6416 | 1.4014 | 77.01 | 417.0645 | 697 | Phenol, 4-(2-aminoethyl)- |
| FT103 | 1.1697 | NS | 2.176 | 1.1217 | 85.05 | 348.1603 |  | unindentified |
| FT106 | 0.8358 | NS | 0.417 | -1.2617 | 85.05 | 390.3981 | 607 | Pentanethioic acid, S-ethyl ester |
| FT107 | 1.2553 | NS | 3.0705 | 1.6185 | 86.10 | 322.1338 | 628 | tert-Butyl-[2-[2-(2-ethoxyethoxy)ethoxy]ethoxy]dimethylsilane |
| FT110 | 1.2500 | NS | 2.5869 | 1.3712 | 86.10 | 423.2486 | 672 | 2-Chloro-3,3,4,4-tetrafluoro-1,2-oxazetidine |
| FT111 | 1.1167 | NS | 2.1857 | 1.1281 | 86.10 | 315.0363 | 743 | l-Leucine, trimethylsilyl ester |
| FT115 | 1.1455 | NS | 2.5576 | 1.3548 | 88.05 | 348.0498 |  | unindentified |
| FT116 | 1.2419 | NS | 0.311 | -1.6865 | 88.10 | 486.0066 | 802 | Gulose, 2,3,4,5,6-pentakis-O-(trimethylsilyl)- |
| FT117 | 1.6780 | 1.38E-03 | 0.232 | -2.1095 | 88.10 | 426.7490 | 761 | N-(2-Methoxyphenyl)-2-nitrobenzenecarbothioamide |
| FT122 | 0.9711 | NS | 2.3021 | 1.203 | 89.05 | 327.1555 |  | unindentified |
| FT125 | 1.1762 | NS | 2.1883 | 1.1298 | 90.05 | 326.0347 | 447 | 3,6,9-Trioxa-2,10-disilaundecane, 2,2,10,10-tetramethyl- |
| FT135 | 1.6480 | 3.38E-03 | 0.051 | -4.2801 | 91.10 | 353.8015 | 794 | Benzeneethanamine |
| FT136 | 0.8071 | NS | 2.3091 | 1.2074 | 91.09 | 484.6124 |  | unindentified |
| FT137 | 1.3545 | NS | 51.468 | 5.6856 | 91.10 | 300.0405 |  | unindentified |
| FT139 | 1.3520 | NS | 0.258 | -1.9523 | 92.09 | 354.7446 | 748 | Benzeneethanamine |
| FT140 | 1.0679 | NS | 2.0349 | 1.0249 | 92.10 | 339.6141 | 571 | Ergotaman-3',6',18-trione, 9,10-dihydro-12'-hydroxy-2'-methyl-5'-(phenylmethyl)-, (5'α,10α)- |
| FT141 | 1.2142 | NS | 3.2002 | 1.6781 | 93.02 | 300.9320 | 807 | Acetamide, N-(2,6-dimethylphenyl)-N-(2-methylphenyl)- |
| FT151 | 0.8023 | NS | 0.221 | -2.1774 | 100.08 | 422.2989 | 948 | 4-Amino-4,5(1H)-dihydro-1,2,4-triazole-5-one |
| FT152 | 1.1101 | NS | 0.157 | -2.6691 | 100.10 | 429.7755 |  | unindentified |
| FT154 | 1.5422 | 1.72E-02 | 0.099 | -3.3357 | 100.10 | 487.0781 | 837 | Ethyl 2,3,4,6-tetrakis-O-(trimethylsilyl)-D-glucopyranoside |
| FT157 | 0.4773 | NS | 2.0435 | 1.0311 | 101.09 | 327.0030 | 411 | Butane, 1,2,3-tris(trimethylsiloxy)- |
| FT160 | 0.7912 | NS | 0.359 | -1.4772 | 101.10 | 429.7755 | 347 | 2-Methylbenzyl p-toluate |
| FT165 | 1.1523 | NS | 2.3781 | 1.2498 | 102.14 | 348.0498 |  | unindentified |
| FT169 | 1.6762 | 1.48E-03 | 0.101 | -3.3006 | 103.10 | 442.4285 | 735 | Terephthalic acid, cyclobutyl decyl ester |
| FT175 | 0.9658 | NS | 4.9506 | 2.3076 | 105.05 | 321.1933 | 567 | tert-Butyl-[2-[2-(2-ethoxyethoxy)ethoxy]ethoxy]dimethylsilane |
| FT178 | 1.7021 | 4.13E-04 | 0.107 | -3.2295 | 105.10 | 428.7658 | 861 | N-(2-Methoxyphenyl)-2-nitrobenzenecarbothioamide |
| FT181 | 1.6460 | 3.55E-03 | 0.244 | -2.0341 | 106.10 | 428.3851 | 347 | 2-Methylbenzyl p-toluate |
| FT184 | 1.3156 | NS | 3.3605 | 1.7487 | 107.09 | 417.0645 | 697 | Phenol, 4-(2-aminoethyl)- |
| FT186 | 1.6686 | 1.92E-03 | 0.424 | -1.2366 | 107.10 | 478.9640 | 682 | Glycyl-L-tyrosine |
| FT187 | 1.2025 | NS | 0.459 | -1.1223 | 107.10 | 486.4315 | 630 | Ethyl 2,3,4,6-tetrakis-O-(trimethylsilyl)-D-glucopyranoside |
| FT189 | 1.3459 | NS | 2.2094 | 1.1437 | 108.10 | 407.0951 |  | unindentified |
| FT190 | 1.3682 | NS | 4.9134 | 2.2967 | 108.09 | 417.0645 |  | unindentified |
| FT195 | 1.3148 | NS | 0.398 | -1.3299 | 111.10 | 485.9875 | 530 | Ethyl 2,3,4,6-tetrakis-O-(trimethylsilyl)-D-glucopyranoside |
| FT198 | 1.1455 | NS | 2.4821 | 1.3116 | 113.05 | 348.0509 |  | unindentified |
| FT210 | 1.1855 | NS | 2.4941 | 1.3185 | 115.06 | 348.0265 |  | unindentified |
| FT213 | 1.0175 | NS | 2.6851 | 1.425 | 116.10 | 327.0941 |  | unindentified |
| FT215 | 1.2176 | NS | 0.015 | -6.0268 | 116.10 | 333.8488 |  | unindentified |
| FT216 | 1.1462 | NS | 2.7135 | 1.4402 | 116.14 | 348.1056 | 934 | Silanol, trimethyl-, phosphate (3:1) |
| FT217 | 1.0847 | NS | 2.0393 | 1.0281 | 117.10 | 348.0756 |  | unindentified |
| FT219 | 0.8912 | NS | 2.1442 | 1.1004 | 117.10 | 327.0327 |  | unindentified |
| FT220 | 0.2776 | NS | 2.8867 | 1.5294 | 117.10 | 531.9411 | 371 | N-Cyclohexyl-1-pyrrolidinecarbothioamide |
| FT225 | 1.0841 | NS | 2.5557 | 1.3537 | 118.10 | 348.0262 |  | unindentified |
| FT226 | 1.1942 | NS | 2.307 | 1.206 | 119.06 | 348.1003 |  | unindentified |
| FT232 | 1.7177 | 4.37E-05 | 0.174 | -2.5246 | 120.10 | 483.9204 | 618 | l-Alanine, N-(4-ethylbenzoyl)-, decyl ester |
| FT233 | 1.6990 | 5.03E-04 | 0.151 | -2.7307 | 121.10 | 486.3158 | 630 | Ethyl 2,3,4,6-tetrakis-O-(trimethylsilyl)-D-glucopyranoside |
| FT239 | 1.0298 | NS | 0.489 | -1.0314 | 125.10 | 340.0211 | 816 | (Methoxymethyl)trimethylsilane |
| FT245 | 1.1180 | NS | 2.955 | 1.5632 | 127.10 | 553.2478 | 609 | 3,6-Dibutyl-1,2-dihydro-1,2,4,5-tetrazine |
| FT250 | 1.2066 | NS | 2.885 | 1.5286 | 129.10 | 348.0504 | 750 | Glycerol, tris(trimethylsilyl) ether |
| FT251 | 1.6471 | 3.45E-03 | 0.195 | -2.3556 | 129.09 | 441.9236 |  | unindentified |
| FT252 | 1.0747 | NS | 2.5732 | 1.3636 | 129.10 | 532.0743 |  | unindentified |
| FT254 | 1.3034 | NS | 0.212 | -2.238 | 129.11 | 429.7755 | 661 | N-(2-Methoxyphenyl)-2-nitrobenzenecarbothioamide # |
| FT257 | 0.9517 | NS | 0.431 | -1.2131 | 130.10 | 514.9975 | 363 | Myo-Inositol, 1,2,3,4,5,6-hexakis-O-(trimethylsilyl)- |
| FT258 | 1.1905 | NS | 2.3804 | 1.2512 | 130.09 | 347.9841 | 948 | Glycerol, tris(trimethylsilyl) ether |
| FT259 | 1.4188 | 4.58E-02 | 0.377 | -1.4058 | 130.10 | 469.3359 | 703 | Alanine, N-methyl-N-ethoxycarbonyl-, dodecyl ester |
| FT260 | 1.4705 | 3.22E-02 | 0.425 | -1.2329 | 130.09 | 425.8345 | 828 | Benzenepropanoic acid, α-[(trimethylsilyl)oxy]-, trimethylsilyl ester |
| FT262 | 1.2513 | NS | 2.9259 | 1.5489 | 131.04 | 348.0509 |  | unindentified |
| FT270 | 0.9949 | NS | 2.2601 | 1.1764 | 132.07 | 327.0941 |  | unindentified |
| FT274 | 1.1739 | NS | 2.175 | 1.121 | 132.10 | 532.9350 | 740 | Octadecanoic acid, trimethylsilyl ester |
| FT275 | 1.0922 | NS | 2.1694 | 1.1173 | 132.13 | 347.9841 | 790 | Glycerol, tris(trimethylsilyl) ether |
| FT276 | 1.0832 | NS | 2.0057 | 1.0041 | 133.05 | 348.0756 |  | unindentified |
| FT281 | 1.1996 | NS | 2.8039 | 1.4875 | 134.05 | 348.0509 |  | unindentified |
| FT289 | 1.2094 | NS | 2.2104 | 1.1443 | 137.10 | 417.0645 |  | unindentified |
| FT302 | 1.4401 | 3.99E-02 | 0.338 | -1.5654 | 143.07 | 440.8470 | 855 | Sucrose, octakis(trimethylsilyl) ether |
| FT305 | 1.4125 | 4.76E-02 | 2.6545 | 1.4084 | 143.10 | 328.1278 | 603 | Butane, 1,2,3-tris(trimethylsiloxy)- |
| FT309 | 1.0483 | NS | 2.2506 | 1.1703 | 145.10 | 532.0743 | 371 | N-Cyclohexyl-1-pyrrolidinecarbothioamide |
| FT312 | 1.3598 | NS | 2.8042 | 1.4876 | 145.10 | 318.0349 | 596 | 3-Methyl-1,2-bis(trimethylsilyl)butane |
| FT314 | 1.2276 | NS | 2.6377 | 1.3993 | 146.14 | 348.0498 |  | unindentified |
| FT316 | 1.2512 | NS | 0.041 | -4.6109 | 147.05 | 333.8488 |  | unindentified |
| FT317 | 1.0893 | NS | 2.0626 | 1.0444 | 147.10 | 348.1003 |  | unindentified |
| FT320 | 1.6017 | 8.14E-03 | 0.169 | -2.5664 | 147.09 | 441.9144 | 735 | Terephthalic acid, cyclobutyl decyl ester |
| FT324 | 0.7232 | NS | 2.0066 | 1.0047 | 148.06 | 327.1555 |  | unindentified |
| FT325 | 1.4659 | 3.34E-02 | 0.336 | -1.574 | 148.09 | 440.8470 |  | unindentified |
| FT328 | 1.7200 | 5.94E-05 | 0.162 | -2.6265 | 148.10 | 426.9009 | 766 | Benzenepropanoic acid, α-[(trimethylsilyl)oxy]-, trimethylsilyl ester |
| FT333 | 1.2151 | NS | 0.276 | -1.8565 | 149.10 | 332.0562 | 716 | 2-Ethyl-2-phenyl-1,3-benzodioxole |
| FT334 | 1.1208 | NS | 2.2394 | 1.1631 | 150.07 | 348.0504 |  | unindentified |
| FT341 | 1.0414 | NS | 0.455 | -1.1368 | 153.08 | 346.0407 | 659 | Pyrrolo[1,2-a]pyrazine-1,4-dione, hexahydro-3-(phenylmethyl)- |
| FT346 | 1.6737 | 2.14E-03 | 0.283 | -1.8211 | 154.09 | 483.2593 | 682 | 7-Isoquinolinol, 1,2,3,4-tetrahydro-6-methoxy-2-methyl-1-[2-(3,4,5-trimethoxyphenyl)ethyl]- |
| FT355 | 1.5436 | 1.69E-02 | 0.287 | -1.8008 | 157.06 | 441.9106 |  | unindentified |
| FT357 | 1.3207 | NS | 2.6551 | 1.4088 | 159.05 | 348.0262 | 892 | 1-(1-Adamantyl)-2-triisopropylsilyloxyethane |
| FT365 | 1.2284 | NS | 2.5653 | 1.3591 | 163.05 | 348.0382 |  | unindentified |
| FT368 | 1.5471 | 1.63E-02 | 0.148 | -2.7522 | 164.07 | 486.9657 | 641 | Di-N-propylethylamine |
| FT371 | 1.3302 | NS | 3.9692 | 1.9888 | 165.10 | 406.9612 | 598 | 2,3,4,5-Tetrahydropyridazine |
| FT373 | 1.5281 | 1.98E-02 | 4.0206 | 2.0074 | 166.05 | 327.0345 | 799 | Pantoyl lactone, trimethylsilyl |
| FT382 | 1.4883 | 2.81E-02 | 0.103 | -3.2791 | 169.10 | 441.9044 |  | unindentified |
| FT383 | 1.3682 | NS | 0.357 | -1.4873 | 170.10 | 478.0498 | 682 | Glycyl-L-tyrosine |
| FT386 | 1.6067 | 7.53E-03 | 0.219 | -2.1909 | 170.10 | 442.4285 |  | unindentified |
| FT396 | 1.1895 | NS | 2.4691 | 1.304 | 175.05 | 348.0504 |  | unindentified |
| FT403 | 1.2216 | NS | 2.9951 | 1.5826 | 177.05 | 348.0387 |  | unindentified |
| FT407 | 1.2019 | NS | 2.0765 | 1.0542 | 178.06 | 348.0382 |  | unindentified |
| FT411 | 1.0233 | NS | 0.133 | -2.9093 | 179.03 | 347.5509 |  | unindentified |
| FT412 | 1.1749 | NS | 2.3991 | 1.2625 | 179.05 | 344.0099 | 776 | Benzeneethanamine |
| FT414 | 1.1175 | NS | 2.1344 | 1.0938 | 179.10 | 406.0970 |  | unindentified |
| FT420 | 1.4686 | 3.27E-02 | 6.6262 | 2.7282 | 180.10 | 407.4287 | 717 | Silane, dimethyl-2-propenyl(tetradecyloxy)- |
| FT421 | 1.0829 | NS | 2.0525 | 1.0374 | 180.10 | 420.8306 | 895 | 4-Hydroxyphenylethanol, di-TMS |
| FT422 | 1.6573 | 2.67E-03 | 5.9382 | 2.570 | 180.11 | 409.6467 | 701 | Tyramine, trimethylsilyl ether |
| FT425 | 1.2217 | NS | 2.4305 | 1.2813 | 181.10 | 407.4287 |  | unindentified |
| FT433 | 1.1587 | NS | 2.2325 | 1.1587 | 189.05 | 348.0264 |  | unindentified |
| FT435 | 1.5721 | 1.22E-02 | 0.194 | -2.3662 | 189.09 | 441.9160 |  | unindentified |
| FT441 | 1.4306 | 4.25E-02 | 0.287 | -1.8025 | 191.04 | 442.0627 |  | unindentified |
| FT443 | 1.0774 | NS | 2.0114 | 1.0082 | 191.05 | 348.0756 |  | unindentified |
| FT445 | 1.6047 | 7.77E-03 | 0.153 | -2.7098 | 191.07 | 441.9353 |  | unindentified |
| FT446 | 1.1687 | NS | 2.7999 | 1.4854 | 191.09 | 348.1984 |  | unindentified |
| FT450 | 1.1120 | NS | 2.7213 | 1.4443 | 192.97 | 349.9706 | 908 | Silanol, trimethyl-, phosphate (3:1) |
| FT477 | 1.1066 | NS | 2.7625 | 1.466 | 203.09 | 348.0509 | 529 | Silanol, trimethyl-, phosphate (3:1) |
| FT483 | 1.2394 | NS | 3.5394 | 1.8235 | 203.10 | 348.1618 | 757 | Glycerol, tris(trimethylsilyl) ether |
| FT484 | 0.6847 | NS | 3.9192 | 1.9706 | 204.09 | 509.0190 | 525 | Phenylthioacetic acid, 4-cyanophenyl ester |
| FT486 | 1.2323 | NS | 3.4517 | 1.7873 | 204.10 | 348.0387 | 774 | Silane, trimethyl (propoxymethyl)- |
| FT487 | 0.6806 | NS | 3.9039 | 1.9649 | 204.11 | 508.7986 | 751 | Gulose, 2,3,4,5,6-pentakis-O-(trimethylsilyl)- |
| FT488 | 1.2320 | NS | 3.3811 | 1.7575 | 204.13 | 348.3706 | 757 | Glycerol, tris(trimethylsilyl) ether |
| FT490 | 0.9260 | NS | 3.8755 | 1.9544 | 205.10 | 327.1555 |  | unindentified |
| FT491 | 1.1046 | NS | 2.1454 | 1.1012 | 205.10 | 348.1303 |  | unindentified |
| FT493 | 1.0774 | NS | 2.0765 | 1.0541 | 206.10 | 348.1303 | 551 | 2-Octen-1-ol |
| FT501 | 1.2175 | NS | 3.0968 | 1.6308 | 207.09 | 348.0264 |  | unindentified |
| FT503 | 1.1053 | NS | 2.1248 | 1.0873 | 208.08 | 348.0504 |  | unindentified |
| FT520 | 1.4295 | 4.28E-02 | 3.0203 | 1.5947 | 217.10 | 457.2807 | 720 | Silane, diethylethoxyoctyloxy- |
| FT521 | 1.5117 | 2.30E-02 | 0.087 | -3.5183 | 217.10 | 441.9198 |  | unindentified |
| FT523 | 0.1892 | NS | 0.481 | -1.0551 | 217.09 | 404.6749 | 531 | Benzeneethanamine, N-[(pentafluorophenyl)methylene]-4-[(trimethylsilyl)oxy]- |
| FT524 | 1.2393 | NS | 0.446 | -1.1661 | 217.09 | 418.0645 | 788 | ose, octakis (trimethylsilyl) ether |
| FT526 | 1.2261 | NS | 3.226 | 1.6898 | 217.10 | 348.0386 |  | unindentified |
| FT527 | 1.4076 | 4.91E-02 | 0.211 | -2.2409 | 217.10 | 432.6191 |  | unindentified |
| FT529 | 1.5761 | 1.16E-02 | 0.130 | -2.9477 | 218.09 | 441.9106 | 894 | α-D-Glucopyranoside, 1,3,4,6-tetrakis-O-(trimethylsilyl)-β-D-fructofuranosyl 2,3,4,6-tetrakis-O-(trimethylsilyl)- |
| FT531 | 1.0921 | NS | 2.1832 | 1.1264 | 218.10 | 348.0756 |  | unindentified |
| FT534 | 1.6750 | 1.54E-03 | 0.175 | -2.5117 | 219.09 | 441.9198 |  | unindentified |
| FT538 | 1.2297 | NS | 3.3454 | 1.7422 | 219.13 | 348.0498 |  | unindentified |
| FT544 | 1.1642 | NS | 2.2043 | 1.1403 | 221.08 | 348.0380 |  | unindentified |
| FT550 | 1.3770 | NS | 2.2257 | 1.1542 | 223.10 | 458.9928 | 518 | 1,4-Dioxaspiro[4.5]decane, 8-chloro- |
| FT561 | 1.5803 | 1.10E-02 | 0.295 | -1.759 | 229.10 | 441.4873 |  | unindentified |
| FT563 | 1.6107 | 7.06E-03 | 0.146 | -2.7787 | 230.10 | 442.9854 | 717 | 3-Tetradecene, (Z)- |
| FT564 | 1.5902 | 9.93E-03 | 0.041 | -4.6001 | 230.10 | 432.6191 | 701 | Glucuronolactone, trisO-(trimethylsilyl)- |
| FT594 | 1.2803 | NS | 0.259 | -1.9512 | 243.09 | 432.4280 |  | unindentified |
| FT596 | 1.4649 | 3.38E-02 | 0.180 | -2.4728 | 243.10 | 441.9198 | 707 | Sucrose, octakis(trimethylsilyl) ether |
| FT598 | 1.3374 | NS | 0.306 | -1.7059 | 244.10 | 342.9062 | 877 | N-(3-Amino-2-hydroxy-phenyl)-acetamide |
| FT601 | 1.6066 | 7.55E-03 | 0.236 | -2.0852 | 244.10 | 441.9053 |  | unindentified |
| FT602 | 1.6112 | 7.01E-03 | 0.078 | -3.679 | 245.10 | 432.8102 |  | unindentified |
| FT604 | 1.6036 | 7.91E-03 | 0.180 | -2.4718 | 245.10 | 441.9198 |  | unindentified |
| FT607 | 1.0241 | NS | 2.9233 | 1.5476 | 246.10 | 553.9181 | 469 | Pyrrolo[1,2-a]pyrazine-1,4-dione, hexahydro-3-(2-methylpropyl)- |
| FT610 | 1.6196 | 6.06E-03 | 0.108 | -3.2128 | 250.10 | 433.8238 | 510 | Chromic acetylacetonate |
| FT613 | 1.6908 | 7.98E-04 | 0.190 | -2.3973 | 257.09 | 441.9198 | 907 | Sucrose, octakis(trimethylsilyl) ether |
| FT638 | 1.6342 | 4.61E-03 | 0.157 | -2.6745 | 271.10 | 441.9160 | 773 | Sucrose, octakis(trimethylsilyl) ether |
| FT641 | 1.5041 | 2.46E-02 | 0.201 | -2.3118 | 272.10 | 441.9160 |  | unindentified |
| FT648 | 1.4110 | 4.80E-02 | 2.4002 | 1.2631 | 281.00 | 552.0428 | 361 | Pyrrolo[1,2-a]pyrazine-1,4-dione, hexahydro-3-(2-methylpropyl)- |
| FT653 | 1.6848 | 1.04E-03 | 5.4042 | 2.4341 | 282.98 | 350.0357 |  | unindentified |
| FT676 | 1.1608 | NS | 2.4691 | 1.304 | 299.09 | 350.1687 |  | unindentified |
| FT678 | 1.1962 | NS | 2.5697 | 1.3616 | 300.01 | 350.0307 |  | unindentified |
| FT679 | 1.1688 | NS | 2.3808 | 1.2515 | 300.08 | 350.2787 |  | unindentified |
| FT680 | 1.1867 | NS | 2.4051 | 1.2661 | 301.03 | 350.1094 |  | unindentified |
| FT681 | 1.1504 | NS | 2.2084 | 1.143 | 301.07 | 350.0588 |  | unindentified |
| FT694 | 1.3042 | NS | 2.5422 | 1.3461 | 313.23 | 504.0042 |  | unindentified |
| FT695 | 1.1380 | NS | 2.1844 | 1.1272 | 314.09 | 350.0588 |  | unindentified |
| FT702 | 1.3746 | NS | 0.156 | -2.6802 | 319.10 | 442.0628 | 829 | α-D-Glucopyranoside, 1,3,4,6-tetrakis-O-(trimethylsilyl)-β-D-fructofuranosyl 2,3,4,6-tetrakis-O-(trimethylsilyl)- |
| FT704 | 1.4852 | 2.88E-02 | 0.108 | -3.2038 | 319.15 | 441.3573 | 907 | Sucrose, octakis(trimethylsilyl) ether |
| FT713 | 1.5206 | 2.12E-02 | 0.183 | -2.4459 | 331.11 | 441.9236 | 929 | α-D-Glucopyranoside, 1,3,4,6-tetrakis-O-(trimethylsilyl)-β-D-fructofuranosyl 2,3,4,6-tetrakis-O-(trimethylsilyl)- |
| FT721 | 1.0133 | NS | 2.5935 | 1.3749 | 337.26 | 531.9327 | 827 | Oleic acid, trimethylsilyl ester |
| FT723 | 1.1851 | NS | 2.5852 | 1.3703 | 339.26 | 531.9411 | 730 | N-(4-Bromo-2,5-dimethylphenyl)-2-chlorobenzamide |
| FT735 | 0.9458 | NS | 0.163 | -2.6203 | 361.13 | 420.0736 | 573 | 2-Amino-1-phenylethanol, trimethylsilyl ether |
| FT736 | 1.4723 | 2.94E-02 | 0.044 | -4.502 | 361.14 | 442.3656 |  | unindentified |
| FT737 | 1.1912 | NS | 0.122 | -3.0296 | 361.20 | 441.9044 |  | unindentified |
| FT738 | 0.7497 | NS | 4.4598 | 2.157 | 361.20 | 485.4167 |  | unindentified |
| FT739 | 0.4233 | NS | 0.477 | -1.0685 | 361.19 | 419.9162 | 889 | 4-Hydroxyphenylethanol, di-TMS |
| FT741 | 1.5074 | 2.39E-02 | 0.060 | -4.0664 | 362.11 | 442.2020 | 829 | α-D-Glucopyranoside, 1,3,4,6-tetrakis-O-(trimethylsilyl)-β-D-fructofuranosyl 2,3,4,6-tetrakis-O-(trimethylsilyl)- |
| FT742 | 0.0307 | NS | 0.432 | -1.2109 | 362.20 | 441.9036 |  | unindentified |
| FT744 | 1.3181 | NS | 0.142 | -2.8149 | 363.11 | 441.9236 | 603 | α-D-Glucopyranoside, 1,3,4,6-tetrakis-O-(trimethylsilyl)-β-D-fructofuranosyl 2,3,4,6-tetrakis-O-(trimethylsilyl)- |
| FT747 | 0.3207 | NS | 0.395 | -1.339 | 363.20 | 441.9044 |  | unindentified |
| FT749 | 1.4584 | 3.52E-02 | 0.153 | -2.7113 | 364.17 | 441.1653 |  | unindentified |
| FT758 | 1.3370 | NS | 0.330 | -1.5999 | 399.30 | 476.0830 |  | unindentified |
| FT764 | 0.7872 | NS | 0.396 | -1.3356 | 437.15 | 442.2019 | 764 | Sucrose, octakis(trimethylsilyl) ether |
| FT766 | 0.7881 | NS | 0.213 | -2.2276 | 437.20 | 441.3501 |  | unindentified |
| FT767 | 1.0308 | NS | 0.150 | -2.7372 | 437.20 | 419.7287 | 598 | 4-Hydroxyphenylethanol, di-TMS |
| FT768 | 1.5054 | 2.44E-02 | 0.303 | -1.7233 | 438.13 | 442.0627 |  | unindentified |
| FT769 | 1.4400 | 3.99E-02 | 0.310 | -1.6904 | 438.21 | 441.3501 | 862 | D-(+)-Trehalose, octakis (trimethylsilyl) ether |
| FT770 | 1.5359 | 1.83E-02 | 0.339 | -1.5617 | 439.15 | 442.2019 |  | unindentified |
| FT771 | 1.1825 | NS | 0.449 | -1.1541 | 439.20 | 441.3501 | 707 | Sucrose, octakis(trimethylsilyl) ether |
| FT774 | 1.5424 | 1.72E-02 | 0.290 | -1.7848 | 451.18 | 441.9236 |  | unindentified |

**Table S9**. Differential metabolites in the FORM15 culture medium inoculated with *Azospirillum brasilense* Ab-V5 after incubation for 36 h and 72 h at 180 rpm and 28 ºC. Peak ID indicates the peak number after XCMS analysis of the chromatograms. VIP score denotes the value of variable importance in projection, considering the component 1 of multivariate supervised discriminant analysis by partial least squares (sPLS-DA). The *p*-value indicates whether a significant difference (p < 0.05) or a non-significant difference (NS) is present according to Student's t-test. FC stands for fold-change analysis, which is the ratio between group means using data prior to column-wise normalization. Log2(FC) indicates the log2 transformed value for each comparison. Match Factor indicates the similarity score of a peak to the listed compounds in the NIST Mass Spectral Library (excellent match: >900; good match: 800–900; fair match: 700–800; medium to low confidence: <700).

| Peak ID | VIP score | *p*-value | FC | log2(FC) | *m/z* | RT (s) | Match Factor | Compounds |
| --- | --- | --- | --- | --- | --- | --- | --- | --- |
| FT001 | 1.4014 | 4.55E-02 | 0.357 | -1.4849 | 44.99 | 348.0756 |  | unindentified |
| FT002 | 0.3327 | NS | 0.419 | -1.255 | 44.99 | 327.0941 |  | unindentified |
| FT003 | 1.3557 | NS | 0.246 | -2.0244 | 44.95 | 359.2051 | 702 | Sym-tetramethyl (diisopropyl)disiloxane |
| FT008 | 1.3736 | NS | 0.326 | -1.6172 | 45.99 | 348.0509 | 808 | Glycerol, tris(trimethylsilyl) ether |
| FT010 | 1.3885 | 4.93E-02 | 0.342 | -1.5466 | 46.95 | 348.0387 | 950 | Glycerol, tris(trimethylsilyl) ether |
| FT011 | 1.3657 | NS | 2.8685 | 1.5203 | 46.95 | 486.0000 |  | unindentified |
| FT015 | 0.8841 | NS | 0.476 | -1.0722 | 49.96 | 300.2195 |  | unindentified |
| FT016 | 0.7975 | NS | 2.2798 | 1.1889 | 50.00 | 300.8238 | 807 | Acetamide, N-(2,6-dimethylphenyl)-N-(2-methylphenyl)- Acetamide, N-(2,6-dimethylphenyl)-N-(2-methylphenyl)- |
| FT025 | 1.1102 | NS | 0.426 | -1.2293 | 57.05 | 404.6882 | 678 | Tyramine, trimethylsilyl ether |
| FT027 | 1.2979 | NS | 0.278 | -1.8466 | 58.01 | 348.0504 | 768 | 2-Phenylamino-6,7-dihydro-5H-1-thia-8-aza-S-indacene-3-carbaldehyde |
| FT030 | 1.4057 | 4.43E-02 | 0.3750 | -1.4147 | 59.00 | 348.1003 |  | unindentified |
| FT031 | 0.4256 | NS | 0.419 | -1.2556 | 58.99 | 327.1555 | 678 | N-Cycloheptyl-2-iodo-benzamide |
| FT036 | 1.3165 | NS | 0.311 | -1.6859 | 60.00 | 348.0504 | 750 | Glycerol, tris(trimethylsilyl) ether |
| FT037 | 1.3590 | NS | 0.412 | -1.2799 | 61.00 | 348.0498 |  | unindentified |
| FT042 | 1.1441 | NS | 0.392 | -1.3497 | 63.00 | 300.1271 | 931 | Benzeneethanamine |
| FT046 | 1.6589 | 1.25E-03 | 0.478 | -1.0636 | 65.05 | 359.0704 | 727 | Sym-tetramethyl (diisopropyl)disiloxane |
| FT051 | 1.2162 | NS | 2.1916 | 1.132 | 70.05 | 495.9961 |  | unindentified |
| FT052 | 1.2027 | NS | 2.6407 | 1.4009 | 70.05 | 477.8932 |  | unindentified |
| FT054 | 1.2602 | NS | 2.9861 | 1.5783 | 70.05 | 458.4049 | 513 | Dimethylketene |
| FT057 | 1.0685 | NS | 0.436 | -1.1984 | 71.05 | 386.9210 | 859 | 1-Mono-isobutyrin, bis(trimethylsilyl)- deriv. |
| FT060 | 1.0920 | NS | 3.7865 | 1.9209 | 72.05 | 478.3067 | 669 | 2,2-Dipropyl-N-ethylpiperidine |
| FT063 | 1.1787 | NS | 0.0142 | -6.1337 | 72.15 | 348.1051 | 924 | Glycerol, tris(trimethylsilyl) ether |
| FT064 | 1.3833 | NS | 3.3812 | 1.7576 | 72.14 | 486.0000 |  | unindentified |
| FT065 | 1.3986 | 4.64E-02 | 0.325 | -1.6202 | 73.05 | 348.0756 |  | unindentified |
| FT068 | 1.4681 | 2.83E-02 | 0.481 | -1.056 | 73.05 | 358.5492 | 910 | 1(2H)-Pentalenone, 3,3a,4,6a-tetrahydro- |
| FT070 | 0.1130 | NS | 0.412 | -1.2778 | 73.05 | 327.1555 | 792 | 3,6,9-Trioxa-2,10-disilaundecane, 2,2,10,10-tetramethyl- |
| FT074 | 1.4749 | 2.68E-02 | 0.323 | -1.6307 | 74.05 | 348.1003 |  | unindentified |
| FT076 | 0.5316 | NS | 0.313 | -1.6772 | 74.05 | 327.1555 | 556 | Pantoyl lactone, trimethylsilyl |
| FT080 | 1.4434 | 3.42E-02 | 2.0735 | 1.0521 | 75.00 | 486.0000 |  | unindentified |
| FT083 | 1.3792 | NS | 0.283 | -1.8219 | 75.04 | 348.0509 |  | unindentified |
| FT084 | 1.0329 | NS | 0.304 | -1.7161 | 75.02 | 532.0743 | 679 | Octadecanoic acid, trimethylsilyl ester |
| FT085 | 1.3369 | NS | 0.367 | -1.4451 | 75.03 | 358.9076 | 727 | Sym-tetramethyl(diisopropyl)disiloxane |
| FT088 | 1.6799 | 4.17E-04 | 2.1836 | 1.1267 | 76.02 | 486.0000 |  | unindentified |
| FT090 | 1.1318 | NS | 0.401 | -1.3199 | 76.03 | 531.9411 | 812 | 9,12-Octadecadienoic acid (Z,Z)-, trimethylsilyl ester |
| FT102 | 0.9512 | NS | 4.9616 | 2.3108 | 85.05 | 486.0000 | 564 | D-(+)-Trehalose, octakis(trimethylsilyl) ether |
| FT103 | 1.4399 | 3.51E-02 | 0.324 | -1.6243 | 85.05 | 348.1603 |  | unindentified |
| FT104 | 1.0646 | NS | 0.380 | -1.3942 | 85.08 | 404.6882 | 678 | Tyramine, trimethylsilyl ether |
| FT113 | 1.3448 | NS | 0.401 | -1.3169 | 87.05 | 348.0382 |  | unindentified |
| FT115 | 1.2442 | NS | 0.297 | -1.751 | 88.05 | 348.0498 |  | unindentified |
| FT116 | 1.0010 | NS | 0.497 | -1.0093 | 88.10 | 486.0066 | 802 | Gulose, 2,3,4,5,6-pentakis-O-(trimethylsilyl)- |
| FT121 | 0.9530 | NS | 4.3872 | 2.1333 | 89.05 | 300.0390 |  | unindentified |
| FT122 | 0.3372 | NS | 0.356 | -1.4911 | 89.05 | 327.1555 |  | unindentified |
| FT134 | 1.4731 | 2.72E-02 | 0.233 | -2.1022 | 91.10 | 358.6306 | 787 | Benzeneacetic acid, trimethylsilyl ester |
| FT135 | 1.6103 | 4.85E-03 | 10.925 | 3.4495 | 91.10 | 353.8015 | 794 | Benzeneethanamine |
| FT136 | 0.9698 | NS | 0.099 | -3.3333 | 91.09 | 484.6124 |  | unindentified |
| FT137 | 0.6013 | NS | 0.043 | -4.5363 | 91.10 | 300.0405 |  | unindentified |
| FT139 | 1.5334 | 1.52E-02 | 7.9596 | 2.9927 | 92.09 | 354.7446 | 748 | Benzeneethanamine |
| FT141 | 1.2416 | NS | 0.090 | -3.4757 | 93.02 | 300.9320 | 807 | Acetamide, N-(2,6-dimethylphenyl)-N-(2-methylphenyl)- |
| FT148 | 1.0107 | NS | 0.249 | -2.0027 | 99.10 | 433.8238 | 758 | Furan, 2,5-dihydro-2,5-dimethoxy- |
| FT149 | 1.3670 | NS | 2.1888 | 1.1301 | 99.10 | 422.0801 | 645 | 4-Amino-4,5(1H)-dihydro-1,2,4-triazole-5-one |
| FT151 | 1.2166 | NS | 10.756 | 3.4271 | 100.08 | 422.2989 | 948 | 4-Amino-4,5(1H)-dihydro-1,2,4-triazole-5-one |
| FT153 | 0.7898 | NS | 0.101 | -3.3087 | 100.09 | 434.1112 |  | unindentified |
| FT156 | 1.4275 | 3.84E-02 | 0.359 | -1.4784 | 101.09 | 348.0509 |  | unindentified |
| FT157 | 0.1477 | NS | 0.325 | -1.619 | 101.09 | 327.0030 | 411 | Butane, 1,2,3-tris(trimethylsiloxy)- |
| FT159 | 1.1664 | NS | 0.499 | -1.0023 | 101.09 | 404.8656 | 662 | Bis(tert-butyldimethylsilyl) 3,6,9,12-tetraoxatetradecane-1,14-dioate |
| FT162 | 1.6130 | 4.59E-03 | 2.6508 | 1.4065 | 102.10 | 486.0000 |  | unindentified |
| FT163 | 0.9950 | NS | 0.383 | -1.3831 | 102.10 | 433.9338 |  | unindentified |
| FT165 | 1.3073 | NS | 0.289 | -1.7896 | 102.14 | 348.0498 |  | unindentified |
| FT166 | 1.4169 | 4.12E-02 | 0.343 | -1.545 | 103.10 | 348.0756 |  | unindentified |
| FT168 | 0.0888 | NS | 0.436 | -1.197 | 103.10 | 434.0925 | 682 | 2-Thiobarbituric acid, tris(tert-butyldimethylsilyl) deriv |
| FT170 | 0.1447 | NS | 0.324 | -1.624 | 103.10 | 327.5430 |  | unindentified |
| FT171 | 1.4012 | 4.56E-02 | 0.440 | -1.1842 | 104.10 | 348.1003 |  | unindentified |
| FT177 | 1.1211 | NS | 0.409 | -1.2906 | 105.10 | 344.0783 | 867 | Benzoic acid trimethylsilyl ester |
| FT182 | 1.6517 | 1.64E-03 | 3.8567 | 1.9474 | 107.10 | 495.9923 | 766 | Tyrosine |
| FT186 | 1.6009 | 5.82E-03 | 3.8009 | 1.9263 | 107.10 | 478.9640 | 682 | Glycyl-L-tyrosine |
| FT193 | 1.2553 | NS | 2.3649 | 1.2418 | 110.10 | 468.2889 |  | unindentified |
| FT195 | 1.0245 | NS | 2.4466 | 1.2908 | 111.10 | 485.9875 | 530 | Ethyl 2,3,4,6-tetrakis-O-(trimethylsilyl)-D-glucopyranoside |
| FT196 | 1.2738 | NS | 2.5579 | 1.355 | 111.10 | 468.2557 | 727 | 2,4-Imidazolidinedione, 5-methyl- |
| FT198 | 1.3950 | 4.74E-02 | 0.264 | -1.9206 | 113.05 | 348.0509 |  | unindentified |
| FT199 | 1.2199 | NS | 2.3007 | 1.2021 | 113.10 | 459.4251 | 544 | Acetaldehyde, ethyl(1-methylethyl)hydrazone |
| FT200 | 1.3423 | NS | 0.288 | -1.7947 | 113.10 | 348.0380 | 727 | 2(3H)-Furanone, 3-[(4-nitrophenyl)methylene]-5-phenyl- |
| FT201 | 0.6663 | NS | 2.2593 | 1.1758 | 113.10 | 486.0000 |  | unindentified |
| FT202 | 1.1914 | NS | 2.0752 | 1.0532 | 113.09 | 495.1311 |  | unindentified |
| FT204 | 1.2061 | NS | 2.9488 | 1.5601 | 114.09 | 467.4453 | 649 | Tryptophol |
| FT205 | 1.1618 | NS | 2.5603 | 1.3563 | 114.08 | 449.1627 | 598 | 3-Methyl-4-amino-4,5(1H)-dihydro-1,2,4-triazole-5-one |
| FT210 | 1.3491 | NS | 0.279 | -1.8394 | 115.06 | 348.0265 |  | unindentified |
| FT211 | 1.4869 | 2.41E-02 | 2.6332 | 1.3968 | 115.10 | 486.0714 |  | unindentified |
| FT213 | 0.1496 | NS | 0.306 | -1.7079 | 116.10 | 327.0941 |  | unindentified |
| FT216 | 1.3030 | NS | 0.247 | -2.0163 | 116.14 | 348.1056 | 934 | Silanol, trimethyl-, phosphate (3:1) |
| FT217 | 1.4058 | 4.43E-02 | 0.321 | -1.6371 | 117.10 | 348.0756 |  | unindentified |
| FT219 | 0.2124 | NS | 0.408 | -1.2917 | 117.10 | 327.0327 |  | unindentified |
| FT220 | 0.8715 | NS | 0.360 | -1.4722 | 117.10 | 531.9411 | 371 | N-Cyclohexyl-1-pyrrolidinecarbothioamide |
| FT221 | 1.4718 | 2.75E-02 | 0.383 | -1.3829 | 118.08 | 348.2789 |  | unindentified |
| FT223 | 1.0878 | NS | 0.403 | -1.3101 | 118.10 | 532.9350 | 513 | 1,2-Cyclohexanedicarboxylic acid, 2,6-dimethoxyphenyl heptyl ester |
| FT224 | 0.0898 | NS | 0.418 | -1.258 | 118.10 | 326.9425 | 698 | 3,6,9-Trioxa-2,10-disilaundecane, 2,2,10,10-tetramethyl- |
| FT225 | 0.9518 | NS | 0.334 | -1.5804 | 118.10 | 348.0262 |  | unindentified |
| FT226 | 1.3983 | 4.65E-02 | 0.280 | -1.8353 | 119.06 | 348.1003 |  | unindentified |
| FT229 | 1.3046 | NS | 4.8938 | 2.2909 | 120.10 | 479.8752 | 660 | 4-Pyridinecarbonitrile, 1-oxide |
| FT239 | 1.3022 | NS | 3.8087 | 1.9293 | 125.10 | 340.0211 | 816 | (Methoxymethyl)trimethylsilane |
| FT247 | 1.1973 | NS | 2.4985 | 1.3211 | 128.10 | 459.4588 | 703 | d-Proline, N-methoxycarbonyl-, isohexyl ester |
| FT250 | 1.3490 | NS | 0.229 | -2.124 | 129.10 | 348.0504 | 750 | Glycerol, tris(trimethylsilyl) ether |
| FT252 | 1.0624 | NS | 0.385 | -1.3766 | 129.10 | 532.0743 |  | unindentified |
| FT257 | 0.7937 | NS | 2.0629 | 1.0446 | 130.10 | 514.9975 | 363 | Myo-Inositol, 1,2,3,4,5,6-hexakis-O-(trimethylsilyl)- |
| FT258 | 1.3547 | NS | 0.298 | -1.7478 | 130.09 | 347.9841 | 948 | Glycerol, tris(trimethylsilyl) ether |
| FT259 | 1.4329 | 3.69E-02 | 3.9934 | 1.9976 | 130.10 | 469.3359 | 703 | Alanine, N-methyl-N-ethoxycarbonyl-, dodecyl ester |
| FT260 | 1.5295 | 1.59E-02 | 2.8873 | 1.5297 | 130.09 | 425.8345 | 828 | Benzenepropanoic acid, α-[(trimethylsilyl)oxy]-, trimethylsilyl ester |
| FT262 | 1.3932 | 4.79E-02 | 0.218 | -2.194 | 131.04 | 348.0509 |  | unindentified |
| FT263 | 1.2935 | NS | 2.0280 | 1.0201 | 131.09 | 486.0000 |  | unindentified |
| FT264 | 1.4065 | 4.41E-02 | 0.361 | -1.4707 | 131.06 | 348.1050 |  | unindentified |
| FT265 | 0.0727 | NS | 0.434 | -1.2042 | 131.08 | 327.1555 | 735 | Pantoyl lactone, trimethylsilyl |
| FT269 | 1.4344 | 3.65E-02 | 0.334 | -1.580 | 132.06 | 348.0265 |  | unindentified |
| FT270 | 0.3311 | NS | 0.421 | -1.2485 | 132.07 | 327.0941 |  | unindentified |
| FT274 | 1.0655 | NS | 0.463 | -1.1096 | 132.10 | 532.9350 | 740 | Octadecanoic acid, trimethylsilyl ester |
| FT275 | 1.3275 | NS | 0.320 | -1.6424 | 132.13 | 347.9841 | 790 | Glycerol, tris(trimethylsilyl) ether |
| FT276 | 1.3899 | 4.89E-02 | 0.328 | -1.6086 | 133.05 | 348.0756 |  | unindentified |
| FT277 | 1.6814 | 3.75E-04 | 2.7723 | 1.4711 | 133.06 | 486.0000 |  | unindentified |
| FT278 | 0.3757 | NS | 0.395 | -1.3411 | 133.05 | 327.0327 |  | unindentified |
| FT281 | 1.3205 | NS | 0.246 | -2.0217 | 134.05 | 348.0509 |  | unindentified |
| FT284 | 1.0703 | NS | 0.416 | -1.2667 | 134.10 | 388.0871 | 724 | Silane, trimethyl (tricyclo[3.3.1.1(3,7)]dec-2-yloxy)- |
| FT287 | 1.0830 | NS | 2.0243 | 1.0174 | 135.08 | 469.3414 | 704 | Adenine |
| FT290 | 1.5053 | 2.04E-02 | 0.426 | -1.2298 | 137.08 | 359.1714 | 727 | Sym-tetramethyl(diisopropyl)disiloxane |
| FT295 | 1.4773 | 2.62E-02 | 2.1044 | 1.0734 | 140.08 | 512.1827 |  | unindentified |
| FT304 | 1.4454 | 3.37E-02 | 2.2951 | 1.1986 | 143.08 | 507.0563 |  | unindentified |
| FT309 | 1.2768 | NS | 0.446 | -1.1659 | 145.10 | 532.0743 | 371 | N-Cyclohexyl-1-pyrrolidinecarbothioamide |
| FT314 | 1.3248 | NS | 0.282 | -1.8257 | 146.14 | 348.0498 |  | unindentified |
| FT317 | 1.4063 | 4.41E-02 | 0.313 | -1.6753 | 147.10 | 348.1003 |  | unindentified |
| FT321 | 1.1103 | NS | 0.417 | -1.2632 | 147.09 | 405.2682 | 678 | Tyramine, trimethylsilyl ether |
| FT322 | 1.4091 | 4.34E-02 | 0.330 | -1.6005 | 148.09 | 348.0756 |  | unindentified |
| FT324 | 0.6996 | NS | 0.335 | -1.5787 | 148.06 | 327.1555 |  | unindentified |
| FT330 | 1.4308 | 3.75E-02 | 0.330 | -1.5977 | 149.05 | 348.1056 |  | unindentified |
| FT333 | 0.7124 | NS | 0.280 | -1.8341 | 149.10 | 332.0562 | 716 | 2-Ethyl-2-phenyl-1,3-benzodioxole |
| FT334 | 1.3103 | NS | 0.326 | -1.6176 | 150.07 | 348.0504 |  | unindentified |
| FT345 | 1.2116 | NS | 2.7192 | 1.4432 | 154.08 | 477.7985 | 514 | 6-Cyanoquinoline |
| FT347 | 1.4859 | 2.43E-02 | 2.9911 | 1.5807 | 155.04 | 486.0000 |  | unindentified |
| FT352 | 1.5226 | 1.71E-02 | 2.1893 | 1.1305 | 156.08 | 453.1774 | 412 | Benzenemethanol, α-(chloromethyl)- |
| FT357 | 1.4642 | 2.92E-02 | 0.268 | -1.9014 | 159.05 | 348.0262 | 892 | 1-(1-Adamantyl)-2-triisopropylsilyloxyethane |
| FT358 | 1.1274 | NS | 0.423 | -1.2414 | 159.09 | 404.7100 | 678 | Tyramine, trimethylsilyl ether |
| FT359 | 1.4536 | 3.17E-02 | 0.346 | -1.5319 | 159.10 | 348.3706 |  | unindentified |
| FT365 | 1.3870 | 4.98E-02 | 0.277 | -1.850 | 163.05 | 348.0382 |  | unindentified |
| FT369 | 1.2499 | NS | 0.329 | -1.6055 | 164.10 | 358.1909 | 766 | Sym-tetramethyl (diisopropyl)disiloxane |
| FT379 | 1.4188 | 4.07E-02 | 2.9549 | 1.5631 | 168.07 | 512.1234 | 352 | 9H-Pyrido[3,4-b]indole |
| FT380 | 1.1601 | NS | 2.1854 | 1.1279 | 168.10 | 458.0905 | 544 | 3-Ethoxy-4-methoxyphenol |
| FT383 | 1.4123 | 4.25E-02 | 4.7315 | 2.2423 | 170.10 | 478.0498 | 682 | Glycyl-L-tyrosine |
| FT387 | 1.6251 | 3.51E-03 | 2.4217 | 1.276 | 171.10 | 486.0000 |  | unindentified |
| FT396 | 1.3823 | NS | 0.266 | -1.9124 | 175.05 | 348.0504 |  | unindentified |
| FT400 | 1.5536 | 1.20E-02 | 3.0173 | 1.5933 | 176.10 | 486.0000 |  | unindentified |
| FT403 | 1.3931 | 4.80E-02 | 0.201 | -2.3137 | 177.05 | 348.0387 |  | unindentified |
| FT404 | 1.4345 | 3.65E-02 | 0.334 | -1.5822 | 177.08 | 348.1003 |  | unindentified |
| FT407 | 1.4085 | 4.35E-02 | 0.344 | -1.5408 | 178.06 | 348.0382 |  | unindentified |
| FT411 | 0.8500 | NS | 5.5295 | 2.4672 | 179.03 | 347.5509 |  | unindentified |
| FT412 | 1.1465 | NS | 0.423 | -1.2426 | 179.05 | 344.0099 | 776 | Benzeneethanamine |
| FT416 | 0.0675 | NS | 0.463 | -1.112 | 179.10 | 487.8874 |  | unindentified |
| FT420 | 1.6727 | 6.55E-04 | 0.396 | -1.336 | 180.10 | 407.4287 | 717 | Silane, dimethyl-2-propenyl(tetradecyloxy)- |
| FT422 | 1.4769 | 2.63E-02 | 0.256 | -1.9638 | 180.11 | 409.6467 | 701 | Tyramine, trimethylsilyl ether |
| FT433 | 1.3606 | NS | 0.314 | -1.6725 | 189.05 | 348.0264 |  | unindentified |
| FT437 | 1.4417 | 3.47E-02 | 3.8042 | 1.9276 | 190.09 | 486.0000 |  | unindentified |
| FT443 | 1.4797 | 2.57E-02 | 0.301 | -1.7295 | 191.05 | 348.0756 |  | unindentified |
| FT446 | 1.3390 | NS | 0.220 | -2.1809 | 191.09 | 348.1984 |  | unindentified |
| FT449 | 1.3436 | NS | 0.338 | -1.5666 | 192.08 | 348.0504 |  | unindentified |
| FT453 | 1.5765 | 8.72E-03 | 0.495 | -1.0144 | 193.08 | 359.0704 | 887 | Benzeneacetic acid, trimethylsilyl ester |
| FT458 | 1.2521 | NS | 2.223 | 1.1525 | 194.09 | 502.0036 |  | unindentified |
| FT467 | 1.6068 | 5.20E-03 | 3.0297 | 1.5992 | 199.07 | 486.0000 |  | unindentified |
| FT468 | 1.5108 | 1.93E-02 | 5.127 | 2.3581 | 199.07 | 447.0001 | 676 | D-Turanose, heptakis(trimethylsilyl)- |
| FT477 | 1.3004 | NS | 0.211 | -2.2465 | 203.09 | 348.0509 | 529 | Silanol, trimethyl-, phosphate (3:1) |
| FT480 | 1.0747 | NS | 0.469 | -1.0916 | 203.09 | 404.7100 | 678 | Tyramine, trimethylsilyl ether |
| FT481 | 1.1716 | NS | 0.472 | -1.0817 | 203.10 | 387.3405 | 893 | 1-Mono-isobutyrin, bis(trimethylsilyl)- deriv. |
| FT483 | 1.3251 | NS | 0.173 | -2.5337 | 203.10 | 348.1618 | 757 | Glycerol, tris(trimethylsilyl) ether |
| FT484 | 1.4679 | 2.84E-02 | 2.7308 | 1.4493 | 204.09 | 509.0190 | 525 | Phenylthioacetic acid, 4-cyanophenyl ester |
| FT485 | 1.1542 | NS | 2.4333 | 1.2829 | 204.10 | 486.0000 |  | unindentified |
| FT486 | 1.3233 | NS | 0.185 | -2.4343 | 204.10 | 348.0387 | 774 | Silane, trimethyl (propoxymethyl)- |
| FT487 | 1.5582 | 1.13E-02 | 2.6156 | 1.3872 | 204.11 | 508.7986 | 751 | Gulose, 2,3,4,5,6-pentakis-O-(trimethylsilyl)- |
| FT488 | 1.3472 | NS | 0.179 | -2.4784 | 204.13 | 348.3706 | 757 | Glycerol, tris(trimethylsilyl) ether |
| FT489 | 1.5761 | 8.77E-03 | 5.1953 | 2.3772 | 205.10 | 486.0000 |  | unindentified |
| FT490 | 0.1245 | NS | 0.175 | -2.5107 | 205.10 | 327.1555 |  | unindentified |
| FT491 | 1.4378 | 3.57E-02 | 0.290 | -1.7857 | 205.10 | 348.1303 |  | unindentified |
| FT492 | 1.1770 | NS | 2.3729 | 1.2466 | 206.10 | 486.0000 |  | unindentified |
| FT493 | 1.4223 | 3.97E-02 | 0.305 | -1.711 | 206.10 | 348.1303 | 551 | 2-Octen-1-ol |
| FT496 | 1.5245 | 1.68E-02 | 0.342 | -1.5482 | 206.11 | 348.0380 |  | unindentified |
| FT498 | 1.0362 | NS | 0.497 | -1.0092 | 207.04 | 552.1802 | 351 | Acetonitrile, (dimethylamino)- |
| FT501 | 1.3126 | NS | 0.231 | -2.1165 | 207.09 | 348.0264 |  | unindentified |
| FT503 | 1.3143 | NS | 0.343 | -1.5425 | 208.08 | 348.0504 |  | unindentified |
| FT519 | 1.2569 | NS | 2.616 | 1.3874 | 217.10 | 486.0000 |  | unindentified |
| FT520 | 1.1393 | NS | 2.1327 | 1.0927 | 217.10 | 457.2807 | 720 | Silane, diethylethoxyoctyloxy- |
| FT522 | 1.5007 | 2.13E-02 | 2.4172 | 1.2734 | 217.10 | 508.9257 | 601 | β-Gentiobiose, octakis(trimethylsilyl) ether |
| FT526 | 1.3516 | NS | 0.198 | -2.3353 | 217.10 | 348.0386 |  | unindentified |
| FT528 | 1.6149 | 4.42E-03 | 4.9241 | 2.2999 | 218.10 | 486.0000 |  | unindentified |
| FT530 | 1.3759 | NS | 2.0648 | 1.046 | 218.09 | 456.8384 | 755 | D-(-)-Ribofuranose, tetrakis (trimethylsilyl) ether (isomer 2) |
| FT531 | 1.4400 | 3.51E-02 | 0.284 | -1.8154 | 218.10 | 348.0756 |  | unindentified |
| FT533 | 1.0565 | NS | 0.375 | -1.4143 | 219.09 | 348.1056 | 556 | Benzene, 1,1'-[1-(2-propenyl)-1,2-ethanediyl]bis- |
| FT536 | 1.2376 | NS | 2.0889 | 1.0628 | 219.10 | 486.0000 |  | unindentified |
| FT538 | 1.3500 | NS | 0.184 | -2.4395 | 219.13 | 348.0498 |  | unindentified |
| FT539 | 1.3712 | NS | 0.224 | -2.1555 | 220.09 | 348.0265 |  | unindentified |
| FT544 | 1.3894 | 4.91E-02 | 0.323 | -1.6317 | 221.08 | 348.0380 |  | unindentified |
| FT550 | 0.7161 | NS | 0.472 | -1.0842 | 223.10 | 458.9928 | 518 | 1,4-Dioxaspiro[4.5]decane, 8-chloro- |
| FT559 | 1.1935 | NS | 2.3632 | 1.2407 | 229.10 | 486.0000 |  | unindentified |
| FT563 | 1.0876 | NS | 2.1417 | 1.0987 | 230.10 | 442.9854 | 717 | 3-Tetradecene, (Z)- |
| FT565 | 1.2160 | NS | 2.2027 | 1.1393 | 231.10 | 486.0000 |  | unindentified |
| FT571 | 0.0349 | NS | 0.392 | -1.3511 | 231.15 | 433.9338 | 685 | Benzenamine, N,N'-(1,2-dimethyl-1,2-ethanediylidene)bis[2,6-bis(1-methylethyl)- |
| FT578 | 1.6255 | 3.48E-03 | 2.5362 | 1.3427 | 233.11 | 486.0000 |  | unindentified |
| FT598 | 1.4430 | 3.44E-02 | 5.9421 | 2.571 | 244.10 | 342.9062 | 877 | N-(3-Amino-2-hydroxy-phenyl)-acetamide |
| FT611 | 1.6158 | 4.33E-03 | 0.319 | -1.6465 | 251.09 | 482.0141 | 741 | β-D-Mannopyranoside, methyl 2,3,4,6-tetrakis-O-(trimethylsilyl)- |
| FT622 | 0.5653 | NS | 0.377 | -1.4061 | 262.15 | 504.4969 | 498 | Phenylthioacetic acid, 4-cyanophenyl ester |
| FT637 | 0.8722 | NS | 2.1505 | 1.1047 | 271.09 | 485.7143 |  | unindentified |
| FT648 | 1.6226 | 3.72E-03 | 0.379 | -1.3995 | 281.00 | 552.0428 | 361 | Pyrrolo[1,2-a]pyrazine-1,4-dione, hexahydro-3-(2-methylpropyl)- |
| FT653 | 0.8587 | NS | 0.255 | -1.9717 | 282.98 | 350.0357 |  | unindentified |
| FT658 | 1.5782 | 8.50E-03 | 12.256 | 3.6154 | 289.10 | 447.0771 | 648 | Ribitol, 1,2,3,4,5-pentakis-O-(trimethylsilyl)- |
| FT660 | 1.6162 | 4.29E-03 | 6.8566 | 2.7775 | 289.10 | 486.0000 | 832 | Ethyl 2,3,4,6-tetrakis-O-(trimethylsilyl)-D-glucopyranoside |
| FT662 | 1.5077 | 1.99E-02 | 11.083 | 3.4703 | 289.13 | 445.8668 | 634 | D-(+)-Trehalose, octakis(trimethylsilyl) ether |
| FT665 | 1.5857 | 7.56E-03 | 7.530 | 2.9126 | 290.10 | 446.8869 | 703 | 3,6-Dioxa-2,7-disilaoctane, 2,2,4,7,7-pentamethyl- |
| FT668 | 1.4755 | 2.66E-02 | 3.4134 | 1.7712 | 291.10 | 486.0000 |  | unindentified |
| FT671 | 1.4974 | 2.20E-02 | 0.345 | -1.5365 | 293.09 | 348.0065 |  | unindentified |
| FT685 | 1.4114 | 4.27E-02 | 3.6424 | 1.8649 | 305.10 | 486.0000 |  | unindentified |
| FT702 | 1.4652 | 2.90E-02 | 9.4774 | 3.2445 | 319.10 | 442.0628 | 829 | α-D-Glucopyranoside, 1,3,4,6-tetrakis-O-(trimethylsilyl)-β-D-fructofuranosyl 2,3,4,6-tetrakis-O-(trimethylsilyl)- |
| FT712 | 1.2842 | NS | 3.0022 | 1.586 | 331.14 | 485.8571 | 810 | Mannose, 6-deoxy-2,3,4,5-tetrakis-O-(trimethylsilyl)-, L- |
| FT713 | 1.6776 | 4.85E-04 | 11.071 | 3.4687 | 331.11 | 441.9236 | 929 | α-D-Glucopyranoside, 1,3,4,6-tetrakis-O-(trimethylsilyl)-β-D-fructofuranosyl 2,3,4,6-tetrakis-O-(trimethylsilyl)- |
| FT720 | 1.4023 | 4.52E-02 | 0.291 | -1.7787 | 337.20 | 532.0506 | 634 | Tetrasiloxane, 3,5-diethoxy-1,1,1,7,7,7-hexamethyl-3,5-bis(trimethylsiloxy)- |
| FT721 | 1.3141 | NS | 0.320 | -1.6413 | 337.26 | 531.9327 | 827 | Oleic acid, trimethylsilyl ester |
| FT723 | 1.3061 | NS | 0.326 | -1.6149 | 339.26 | 531.9411 | 730 | N-(4-Bromo-2,5-dimethylphenyl)-2-chlorobenzamide |
| FT725 | 1.4054 | 4.44E-02 | 0.392 | -1.3496 | 341.00 | 546.6301 | 360 | 1-Silacyclo-2,4-hexadiene |
| FT726 | 1.1208 | NS | 0.442 | -1.1789 | 341.15 | 533.9179 | 363 | D:A-Friedoolean-3-en-29-oic acid, methyl ester, (20α)- |
| FT727 | 1.2923 | NS | 0.254 | -1.9777 | 341.15 | 530.7080 | 668 | 9,12-Octadecadienoic acid (Z,Z)-, trimethylsilyl ester |
| FT734 | 1.4973 | 2.20E-02 | 10.493 | 3.3914 | 361.10 | 485.3125 | 564 | D-(+)-Trehalose, octakis(trimethylsilyl) ether |
| FT736 | 1.1927 | NS | 161.960 | 7.3395 | 361.14 | 442.3656 |  | unindentified |
| FT739 | 0.1663 | NS | 0.277 | -1.8494 | 361.19 | 419.9162 | 889 | 4-Hydroxyphenylethanol, di-TMS |
| FT742 | 0.1810 | NS | 0.369 | -1.4371 | 362.20 | 441.9036 |  | unindentified |
| FT747 | 0.2567 | NS | 0.354 | -1.4972 | 363.20 | 441.9044 |  | unindentified |
| FT750 | 1.5629 | 1.06E-02 | 4.690 | 2.2296 | 365.15 | 446.9551 | 648 | Ribitol, 1,2,3,4,5-pentakis-O-(trimethylsilyl)- |
| FT751 | 1.4102 | 4.31E-02 | 5.6071 | 2.4873 | 371.26 | 407.0411 | 796 | Silane, diethyldodecyloxyoctyloxy- |
| FT764 | 0.9827 | NS | 2.6012 | 1.3792 | 437.15 | 442.2019 | 764 | Sucrose, octakis(trimethylsilyl) ether |
| FT769 | 1.2536 | NS | 0.380 | -1.3949 | 438.21 | 441.3501 | 862 | D-(+)-Trehalose, octakis (trimethylsilyl) ether |
